# An Alliance of Gel-Based and Gel-Free Proteomic Techniques Displays Substantial Insight Into the Proteome of a Virulent and an Attenuated *Histomonas meleagridis* Strain

**DOI:** 10.3389/fcimb.2018.00407

**Published:** 2018-11-16

**Authors:** Andreas Monoyios, Karin Hummel, Katharina Nöbauer, Martina Patzl, Sarah Schlosser, Michael Hess, Ivana Bilic

**Affiliations:** ^1^Clinic for Poultry and Fish Medicine, Department for Farm Animals and Veterinary Public Health, University of Veterinary Medicine Vienna, Vienna, Austria; ^2^VetCore Facility for Research, University of Veterinary Medicine Vienna, Vienna, Austria; ^3^Department for Pathobiology, Institute of Immunology, University of Veterinary Medicine Vienna, Vienna, Austria; ^4^Christian Doppler Laboratory for Innovative Poultry Vaccines, University of Veterinary Medicine Vienna, Vienna, Austria

**Keywords:** *Histomonas meleagridis*, protozoa, 2D-DIGE, SWATH MS, attenuation, virulence factors, comparative proteomic analysis

## Abstract

The unicellular protozoan *Histomonas meleagridis* is notorious for being the causative agent of histomonosis, which can cause high mortality in turkeys and substantial production losses in chickens. The complete absence of commercially available curative strategies against the disease renders the devising of novel approaches a necessity. A fundamental step toward this objective is to understand the flagellate's virulence and attenuation mechanisms. For this purpose we have previously conducted a comparative proteomic analysis of an *in vitro* cultivated virulent and attenuated histomonad parasite using two-dimensional electrophoresis and MALDI-TOF/TOF. The current work aimed to substantially extend the knowledge of the flagellate's proteome by applying 2D-DIGE and sequential window acquisition of all theoretical mass spectra (SWATH) MS as tools on the two well-defined strains. In the gel-based experiments, 49 identified protein spots were found to be differentially expressed, of which 37 belonged to the *in vitro* cultivated virulent strain and 12 to the attenuated one. The most frequently identified proteins in the virulent strain take part in cytoskeleton formation, carbohydrate metabolism and adaptation to stress. However, post-translationally modified or truncated ubiquitous cellular proteins such as actin and GAPDH were identified as upregulated in multiple gel positions. This indicated their contribution to processes not related to cytoskeleton and carbohydrate metabolism, such as fibronectin or plasminogen binding. Proteins involved in cell division and cytoskeleton organization were frequently observed in the attenuated strain. The findings of the gel-based studies were supplemented by the gel-free SWATH MS analysis, which identified and quantified 42 significantly differentially regulated proteins. In this case proteins with peptidase activity, metabolic proteins and actin-regulating proteins were the most frequent findings in the virulent strain, while proteins involved in hydrogenosomal carbohydrate metabolism dominated the results in the attenuated one.

## Introduction

The unicellular microaerophilic poultry parasite *Histomonas meleagridis*, first classified in 1920 (Tyzzer, 1919), is a member of the order *Tritrichomonadida* (Cepicka et al., 2010). In gallinaceous birds, the flagellate is responsible for histomonosis, also known as infectious enterohepatitis or blackhead disease (reviewed in Hess, 2017). In the course of the disease the parasite invades the caecum, then it gains access to the circulatory system through which it travels to the liver and other organs. The necrosis of the liver, which is frequently seen in turkeys, can lead to high mortality (reviewed in McDougald, 2005). In chickens, this gross pathological finding is less common, but severe clinical signs including significant drop in egg production were recorded (Hess and McDougald, 2013). In recent years histomonosis re-emerged, due to the withdrawal of previously effective anti-histomonad drugs and the gaining popularity of free-range poultry farming (reviewed in Hess et al., 2015).

The establishment of a mono-eukaryotic *H. meleagridis* culture derived from a single cell and its successful stable attenuation after numerous *in vitro* passages opened the road for the development of innovative strategies against the flagellate (Hess et al., 2006; Sulejmanovic et al., 2013). Such attenuated parasites which are propagated *in vitro* in a mixed bacterial content, termed as xenic culture, were proven to be safe and effective as a vaccine (Liebhart et al., 2011, 2013). Among the known prevention and therapeutic approaches that have been considered against *H. meleagridis*, live attenuated vaccination was recognized to be the most promising, albeit not yet commercially available (reviewed in Liebhart et al., 2017). The further development of the mono-eukaryotic culture by replacing the mixed bacterial flora with *Escherichia coli* DH5α, showed no effect on the attenuation process (Ganas et al., 2012) and was an important tool for detailed molecular studies.

Despite its importance for poultry health only limited knowledge is available on the parasite's molecular biology, a precondition to develop new protection strategies or improve existing ones. A number of molecular studies determined the phylogenetic relationship of *H. meleagridis* and enriched the collection of available sequences (reviewed in Hess et al., 2015). A non-comparative two-dimensional electrophoresis (2-DE) study on *H. meleagridis* proteome revealed 17 out of 19 identified proteins as actin (Pham et al., 2016). A recent comparative 2-DE proteome analysis of the virulent and attenuated *H. meleagridis* culture, identified 49 significantly differentially expressed protein spots between the two strains, corresponding to 31 unique *H. meleagridis* proteins (Monoyios et al., 2018). In that study, mass spectrometric identification of significant protein spots was supported by the recent *de novo* transcriptome sequencing of the virulent and attenuated parasite, which was the latest contribution toward the enrichment of *H. meleagridis* sequence data (Mazumdar et al., 2017).

As the evolution in mass spectrometric identification methods and gel image analysis continued, the popularity of 2-DE has risen and the technique was favored for the separation of complete proteins at high resolution (reviewed in Rabilloud et al., 2010). The introduction of immobilized pH gradient (IPG) gel strips and a new 2-DE protocol, offered the advantage of reproducibility (Görg et al., 2009). However, with conventional 2-DE, only one protein sample can be visualized per gel, while reproducibility issues remained due to the gel-to-gel variation (reviewed in Miller, 2012). From this aspect, the two-dimensional differential gel electrophoresis (2D-DIGE) was considered to be an improvement of 2-DE since it allows for the resolution of three different samples in parallel on the same gel, which are labeled with different fluorescent dyes (reviewed in Miller, 2012). These can include two protein samples to be compared and an internal standard (IS). The latter is used to quantitatively normalize all protein spots, which are included in the experiment, a feature that increases accuracy in detecting differential protein expression (reviewed in Baggerman et al., 2005). In addition, the sensitivity of the 2-DE silver staining protocol and 2D-DIGE minimal labeling are unparalleled since the former can detect 1 ng of protein per 2D spot and the latter as low as 0.1 ng of protein per 2D spot (reviewed in Sitek et al., 2006).

Nevertheless, from all the proteins present in a complex sample, only 30–50% can be resolved in a 2-DE gel depending on their abundance and physicochemical properties (reviewed in Baggerman et al., 2005). As a consequence the same group of proteins will be repeatedly visualized and a “déjà vu” of reported results, can be expected (Petrak et al., 2008). Some of the limitations of gel-based methods can be tackled by shotgun proteomic techniques that have the capability of identifying over 10,000 proteins in a sample through a data-dependent approach (Huang et al., 2015). Nonetheless, even with the current technology, challenges arise when reproducible, sensitive, and accurate quantification is required among multiple samples that cover wider proportions of the proteome (Gillet et al., 2012). Acquisition of MS/MS data by the Triple-TOF high-resolution mass spectrometer allowed the development of a new quantification approach called sequential window acquisition of all theoretical mass spectra (SWATH), which is a data-independent acquisition method with the aim of analyzing the fragmentation products of all ions (reviewed in Crutchfield et al., 2016). Label-free quantification of protein abundance with SWATH technology demonstrated high precision, reproducibility, and accuracy (Huang et al., 2015). The usefulness of this approach was recently demonstrated in the comparative proteomic analysis of a fish bacterial pathogen, with the prospect of comprehending its virulence mechanisms (Kumar et al., 2016). However, to the extent of our knowledge very few, if any, comparative proteomic studies on protozoa have utilized the SWATH MS technology.

In the present study, 2D-DIGE gel-based experiments were complemented by the gel-free, label-free proteomic technique which involved nano-scale liquid chromatography coupled with a hybrid high-resolution mass spectrometer and SWATH MS technology. The main objective was to resolve proteome differences or similarities between the *in vitro* cultivated virulent and attenuated *H. meleagridis* strains in greater detail in a comprehensive setting.

## Materials and methods

### Cultivation and collection of virulent and attenuated *H. meleagridis* parasites

The experiments were performed using virulent and attenuated *H. meleagridis* parasites which were propagated *in vitro* as monoxenic mono-eukaryotic cultures designated as *H. meleagridis/turkey/Austria/2922-C6/04-10x/DH5*α *and H. meleagridis/turkey/Austria/2922-C6/04-290x/DH5*α, respectively (Ganas et al., 2012). The incubation took place at 41°C in a standard medium consisting of Medium 199 (Gibco™, Vienna, Austria), 15% heat-inactivated fetal bovine serum (Gibco™), and 0.22% of sterilized rice starch (Carl Roth GmbH + Co. KG, Karlsruhe, Germany). Parasites were passaged every 3 days (Ganas et al., 2012) and at the final passage 1 ml of *E. coli* DH5α liquid culture (colony forming units (CFU): 5 × 10^8^ bacterial cells/ml), which was grown overnight at 37°C, was added to the cultures. Subsequently, the virulent low-passaged parasites were harvested at passage number 25 and the attenuated high-passaged parasites at passage number 303. A purification protocol consisting of consecutive washing and centrifugation steps was used to collect *H. meleagridis* cells and to remove most of the bacteria as previously described (Monoyios et al., 2018). Pellets of collected parasites were stored at −80°C until further use. The quality of purification and parasite numbers were evaluated using Trypan Blue Stain (Gibco™, Invitrogen, Vienna, Austria) as previously described (Zaragatzki et al., 2010).

### Protein sample preparation for 2D-DIGE and SWATH MS

In total four biological replicates and four technical replicates were used in the 2D-DIGE experiment. For each sample protein was extracted from 6 × 10^7^ purified parasites, which were co-cultivated in tissue culture flasks with *E. coli* DH5α. Four separate virulent and attenuated *H. meleagridis* cultures were harvested on the same day at passage number 25 and 303, respectively (Figure S1). Two parasite cultures from each strain were pooled together prior to the protein extraction resulting in two samples for the virulent strain 25a and 25b and two for the attenuated strain 303a and 303b (Figure S1). In that way, a higher number of parasites was obtained and at the same time higher biological variability was introduced in each sample. In order to obtain a second group of four protein samples (25a, 25b, 303a, 303b), the cultivation and the extraction schemes from above were repeated using *H. meleagridis* parasites, which were cultivated and harvested at a different time point. For both experiments, protein extraction of the first biological replicate was performed on the day 1 (sample a) and the second biological replicate on the day 2 (sample b), and as a result four protein samples were included in each of the two 2D-DIGE experiments: 25a, 25b, 303a, 303b (Figure S1). In each 2D-DIGE experiment, each of the two biological replicates of a given strain (e.g., 25a and 25b) was swap labeled with the fluorescent dyes (e.g., 25a with G-Dye200 and 25a with G-Dye300, Table 1) and the acquired fluorescent images represented technical replicates for the 2D-DIGE protocol (Figure S1). The protein sample preparation procedure, described below, was applied to all eight samples. Two pellets of collected parasites were resuspended in extraction buffer [50 mM Tris-HCl pH 8.8, 5 mM EDTA, 100 mM KCl, 1% (w/v) dithiothreitol (DTT)] containing a complete protease inhibitor cocktail (Roche Applied Science, Roche Diagnostics, Mannheim, Germany) and sonicated (power: 30%, duration: 10 s, cycle (pulsation): 5 × 10%, Bandelin Sonopuls HD2070, Bandelin electronic, Berlin, Germany) three times on ice with a 30 s rest period in between. Consequently, a centrifugation step was performed at 18,000 × g for 15 min at 4°C. The pellets containing rice starch particles were discarded. The supernatants were then mixed with lysis buffer (pH: 8.5) containing 7 M urea, 2 M thiourea, 4% (w/v) CHAPS, 30 mM Tris-HCl (Arnal et al., 2015) to which complete protease inhibitor cocktail was added. Protein concentration was measured using the 2-D Quant kit (GE Healthcare Life Sciences, Sigma-Aldrich Handels GmbH, Vienna, Austria) based on the manufacturer's instructions. The 2-D Clean up kit (GE Healthcare Life Sciences) was used for further protein purification. The resulting protein pellet was resuspended in the lysis buffer described above and stored at −80°C.

**Table 1:** Experimental design for the 2D-DIGE-α and 2D-DIGE-β experiments.

**Gel Nr**.	**Fluorescent dyes**		
	**G-Dye100**	**G-Dye200**	**G-Dye300**
1	IS (50 μg)	25a (50 μg)	303b (50 μg)
2	IS (50 μg)	25b (50 μg)	303a (50 μg)
3	IS (50 μg)	303b (50 μg)	25a (50 μg)
4	IS (50 μg)	303a (50 μg)	25b (50 μg)

*IS, Internal standard*.

*25a, Biological replicate 1 of virulent Histomonas meleagridis parasites, of passage number 25, protein extraction on day 1*.

*25b, Biological replicate 2 of virulent H. meleagridis parasites, of passage number 25, protein extraction on day 2*.

*303a, Biological replicate 1 of attenuated H. meleagridis parasites, of passage number 303, protein extraction on day 1*.

*303b, Biological replicate 2 of attenuated H. meleagridis parasites, of passage number 303, protein extraction on day 2*.

The first group of four protein samples was used for the shotgun proteomic analysis and in a 2D-DIGE experiment, designated as 2D-DIGE-α. The second group was used in a repetition of the 2D-DIGE experiment, referred to as 2D-DIGE-β.

### Protein labeling with G-Dye fluorophores and 2D-DIGE protocol

The protein concentration of all four protein samples was measured with the 2-D Quant kit to ensure accurate sample application and labeling. The G-Dye100 (G100), G-Dye200 (G200), and G-Dye300 (G300) lysine fluorescent dyes, included in the Refraction-2D™ kit (NH DyeGNOSTICS GmbH, Halle, Germany) were reconstituted and used for the minimal labeling of protein samples according to manufacturer's instructions. Each 2D-DIGE experiment consisted of four 2-DE gels. In each gel, three samples were visualized, namely 25a or 25b, 303a or 303b and the IS (Table 1). The IS sample, labeled with G-Dye100, contained an equal amount of all four protein samples. To exclude preferential labeling bias, the four protein samples were individually labeled with 400 pmol of G-Dye200 or G-Dye300 dye using dye-swap correction (Table 1). In each gel, 150 μg of protein was resolved in the first dimension by conducting isoelectric focusing (IEF) and in the second dimension by SDS-PAGE.

Previous to IEF, 18 cm IPG strips with a linear pH range 4–7 (ReadyStrip™ IPG strips, Bio-Rad®, Vienna, Austria) were rehydrated for 16–17 h at room temperature in rehydration buffer [7 M urea, 2 M thiourea, 4% (w/v) CHAPS, 0.002% (w/v) bromophenol blue] to which 0.4% (w/v) DTT and 0.5% (v/v) SERVALYT™ 3–10 Carrier Ampholytes (Serva Electrophoresis, Heidelberg, Germany) were added (Arnal et al., 2015). The sample application was performed according to the anodic cup-loading technique. In the first dimension, the proteins were focused using PROTEAN® IEF Cell (Bio-Rad®) at 20°C with an electric current limitation of 50 μA/IPG strip. Isoelectric focusing was performed in six steps with the following voltage settings and duration: (i) 150 V for 1.5 h, (ii) 300 V for 1.5 h, (iii) 600 V for 1 h, (iv) linear voltage increase to 8,000 V for 0.5 h, (v) final focusing step at 8,000 V for a total of 45,000 Vh, (vi) 500 V resting step. Following this, all four IPG strips that belonged to the same 2D-DIGE experiment were stored at −80°C.

Prior to the separation of focused proteins by SDS-PAGE, the reduction of the thawed IPG strips was performed in equilibration buffer (6 M urea, 0.375 M Tris-HCl pH 8.8, 2% SDS, 20% glycerol) containing 2% (w/v) DTT for 15 min at room temperature. Then the IPG strips were alkylated under the same conditions and in the same equilibration buffer which contained 2.5% (w/v) iodoacetamide (IAA) instead of DTT.

For the second dimension, vertical slab gels [resolving gel dimensions: width × height × thickness = 18.3 cm × 16.3 cm × 1.5 mm, 1.5 M Tris-HCl pH 8.8, 10% SDS, (total monomer concentration) T = 10%, (percentage of crosslinker) C = 3.33%] were casted simultaneously using the PROTEAN® II multi-gel casting chamber (Bio-Rad®) according to manufacturer's instructions. Subsequently, the equilibrated IPG strips were placed on top of a polymerized stacking gel (18.3 cm × 3 cm × 1.5 mm) using 0.5% pre-warmed agarose, containing 0.05% of bromophenol blue dye, which was used for tracking purposes. The second dimension was carried out using Protean® II xi cell (Bio-Rad®) with a current of 15 mA/gel at 4°C until the tracking dye ran off the bottom of the gels. Fifteen microliters of molecular marker [Spectra^TM^ Multicolor Broad Range Protein Ladder, 10–260 kDa, Thermo Scientific™, Fisher Scientific (Austria) GmbH, Vienna, Austria] was applied for approximate estimation of protein molecular mass (*M*_r_).

The fluorescent labeled gels were scanned with Typhoon 9400 scanner (GE Healthcare Life Sciences) at a resolution of 100 μm. After scanning, protein patterns were additionally visualized following published silver staining protocols (Blum et al., 1989; Miller and Gemeiner, 1992).

### Computational image and statistical analysis of fluorescent proteome patterns

Twelve fluorescent gel images were obtained from each 2D-DIGE experiment and included in the computational image (Figure S2) and statistical analysis which was performed with the Delta2D software version 4.7 (Decodon GmbH, Greifswald, Germany) (Berth et al., 2007). The investigations were characterized as multiplex experiments. The 12 fluorescent images were categorized into three groups consisting of four images each. The three groups were termed “virulent 25,” “attenuated 303,” and “IS.” The categorization was performed by assigning gel, sample and channel information to each image. The “in gel standard” warping strategy was chosen to coordinate corresponding spot regions across all gel images and to eliminate positional deviations due to technical reasons. The “union fusion” algorithm was applied to combine all gel images in a single fusion image based on their common region. IS images were excluded from this step as they do not add information to the consensus protein spot pattern of the parasites. Spot detection was performed once on the fusion image. The consensus spot pattern information was transferred to all gel images, including the IS images, allowing for the acquisition of complete expression profiles for a given spot across all 12 fluorescent gel images.

The statistical analysis of expression profiles was carried out with TIGR Multiple Experiment Viewer (MeV) which is included in Delta2D software version 4.7 (Decodon GmbH). The IS images were not included in the statistical analysis but were taken into account for the normalization of protein spots visualized on gel images belonging to the virulent (passage number 25) and attenuated (passage number 303) group. In all four gels of a given 2D-DIGE experiment, the same sample served as the IS; therefore, all spots were reliably normalized to the same sample. Hypothesis testing was performed using a permutation Student's *t-*Test by assuming unequal group variances and by applying the following false discovery rate (FDR) correction in order to address the multiple hypothesis testing problem (Benjamini and Hochberg, 1995): with a confidence level of 95% the number of accepted false positive expression profiles was ≤20. Protein spots with significant (*P* < 0.05) differences in the mean normalized volumes (%V), between virulent and attenuated gel image groups, were submitted for identification by MALDI-TOF/TOF or LC-MS/MS.

### Identification of differentially abundant protein spots by MALDI-TOF/TOF

Significantly (*P* < 0.05) differentially abundant protein spots that could be located on silver-stained fluorescent gels were excised and processed for identification. Protein spots were pooled from two or more gels to obtain sufficient material for mass spectrometric analysis. After washing and de-staining, reduction of spots with DTT and alkylation with IAA was conducted (Gharahdaghi et al., 1999; Jiménez et al., 2001). Consequently, in-gel digestion with trypsin (Trypsin Gold, Mass Spectrometry Grade, Promega, Madison, USA) was performed according to Shevchenko et al. (1996). The peptides were then extracted and dried using a vacuum concentrator (Eppendorf, Hamburg, Germany). Afterwards, de-salting and concentrating of tryptic peptides was performed to increase the sensitivity of MS using μ-Zip Tips C18 (Millipore, Billerica, USA) according to the manufacturer's instructions.

A 0.5 μl aliquot of de-salted peptides was added to 0.5 μl of the matrix solution [α-cyano-4-hydroxycinnamic acid, saturated in acetonitrile–0.1% trifluoroacetic acid (30:70, v/v)] and 1 μl of the resulting mixture was spotted on a ground steel MALDI target plate (Bruker Daltonics, Bremen, Germany) and allowed to dry at room temperature. Spectra data were acquired in MS and MS/MS modes by performing MALDI-TOF/TOF MS (Ultraflex II, Bruker Daltonics). FlexAnalysis 3.0 and Biotools 3.2 software (Bruker Daltonics) were used for spectra processing and peak annotation. Processed spectra were submitted to an in-house Mascot server (version 2.3, Matrix Science, Boston, USA) using the ProteinScape 2.1 software (Bruker Daltonics). Subsequent searches were performed in an amino acid database of *E. coli* proteins (UnitProt DB, taxonomy 562 EcolX^1^) combined with a database containing conceptually translated contigs from the *H. meleagridis* reference transcriptome (size: 3356 unique contigs; study accession number PRJEB19109^2^) (Mazumdar et al., 2017) and common Repository of Adventitious Proteins (cRAP) database^3^. The *H. meleagridis* contigs were annotated based on their homology to known genes (Mazumdar et al., 2017). The parameters for the search were: fixed modifications = carbamidomethylation (C); variable modifications = oxidation (M), deamidation (NQ), pyroglutamic acid formation; enzyme specificity = trypsin; charge state (Z) = +1; maximum missed cleavages allowed = 2; peptide mass tolerance = 100 ppm (150 ppm in some cases); fragment mass tolerance = 1.0 Da. Mascot identifications having at least one peptide with a Mascot score >20 were regarded as statistical significant (*P* < 0.05), while in ProteinScape 2.1 software, protein scores >80 were considered as significant.

### Identification of differentially abundant protein spots by LC-MS/MS

Five and three protein spots from the 2D-DIGE-α and 2D-DIGE-β experiment, respectively, could not be identified by MALDI-TOF/TOF MS and were analyzed by LC-MS/MS. For this, proteolytically generated peptides were separated by a high-performance chromatography system (nano-HPLC Ultimate 3000 RSLC system, Dionex) and analyzed with a high-resolution hybrid triple quadrupole time-of-flight mass spectrometer (TripleTOF 5600+, Sciex, USA) connected via nano-electrospray ionization (ESI) interface.

Remainders of the de-salted peptides after in-gel digest were evaporated and reconstituted in 0.1% aqueous TFA for injection into LC-MS. Sample pre-concentration and de-salting was accomplished with a 5 mm Acclaim PepMap μ-Precolumn (300 μm inner diameter, 5 μm particle size, and 100 Å pore size) (Dionex). As a mobile phase 2% ACN in ultra-pure H_2_O with 0.05% TFA was used for loading and de-salting of peptide samples with a flow rate of 5 μl/min. For peptide separation a 25 cm Acclaim PepMap C18 column (75 μm inner diameter, 3 μm particle size, and 100 Å pore size) was used operated at a flow rate of 300 nl/min. The gradient started with 4% B (80% ACN with 0.1% formic acid) and increased to 35% B in 60 min. It was followed by a washing step with 90% B. Mobile Phase A consisted of ultra-pure H_2_O with 0.1% formic acid. Mass spectra of HPLC-separated peptides were acquired using an information dependent data acquisition mode (IDA). MS1 spectra were collected in the range of 400–1,500 m/z for 250 ms. The 25 most intense precursors with charge state 2–4, which exceeded 100 counts per second, were selected for fragmentation. MS2 spectra were acquired in the range of 100–1,800 m/z for 110 ms. Precursor ions were dynamically excluded from reselection for 10 s. The nano-HPLC system was operated by Chromeleon 6.8 using DCMS-Link (Dionex) and the MS by Analyst Software 1.6 (Sciex).

Database searches of raw data were performed using ProteinPilot 5.0 (Sciex) in the combined database of *E. coli* and *H. meleagridis* as described above in: Identification of differentially abundant protein spots by MALDI-TOF/TOF. Mass tolerance in MS mode was set with 0.05 and 0.1 Da in MSMS mode for the rapid recalibration search, and 0.0011 Da in MS and 0.01 Da in MSMS mode for the final search. Parameters for database search: trypsin digestion, cysteine alkylation set to iodoacetamide, search effort set to rapid ID. FDR was performed using the integrated tools in ProteinPilot. Global FDR was set to < 1% on protein as well as on peptide level. Following the generally applied so-called “two-peptide rule” only proteins with at least two peptides were further considered as identified (Taylor and Goodlett, 2005).

### Shotgun protein identification using LC-MS/MS

For identification of the *H. meleagridis* proteins by LC-MS/MS without previous gel electrophoretic separation, protein digestion was performed using a two-step in-solution digestion protocol with a combination of LysC and trypsin according to the manufacturer's recommendation^4^). For each sample 10 μg of total protein were diluted with 8 M urea in 50 mM Tris pH 8.0, to a total volume of 10 μl. Proteins were reduced with 50 mM DTT (30 min, 37°C) and alkylated with 200 mM IAA (30 min, 25°C) before stepwise digestion with Trypsin/LysC mix (Promega) (4 h, 37°C; dilution with 50 mM Tris; 8 h, 37°C). After acidification with concentrated TFA, samples were frozen at −20°C until mass spectrometric analysis. An aliquot of peptides was analyzed by LC-MS/MS as described above in the section Identification of Differentially Abundant Protein Spots by LC-MS/MS.

### Label-free quantification and statistical analysis with SWATH MS technology—creation of SWATH ion library

LC parameters for data independent SWATH quantification analyses were the same as described above for IDA injections. MS parameters were adapted for SWATH. MS1 spectra were collected in the range of 400–1,500 m/z with an accumulation time of 50 ms. Product ion spectra were collected in 34 windows in the range of 400–1,250 m/z with a fixed width of 25 Da. For each window ions were accumulated for 80 ms.

IDA identification results (see section Shotgun Protein Identification Using LC-MS/MS) were used to create the SWATH ion library with SWATH Acquisition MicroApp 2.0 in PeakView 2.2 (both Sciex, USA). Peptides were chosen based on a FDR rate < 1%, excluding shared and modified peptides. Up to 6 peptides per protein and up to 6 transitions per peptide were used. Calculation of peak areas of SWATH samples was performed with MarkerView 1.2.1 (Sciex, USA) after retention time alignment and normalization using total area sums.

Further statistical evaluations were based on the normalized peak areas. Two different software tools were used: R programming language (R Development Core Team, 2014) as well as MarkerView (Sciex).

### Label-free quantification and statistical analysis with SWATH MS technology—markerview

After principal component analysis (PCA), groups were defined and a *t-*Test was performed. Proteins with at least two peptides were considered differential if the *p*-value was below α = 0.00001 and the absolute fold change was at least two (fold change < −2 or >+2). As MarkerView does not differentiate between technical and biological replicates an additional statistical evaluation with R was performed.

### Label-free quantification and statistical analysis with SWATH MS technology—r programming language

cRAP proteins and proteins quantified with just one peptide were removed from the MarkerView normalized raw protein list before further processing. Raw peak areas were log2-transformed to approach a normal distribution. On a logarithmic scale, technical replicates were aggregated by arithmetic mean before application of statistical tests.

Differential expression of proteins was assessed using two-tailed *t-*Test for independent samples for each protein. To adjust for multiple testing, the method of Benjamini and Hochberg was used to control the FDR (Benjamini and Hochberg, 1995). Protein expression was considered differential if the adjusted *p*-value was below α = 0.08 and the absolute fold change was at least two (fold change < −2 or >+2).

### Protein-function network graphs constructed for *H. meleagridis*-specific proteins

The open source software platform Cytoscape version 3.5.1 (Shannon et al., 2003) was used to display the link between identified *H. meleagridis*-specific proteins and their proposed function. The identified proteins were found to be significantly differentially expressed between the two strains by the 2D-DIGE-α, 2D-DIGE-β, and SWATH MS experiments. The proposed functions were attributed to identified proteins based on their Gene Ontology terms, InterPro entries and existing literature. The fold upregulation values, which were acquired from Delta2D software version 4.7 (Decodon GmbH) and SWATH MS quantification measurements, were mapped to certain colors. In this way, the *H. meleagridis*-specific proteins, which were represented by source nodes, were visually characterized and their level of expression was indicated.

## Results

### Fluorescent protein patterns of the *in vitro* cultivated virulent and attenuated *H. meleagridis* parasites

Protein spots were distributed within a pH range of 5–7 and a *M*_r_ of 15–140 kDa (Figures S3, S4). The landmark spot, which was distinctive at pI 5 and a *M*_r_ of ~50 kDa (Figures S3, S4), was known to be actin of *H. meleagridis* origin based upon a previous study (Monoyios et al., 2018). The visualization of this actin spot with yellow color was an indication that it was not differentially expressed between the *in vitro* cultivated virulent and attenuated parasites (Figure S3). This observation was later on confirmed by the statistical analysis of the fluorescent proteome patterns. The projection of virulent and attenuated protein spots with pseudo- colors revealed that high *M*_r_ proteins (50–140 kDa) were more abundant in the attenuated strain (Figures S3, S4). In contrast to this, proteins of low *M*_r_ (15–50 kDa) were more numerous in the protein samples of the virulent strain (Figures S3, S4).

### Detection of significantly differentially abundant protein spots by 2D-DIGE

Initially, 937 and 1297 protein spots were detected on the fusion images, which were generated by the 2D-DIGE-α and 2D-DIGE-β computational image analysis, respectively. Artifacts and faint spots were removed by setting a spots' quality threshold and by performing manual spot editing. As a result, 563 and 762 protein spots were included in the statistical evaluation of fluorescent proteome patterns obtained by the 2D-DIGE-α and 2D-DIGE-β experiment, respectively.

**Figure 1:**
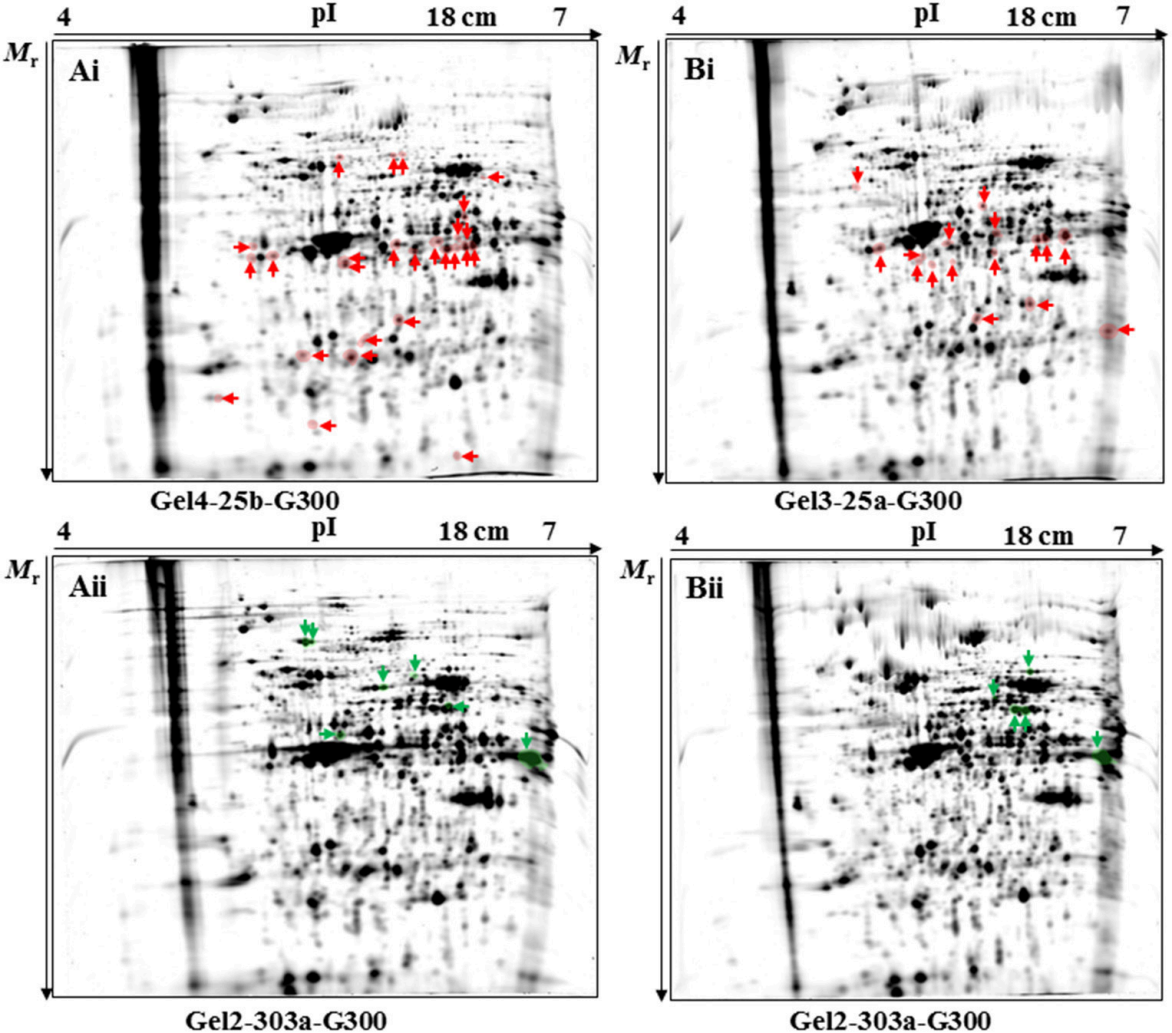
Position of protein spots detected as significantly (*P* < 0.05) differentially expressed by the 2D-DIGE-α **(A)** and 2D-DIGE-β **(B)** experiment. Protein spots found to be significantly upregulated in the fluorescent images of the cultivated virulent strain (passage number 25) by the 2D-DIGE-α **(Ai)** and 2D-DIGE-β **(Bi)** experiment are highlighted with red color and are indicated by red arrows. Fluorescent gel images with codenames Gel4-25b-G300 **(Ai)** and Gel3-25a-G300 **(Bi)** were chosen as the representative gel images for spot visualization. Protein spots found to be significantly upregulated in the fluorescent images of the cultivated attenuated strain (passage number 303) by the 2D-DIGE-α **(Aii)** and 2D-DIGE-β **(Bii)** experiment are highlighted with green color and are indicated by green arrows. Fluorescent gels images with the codenames Gel2-303a-G300 **(Aii)** and Gel2-303a-G300 **(Bii)** were chosen as the representative gel images for spot visualization. The position of identified protein spots is additionally displayed on the corresponding silver-stained fluorescent gels (Figures S6, S7). The arrows at the x-and y- axis in each gel are indicating the direction of protein mobility according to their pI (x-axis) and *M*_r_ (y-axis). 2D-DIGE, two-dimensional differential gel electrophoresis; 25, virulent *H. meleagridis* parasites harvested at passage number 25; 303, attenuated *H. meleagridis* parasites harvested at passage number 303; 25a and 25b, protein samples extracted from virulent *H. meleagridis* parasites on day 1 and day 2, respectively; 303a and 303b, protein samples extracted from attenuated *H. meleagridis* parasites on day 1 and day 2, respectively; G300, protein samples labeled with G-Dye 300; *M*_r_, molecular mass; pI, isolelectric point.

In the 2D-DIGE-α experiment, the statistical analysis detected a total number of 33 protein spots as differentially expressed (Figures 1Ai,Aii and Figure S5A). The fold change in their expression levels ranged between 1.234 and 3.458 (Table 2). Twenty-six out of the 33 protein spots were significantly overexpressed in gel images containing proteins of the virulent culture (Figure 1Ai). However, three out of these could not be located on silver-stained fluorescent gels obtained by the 2D-DIGE-α experiment (Figure S6A). The seven remaining protein spots, out of the total 33, were found overexpressed in the gel images that contained proteins of the attenuated culture (Figure 1Aii). All seven spots were located at a *M*_r_ of 50 kDa and above (Figure 1Aii, Figure S6B). In contrast, only four out of the 26 protein spots, with overexpression in the gel images of the virulent culture, were located above 50 kDa (Figure 1Ai, Figure S6A).

**Table 2:** Summary of mass spectrometric results and corresponding identifications of protein spots with significant (*P* < 0.05) upregulation in fluorescent gel images of the virulent (25) and attenuated (303) *Histomonas meleagridis* strain.

**Spot ID^a^**	**Protein identity- Species- Contig ID^b^; Accession Nr**.	**Identification method**	**Gel image^c^**	**Theor. pI/ *M*${}_{\text{r}}^{\text{d}}$**	**Fold upregulation^e^**	**Protein score^f^**	**Nr. of peptides**
**CYTOSKELETON/PLASMINOGEN (PLG)-BINDING: VIRULENT** ***H. meleagridis***							
63527	Actin- Contig2112; HAGI01002078	MALDI-TOF/TOF	25	5.1/42.5	2.554	246	2
78829	Actin- Contig2112; HAGI01002078	MALDI-TOF/TOF	25	5.1/42.5	2.088	279	3
63624	Actin- Contig2112; HAGI01002078	MALDI-TOF/TOF	25	5.1/42.5	1.916	301	4
63646	Actin- Contig2112; HAGI01002078	MALDI-TOF/TOF	25	5.1/42.5	1.871	200	3
63512	Actin- Contig2112; HAGI01002078	MALDI-TOF/TOF	25	5.1/42.5	1.613	243	3
**CARBOHYDRATE METABOLISM: VIRULENT** ***H. meleagridis***							
63324	Pyruvate phosphate dikinase (PPDK)- Contig1102; HAGI01001086	LC-MS/MS	25	5.7/55.7	2.255	43	4
79153	Phosphoenolpyruvate carboxykinase (PEPCK)- Contig1899; HAGI01001872	MALDI-TOF/TOF	25	5.8/67.1	1.881	163	2
63521	Phosphoenolpyruvate carboxykinase (PEPCK)- Contig1899; HAGI01001872	MALDI-TOF/TOF	25	5.8/67.1	1.614	351	5
63471	Phosphoenolpyruvate carboxykinase (PEPCK)- Contig1899; HAGI01001872	MALDI-TOF/TOF	25	5.8/67.1	1.473	330	5
101054	Phosphoenolpyruvate carboxykinase (PEPCK)- Contig1899; HAGI01001872	MALDI-TOF/TOF	25	5.8/67.1	1.391	107	2
**CARBOHYDRATE METABOLISM/PLASMINOGEN (PLG)-BINDING: VIRULENT** ***H. meleagridis***							
63536	Glyceraldehyde-3-phosphate dehydrogenase (GAPDH)- Contig449; HAGI01000443	MALDI-TOF/TOF	25	6.6/40.0	3.458	107	2
63529	Glyceraldehyde-3-phosphate dehydrogenase (GAPDH)- Contig449; HAGI01000443	MALDI-TOF/TOF	25	6.6/40.0	2.424	346	4
63528	Glyceraldehyde-3-phosphate dehydrogenase (GAPDH)- Contig449; HAGI01000443	MALDI-TOF/TOF	25	6.6/40.0	2.401	269	3
63530	Glyceraldehyde-3-phosphate dehydrogenase (GAPDH)- Contig449; HAGI01000443	MALDI-TOF/TOF	25	6.6/40.0	1.766	304	3
**ADAPTATION TO STRESS: VIRULENT** ***H. meleagridis***							
219386	14-3-3 protein- Contig1890; HAGI01001863	LC-MS/MS	25	4.7/27.5	2.469	304	23
84570	Cytosolic heat shock protein 70 (Cytosolic Hsp70)- Contig764; HAGI01000755	MALDI-TOF/TOF	25	4.9/48.0	2.137	276	3
**PEPTIDASE ACTIVITY: VIRULENT** ***H. meleagridis***							
63659	Clan CD, family C13, asparaginyl endopeptidase-like cysteine peptidase (Peptidase C13)- Contig42; HAGI01000042	MALDI-TOF/TOF	25	5.9/44.5	1.910	283	3
**INTRACELLULAR VESICLE TRAFFICKING: VIRULENT** ***H. meleagridis***							
63717	Rab family GTPase (Rab11c-like)- Contig1980; HAGI01001951	LC-MS/MS	25	5.97/22.9	1.705	113	8
**UNKNOWN FUNCTION: VIRULENT** ***H. meleagridis***							
63318	Hypothetical protein- Contig1489; HAGI01001468	LC-MS/MS	25	5.0/38.3	1.525	168	12
**METABOLIC PROCESSES: VIRULENT** ***H. meleagridis***							
63664	Phosphomanomutase (PMM)- Contig1867; HAGI01001842	MALDI-TOF/TOF	25	5.8/28.4	1.234	243	4
**ADAPTATION TO STRESS: BACTERIAL**							
63323	Chaperone protein ClpB- *Escherichia coli*- J7R7G1_ECOLX	LC-MS/MS	25	5.4/95.5	2.105	256	19
**METABOLIC PROCESSES: BACTERIAL**							
105883	Glycerophosphodiester phosphodiesterase- *E. coli*- A0A024L1F7_ECOLX	MALDI-TOF/TOF	25	5.3/40.8	1.615	236	3
**CARBOHYDRATE UP-TAKE/ADAPTATION TO STRESS: BACTERIAL**							
106853	Maltose ABC transporter substrate-binding protein MalE- *E. coli*- A0A023LBH2_ECOLX	MALDI-TOF/TOF	25	5.4/43.4	1.521	104	2
**CELL DIVISION: ATTENUATED** ***H. meleagridis***							
63266	Cell division cycle protein 48-like (Cdc48-like)- Contig1011; HAGI01000996	MALDI-TOF/TOF	303	4.9/88.0	1.972	801	8
63274	Cell division cycle protein 48-like (Cdc48-like)- Contig1011; HAGI01000996	MALDI-TOF/TOF	303	4.9/88.0	1.908	440	6
**CARBOHYDRATE METABOLISM/PLASMINOGEN (PLG)-BINDING: ATTENUATED** ***H. meleagridis***							
63468	Enolase family protein- Contig1151; HAGI01001135	MALDI-TOF/TOF	303	5.2/51.3	1.638	447	5
63509	Glyceraldehyde-3-phosphate dehydrogenase (GAPDH)- Contig449; HAGI01000443	MALDI-TOF/TOF	303	6.6/40.0	1.438	575	5
**OTHER**							
63347	Serum albumin precursor- *Bos taurus*- ALBU_BOVIN	MALDI-TOF/TOF	303	5.8/69.2	2.828	274	5
**METABOLIC PROCESSES: ATTENUATED** ***H. meleagridis***							
63369	Clan MH, family M20, peptidase T-like metallopeptidase- Contig1068; HAGI01001053	MALDI-TOF/TOF	303	5.3/62.9	1.727	425	5
**CYTOSKELETON ORGANIZATION: ATTENUATED** ***H. meleagridis***							
63416	Coronin- Contig2106; HAGI01002072	MALDI-TOF/TOF	303	5.9/50.2	1.681	199	3

*Results were obtained by the two-dimensional differential gel electrophoresis (2D-DIGE) experiment, designated as 2D-DIGE-α. The identified proteins were categorized according to their proposed function and listed within categories according to their fold upregulation (from high to low)*.

*Spots of interest were excised and pooled from all four silver-stained fluorescent gels included in the 2D-DIGE-α experiment*.

*Proteins that take part in metabolic processes other than carbohydrate metabolism were grouped together*.

a *Spot ID = a unique number assigned to each protein spot by Delta2D software version 4.7 (Decodon GmbH, Greifswald, Germany)*.

b *Contig identification number (ID) was obtained from the de novo transcriptome sequencing of a virulent and an attenuated H. meleagridis strain (Mazumdar et al., 2017)*.

c *Gel image = 25: the protein spot was significantly upregulated in fluorescent gel images displaying proteins of the cultivated virulent strain*.

303: the protein spot was significantly upregulated in fluorescent gel images displaying proteins of the cultivated attenuated strain.

d *The theoretical isoelectric point (pI) and molecular mass (M_r_) of each protein*.

e *The fold upregulation of each protein spot which was assigned by Delta2D software version 4.7 (Decodon GmbH)*.

f *Protein score = MALDI-TOF/TOF identifications with protein score >80 are significant in MASCOT and fulfill the stricter criteria of ProteinScape 2.1 software*. *The following protein spots were not identified: 63535 (gel image: 25, fold upregulation: 1.661), 63735 (gel image: 25, fold upregulation: 1.932), 71550 (gel image: 25, fold upregulation: 2.072)*.

In the 2D-DIGE-β experiment the statistical analysis detected a total number of 21 protein spots as differentially expressed (Figures 1Bi,Bii and Figure S5B). The fold change in their expression levels ranged between 1.255 and 3.443 (Table 3). Sixteen out of the 21 differentially expressed protein spots were detected in gel images containing proteins of the virulent culture (Figure 1Bi). Identifying two out of these spots from silver-stained fluorescent gels was not possible (Figure S7A). In agreement with the 2D-DIGE-α experiment, only two out of the 16 protein spots were located above 50 kDa (Figure 1Bi, Figure S7A). The five remaining spots, out of the total 21, were detected in the gel images of the attenuated culture (Figure 1Bii). As it was observed in the 2D-DIGE-α experiment for this strain, all five spots were located at a *M*_r_ of ≥50 kDa (Figure 1Bii, Figure S7B).

**Table 3:** Summary of mass spectrometric results and corresponding identifications of protein spots with significant (*P* < 0.05) upregulation in fluorescent gel images of the virulent (25) and attenuated (303) *Histomonas meleagridis* strain.

**Spot ID^a^**	**Protein identity- Species-Contig ID^b^; Accession Nr**.	**Identification method**	**Gel image^c^**	**Theor. pI/ *M*${}_{\text{r}}^{\text{d}}$**	**Fold upregulation^e^**	**Protein score^F^**	**Nr. of peptides**
**CARBOHYDRATE METABOLISM/ PLASMINOGEN (PLG)-BINDING: VIRULENT** ***H. meleagridis***							
610	Fructose-bisphosphate aldolase (FBAL)- Contig601; HAGI01000595	MALDI-TOF/TOF	25	6.0/37.2	2.726	385	6
526	Glyceraldehyde-3-phosphate dehydrogenase (GAPDH)- Contig449; HAGI01000443	MALDI-TOF/TOF	25	6.6/40.0	2.708	371	4
509	Glyceraldehyde-3-phosphate dehydrogenase (GAPDH)- Contig449; HAGI01000443	MALDI-TOF/TOF	25	6.6/40.0	1.856	445	4
524	Glyceraldehyde-3-phosphate dehydrogenase (GAPDH)- Contig449; HAGI01000443	MALDI-TOF/TOF	25	6.6/40.0	1.811	318	4
**CYTOSKELETON/PLASMINOGEN (PLG)-BINDING: VIRULENT** ***H. meleagridis***							
535	Actin- Contig2112; HAGI01002078	MALDI-TOF/TOF	25	5.1/42.5	2.942	124	2
627	Actin- Contig2112; HAGI01002078	MALDI-TOF/TOF	25	5.1/42.5	2.725	169	2
386163	Actin family protein- Contig444; HAGI01000438	MALDI-TOF/TOF	25	6.5/44.6	2.687	128	2
**CARBOHYDRATE METABOLISM: VIRULENT** ***H. meleagridis***							
202345	Phosphoenolpyruvate carboxykinase (PEPCK)- Contig1899; HAGI01001872	MALDI-TOF/TOF	25	5.8/67.1	2.400	415	5
63267	Iron-containing alcohol dehydrogenase (ADH)- Contig362; HAGI01000356	LC-MS/MS	25	5.6/43.5	2.259	246	17
640	Phosphoglycerate mutase (PGlyM)- Contig1739; HAGI01001716	MALDI-TOF/TOF	25	6.8/29.8	1.602	294	4
**METABOLIC PROCESSES: VIRULENT** ***H. meleagridis***							
42635	Clan MG, family M24, aminopeptidase P-like metallopeptidase- Contig29; HAGI01000029	MALDI-TOF/TOF	25	5.2/51.9	2.510	101	1
356639	Clan MG, family M24, aminopeptidase P-like metallopeptidase- Contig29; HAGI01000029	LC-MS/MS	25	5.2/51.9	1.345	102	8
**ADAPTATION TO STRESS: BACTERIAL**							
417	60 kDa chaperonin- *Escherichia coli*- Q6Q099_ECOLX	LC-MS/MS	25	4.8/57.3	1.535	305	21
**CARBOHYDRATE METABOLISM: BACTERIAL**							
197187	Class II fructose-bisphosphate aldolase- *E. coli*- A0A0D8VY27_ECOLX	MALDI-TOF/TOF	25	5.5/39.1	1.404	220	2
**CYTOSKELETON ORGANIZATION: ATTENUATED** ***H. meleagridis***							
408	Coronin- Contig2106; HAGI01002072	MALDI-TOF/TOF	303	5.9/50.2	1.492	525	5
409	Coronin- Contig1193; HAGI01001177	MALDI-TOF/TOF	303	5.9/50.3	1.395	716	7
**METABOLIC PROCESSES: ATTENUATED** ***H. meleagridis***							
587944-1	Transketolase family protein- Contig1077; HAGI01001062	MALDI-TOF/TOF	303	5.4/62.7	1.551	231	3
587944-2	Transketolase family protein- Contig1077; HAGI01001062	MALDI-TOF/TOF	303-Gel4	5.4/62.7	1.551	246	4
**CARBOHYDRATE METABOLISM/PLASMINOGEN (PLG)-BINDING: ATTENUATED** ***H. meleagridis***							
495	Glyceraldehyde-3-phosphate dehydrogenase (GAPDH)- Contig449; HAGI01000443	MALDI-TOF/TOF	303	6.6/40.0	1.460	455	4
**METABOLIC PROCESSES: BACTERIAL**							
153815	Bifunctional purine biosynthesis protein purH- *E. coli*- A0A085PAJ0_ECOLX	MALDI-TOF/TOF	303	5.5/57.3	1.414	502	6

*Results were obtained by the two-dimensional differential gel electrophoresis (2D-DIGE) experiment, designated as 2D-DIGE-β. The identified proteins were categorized according to their proposed function and listed within categories according to their fold upregulation (from high to low)*.

*Spots of interest were excised and pooled from all four silver-stained fluorescent gels included in the 2D-DIGE-β experiment*.

*Proteins that take part in metabolic processes other than carbohydrate metabolism are grouped together*.

a *Spot ID = a unique number assigned to each protein spot by Delta2D software version 4.7 (Decodon GmbH, Greifswald, Germany)*.

b *Contig identification number (ID) was obtained from the de novo transcriptome sequencing of a virulent and an attenuated H. meleagridis strain (Mazumdar et al., 2017)*.

c *Gel image = 25: the protein spot was significantly upregulated in fluorescent gel images displaying proteins of the cultivated virulent strain*.

*303: the protein spot was significantly upregulated in fluorescent gel images displaying proteins of the cultivated attenuated strain*.

*587944-2: The spot was excised and analyzed separately from Gel 4*.

d *The theoretical isoelectric point (pI) and molecular mass (M_r_) of each protein*.

e *The fold upregulation of each protein spot retrieved from Delta2D software version 4.7 (Decodon GmbH)*.

f *Protein score = MALDI-TOF/TOF identifications with protein score >80 are significant in MASCOT and fulfill the stricter criteria of ProteinScape 2.1 software*.

*The following protein spots were not identified: 457 (gel image: 25, fold upregulation: 1.255) and 63593 (gel image: 25, fold upregulation: 3.443)*.

### Identification of proteins detected as significantly differentially abundant by the 2D-DIGE experiments

**Figure 2:**
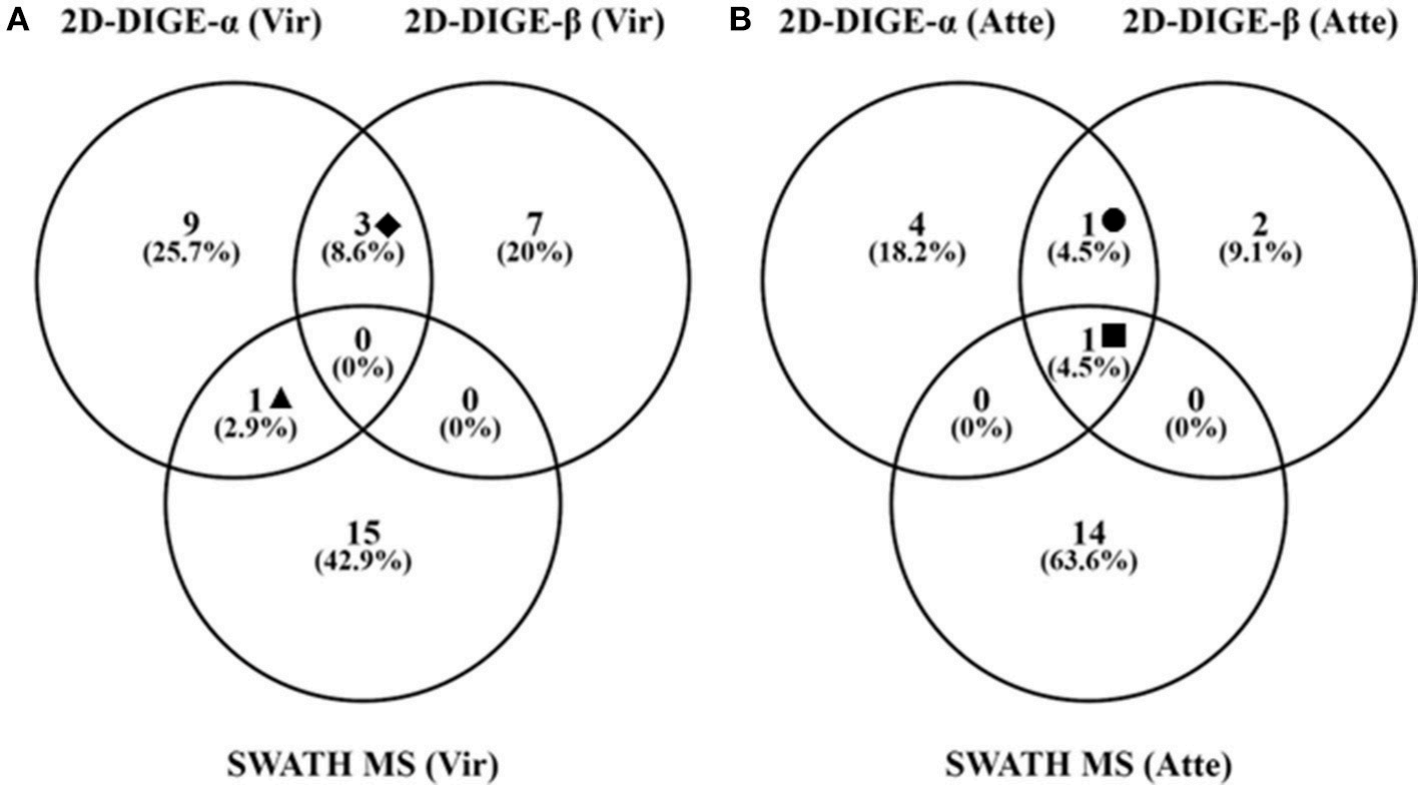
Number of unique and shared identifications for proteins detected as significantly upregulated in the virulent **(A)** and attenuated **(B)** cultivated *H. meleagridis* strain by the 2D-DIGE-α, 2D-DIGE-β, and SWATH MS experiments. Venny 2.1.0 tool (Oliveros, 2015) was used to summarize the findings with venn diagrams. The same extracted protein samples were analyzed by the 2D-DIGE-α and SWATH MS experiment. Based on the analyses the following identified proteins were found to be common between experiments: ♦ = actin, GAPDH and PEPCK **(A)**; ▴ = Clan CD, family C13, asparaginyl endopeptidase-like CP **(A)**; = GAPDH **(B)**; ■ = Coronin **(B)**. 2D-DIGE, two-dimensional differential gel electrophoresis; CP, cysteine peptidase; SWATH MS, sequential window acquisition of all theoretical mass spectra mass spectrometry; GAPDH, glyceraldehyde-3-phosphate dehydrogenase; PEPCK, phosphoenolpyruvate carboxykinase.

Thirty and 19 protein spots, which were detected as significantly (*P* < 0.05) overexpressed by the 2D-DIGE-α and 2D-DIGE-β analysis, respectively, matched with entries from the aforementioned databases (see the section Identification of Differentially Abundant Protein Spots by MALDI-TOF/TOF) (Tables 2, 3 and Tables S1, S2). In the 2D-DIGE-α experiment the 23 spots, which displayed upregulation in the gel images of the virulent culture, were shown to correspond to 13 different proteins (Table 2, Figure 2A), while in the 2D-DIGE-β experiment the 14 protein spots from the same culture were identified as 10 unique proteins (Table 3, Figure 2A). Between the two experiments the common proteins, with overexpression in the virulent strain, were: actin, glyceraldehyde 3-phosphate dehydrogenase (GAPDH) and phosphoenolpyruvate carboxykinase (PEPCK) (Tables 2, 3 and Figure 2A). In the 2D-DIGE-α experiment, the seven upregulated protein spots from the attenuated culture, were identified as six different proteins (Table 2, Figure 2B), while in the 2D-DIGE-β experiment the five protein spots, which exhibited overexpression in the fluorescent gel images of the same culture, represented four unique proteins (Table 3, Figure 2B). In the attenuated culture, the common unique proteins between the two experiments were: coronin and GAPDH (Tables 2, 3 and Figure 2B).

### Identification of protein spots detected as significantly overexpressed in fluorescent gel images of the virulent *H. meleagridis* culture

**Figure 3:**
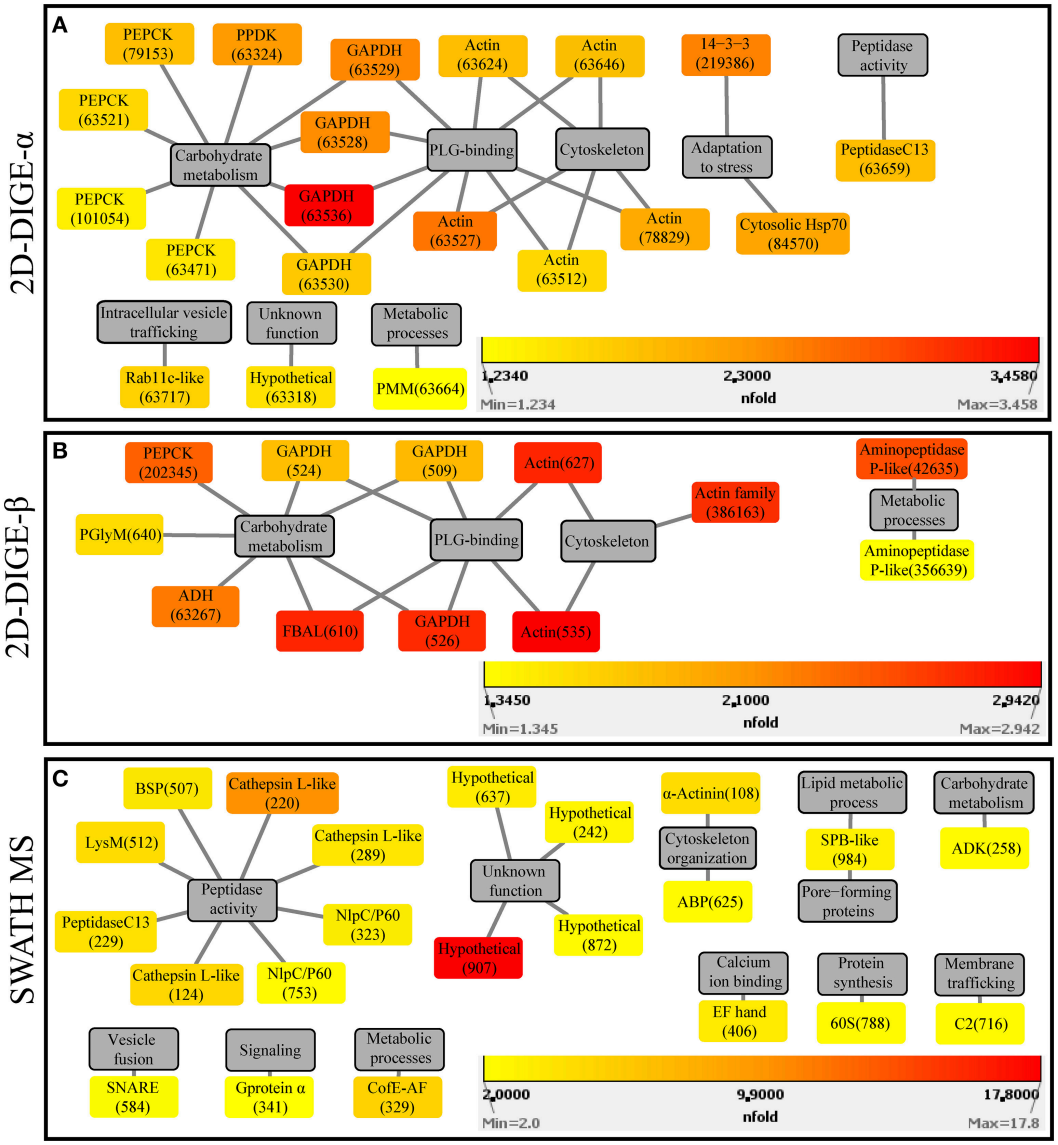
Protein-function network graphs of *Histomonas meleagridis*-specific proteins detected as significantly overexpressed in the virulent strain by the 2D-DIGE-α **(A)**, 2D-DIGE-β **(B)**, and SWATH MS **(C)** experiments. The protein identifications are displayed as color-coded source nodes, with their edges connected to gray colored target nodes which represent the proposed functions. The numbers in parentheses represent the identification numbers assigned to each protein by Delta2D software version 4.7 (Decodon GmbH, Greifswald, Germany) and SWATH MS experiment. In each panel, the dialogs on the right lower corners are the yellow-orange-red gradient which was selected to represent the fold upregulation values of overexpressed proteins from the virulent strain. The source nodes were color-coded based on the fold upregulation data associated with each significantly differentially expressed protein (Tables 2–4). The network layout was manually determined. The reader is referred to Tables 2–4 for explanation of abbreviated protein names. 2D-DIGE, two-dimensional differential gel electrophoresis; SWATH MS, sequential window acquisition of all theoretical mass spectra mass spectrometry.

In the 2D-DIGE-α experiment 20 out of the 23 identified protein spots, with overexpression in the virulent culture, corresponded to 10 *H. meleagridis*-specific proteins (Table 2). Based on their proposed functions these identified proteins can be attributed to eight different categories (Figure 3A). The most frequent identified proteins were actin, followed by GAPDH and PEPCK (Table 2, Figure 3A). As shown by the color-coded source nodes, the protein spots with the highest upregulation were identified as GAPDH (63528, 63529, 63536), actin (63527), and 14-3-3 protein (219386) (Table 2, Figure 3A). Nine identified protein spots corresponded to two unique proteins, namely actin, represented by five spots (63512, 63624, 63527, 63646, 78829) and GAPDH, represented by four spots (63528, 63529, 63530, 63536) which might be associated with alternative functions (Figure 3A). The four GAPDH spots, which were aligned at a *M*_r_ of ~40 kDa, exhibited slight differences in their pI but were all mapped to contig449 (Table 2 and Figures S8Ai,Aii). The five actin spots demonstrated shifts in their pI and were dispersed at the low *M*_r_ area between 25 and 50 kDa (Figures S8Ai,Aii). All actin spots could be mapped to contig2112 (Table 2). The statistical analysis, clustered the actin spots together with a Rab11 isotype (cluster 1), heat shock protein 70 (Hsp70) (cluster 3), legumain-like cysteine peptidase (CP) (cluster 4), and the four GAPDH protein spots (cluster 5) (Figure S5A). The clustering indicates co-regulation of the above proteins. The remaining three spots, found to be overexpressed in the gel images of the virulent culture, represented three proteins of *E. coli* origin that seem to be associated with metabolism and adaptation to stress (Table 2).

In the 2D-DIGE-β group 12 out of the 14 identified protein spots, with upregulation in the gel images of the virulent culture, represented eight unique *H. meleagridis* proteins (Table 3). Based on their proposed functions, the *H. meleagridis*-specific proteins could be connected with four different categories (Figure 3B). The most frequent identified proteins were GAPDH, in three spots out of the total 12 (509, 524, 526), followed by actin, which was represented by two spots (535, 627) (Table 3, Figure 3B). According to the color-coded network, protein spots exhibiting the highest upregulation were identified as actin (535, 627), fructose-bisphosphate aldolase (FBAL) (610), GAPDH (526), and actin family protein (386163) (Table 3, Figure 3B). It should be noted that eight out of the 12 *H. meleagridis*-specific protein spots displayed an upregulation level, which was above the median value (Table 3, Figure 3B). Five out of the eight unique *H. meleagridis* proteins could be connected with carbohydrate metabolism (509, 524, 526, 610, 640, 63267, 202345) (Figure 3B). Six protein spots corresponded to three unique proteins, namely GAPDH (509, 524, 526), actin (535, 627), and FBAL (610) (Figure 3B). Although unrelated, they might be associated with alternative functions. In agreement with the 2D-DIGE-α experiment the three GAPDH protein spots, were also mapped to contig449 and were aligned at a *M*_r_ of ~40 kDa with minor differences in their pI (Table 3 and Figures S8Bi,Bii). The actin spot with identification number 627, displayed a *M*_r_ which was ~15 kDa lower and a pI value which was approximately one unit larger in comparison to the 535 actin spot (Figures S8Bi,Bii). In the statistical analysis, these two actin spots (535, 627) clustered with a GAPDH spot (509), the FBAL spot (610), a Clan MG, family M24, aminopeptidase P-like metallopeptidase (42635), and with other proteins of the carbohydrate metabolism, namely PEPCK (202345) and phosphoglycerate mutase (PGlyM) (640) (cluster 3, Figure S5B). Additionally, another GAPDH spot (524) clustered with an actin family protein (386163) (cluster 1, Figure S5B). The remaining two spots originated from the bacterial background and were connected with stress adaptation and carbohydrate metabolism (Table 3).

### Identification of protein spots detected as significantly upregulated in fluorescent gel images of the attenuated *H. meleagridis* culture

**Figure 4:**
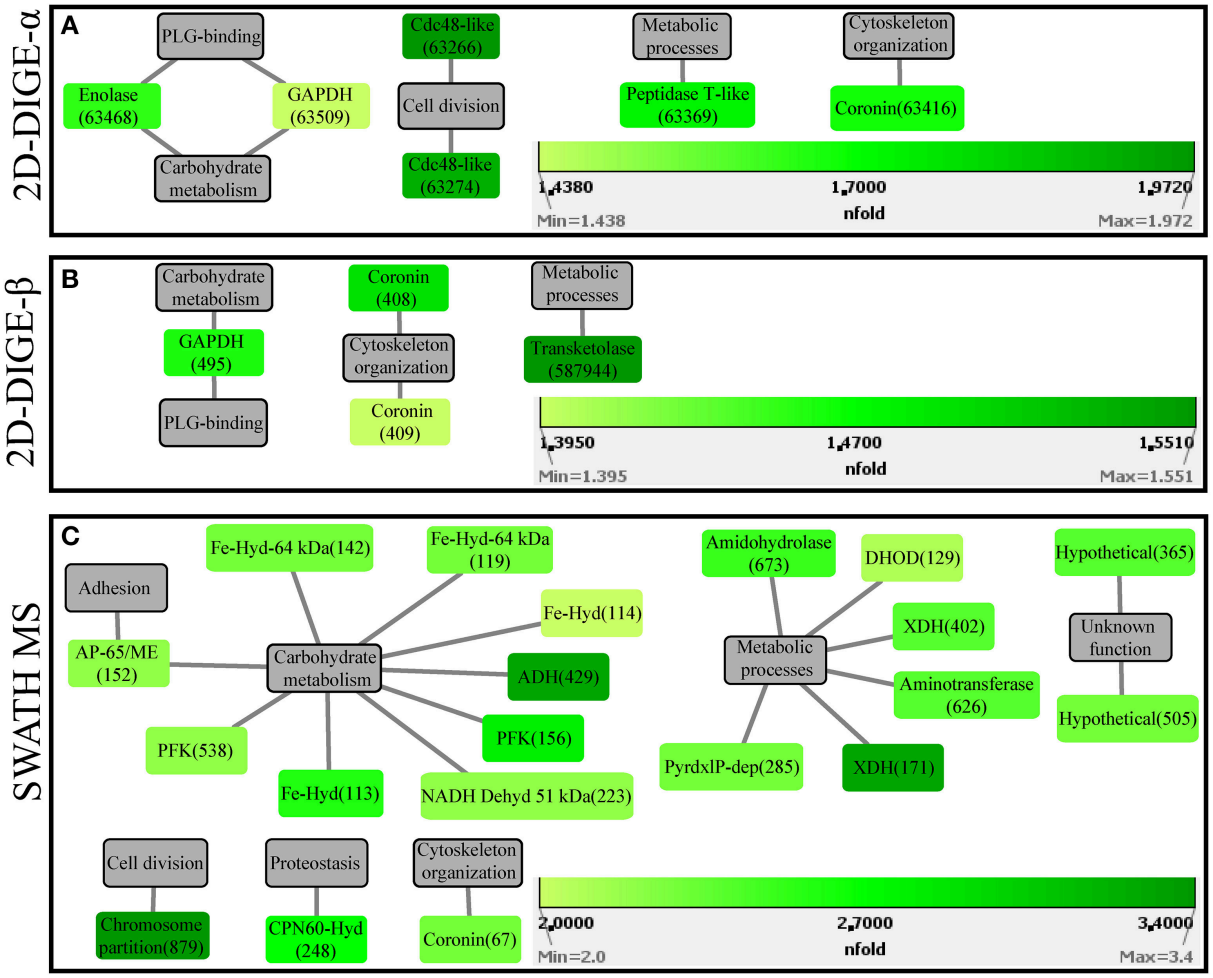
Protein-function network graphs of *Histomonas meleagridis*-specific proteins detected as significantly overexpressed in the attenuated strain by the 2D-DIGE-α **(A)**, 2D-DIGE-β **(B)**, and SWATH MS **(C)** experiments. The protein identifications are displayed as color-coded source nodes, with their edges connected to gray colored target nodes which represent the proposed functions. The numbers in parentheses represent the identification numbers assigned to each protein by Delta2D software version 4.7 (Decodon GmbH, Greifswald, Germany) and SWATH MS experiment. In each panel, the dialogs on the right lower corners are the green colored gradient which was selected to represent the fold upregulation values of overexpressed proteins from the attenuated strain. The source nodes were color-coded based on the fold upregulation data associated with each significantly differentially expressed protein (Tables 2–4). The network layout was manually determined. The reader is referred to Tables 2–4 for explanation of abbreviated protein names. 2D-DIGE, two-dimensional differential gel electrophoresis; SWATH MS, sequential window acquisition of all theoretical mass spectra mass spectrometry.

In the 2D-DIGE-α experiment, six out of the seven analyzed spots, which were upregulated in the gel images of the attenuated culture, corresponded to five proteins of the protozoan (Table 2). These proteins can be connected with five different categories (Figure 4A). As demonstrated by the protein network, the protein spots with the highest upregulation were identified as a Cdc48-like protein (63266, 63274) and a Clan MH, family M20, peptidase T-like metallopeptidase (63369) (Table 2, Figure 4A). The two Cdc48-like protein spots were found to be closely associated at a *M*_r_ of ~110 kDa (Figures S9Ai,Aii). In contrast to the virulent strain, GAPDH was identified in a single significantly upregulated spot (63509), which was located at a position that corresponded to its calculated pI (Table 2 and Figures S9Ai,Aii). The same single GAPDH spot was also visible in the gel images of the virulent culture at the same pI value (Figures S8Ai,Aii), albeit in the attenuated strain it exhibited a slightly higher upregulation (Table 2). The only spot that did not belong to the protozoan was identified as bovine serum albumin (Table 2). This spot exhibited the highest upregulation in the gel images of the attenuated strain but its presence originated from the culture medium (Table 2).

In the 2D-DIGE-β group, four out of five spots, which demonstrated upregulation in the gel images of the attenuated culture, represented three proteins of *H. meleagridis* (Table 3). These proteins can be associated with four different categories (Figure 4B). As shown in the protein network, the protein spots with the highest upregulation were a transketolase family protein (587944) and coronin (408) (Table 3 and Figure 4B). Coronin was also identified in a second spot (409) but was mapped to a different contig (Table 3). The two coronin spots displayed similar fold upregulation values and were visible, as a pair, at a position that corresponded to their correct pI but at ~20 kDa higher than their calculated *M*_r_ (Figures S9Bi,Bii). The same prominent GAPDH protein spot (495), which displayed a significant upregulation in the 2D-DIGE-α experiment, was also detected as overexpressed here at a position that corresponded to its correct pI (Table 3 and Figures S9Bi,Bii). Due to its size and position, this spot was distinguishable from its post-translational modifications which were upregulated in the gel images of the virulent culture (Figures S8Bi,Bii). In contrast with the findings of the 2D-DIGE-α experiment for the attenuated strain, one overexpressed spot exhibited homology with an *E. coli* protein and was associated with purine metabolism (Table 3).

### Identification of proteins detected as significantly differentially regulated by SWATH MS

An average number of 832 and 878 proteins were identified in the protein samples which were obtained from the virulent (25a and 25b) and attenuated (303a and 303b) *H. meleagridis* culture, respectively. The statistical analysis detected 42 significantly differentially expressed proteins of which four proteins fulfilled the significance criteria of MarkerView, 10 proteins fulfilled the criteria of R programming language and 28 proteins were detected as statistical significant by both methods (Table 4). From these differentially regulated proteins, 22 originated from the virulent and 20 from the attenuated *H. meleagridis* culture (Table 4, Table S3).

**Table 4:** Identification and quantification of significant differentially expressed proteins from the cultivated virulent (25) and attenuated (303) *Histomonas meleagridis* strain by SWATH MS.

**Protein Nr.^a^**	**Protein identity- Contig ID^b^; Accession Nr**.	**Protein sample^c^**	**Fold upregulation**	**Peptide Nr**.	**Statistics^d^**
**PEPTIDASE ACTIVITY**					
220	Clan CA, family C1, cathepsin L-like cysteine peptidase (Cathepsin L-like)- Contig2131; HAGI01002930	25	10.1	6	R
124	Clan CA, family C1, cathepsin L-like cysteine peptidase (Cathepsin L-like)- Contig2161; HAGI01002235	25	5.0	6	R+MV
512	LysM peptidoglycan domain-containing protein (LysM)- Contig1659; HAGI01001637	25	4.4	4	R+MV
229	Clan CD, family C13, asparaginyl endopeptidase-like cysteine peptidase (Peptidase C13)- Contig00252_LP; HAGI01002770	25	4.2	6	R
507	Basic secretory protein (BSP) family protein- Contig857; HAGI01000846	25	3.9	5	R
289	Clan CA, family C1, cathepsin L-like cysteine peptidase (Cathepsin L-like)- Contig1674; HAGI01001652	25	3.8	6	R
323	NlpC/P60 superfamily cysteine peptidase domain-containing protein (NlpC/P60)- Contig1788; HAGI01001764	25	3.4	6	R
753	NlpC/P60 superfamily cysteine peptidase domain-containing protein (NlpC/P60)- Contig04155_HP; HAGI01002646	25	2.3	4	R+MV
**UNKNOWN FUNCTION**					
907	Hypothetical protein- Contig04355_LP; HAGI01003301	25	17.8	2	R+MV
637	Hypothetical protein- Contig02848_HP; HAGI01002296	25	3.3	3	R+MV
242	Hypothetical protein- Contig593; HAGI01000587	25	2.8	6	R+MV
872	Hypothetical protein- Contig03282_HP; HAGI01002399	25	2.2	2	MV
**CYTOSKELETON ORGANIZATION**					
108	α-Actinin- Contig328; HAGI01000323	25	4.4	6	R+MV
625	Actin-binding protein (ABP)- Contig497; HAGI01000491	25	2.3	4	R+MV
**METABOLIC PROCESSES**					
329	F420-0-gamma-glutamyl ligase (CofE-AF)- Contig02484_HP; HAGI01002231	25	5.3	6	R+MV
**CALCIUM ION BINDING**					
406	EF hand family protein- Contig2024; HAGI01001994	25	3.6	5	R+MV
**LIPID METABOLIC PROCESS/PORE-FORMING PROTEINS**					
984	Surfactant protein B-like (SPB-like)- Contig03153_LP; HAGI01003015	25	2.9	2	R
**PROTEIN SYNTHESIS**					
788	60S acidic ribosomal protein P1 (60S)- Contig03716_HP; HAGI01002520	25	2.4	2	R
**CARBOHYDRATE METABOLISM**					
258	Adenylate kinase family protein (ADK)- Contig04065_HP; HAGI01002611	25	2.2	6	R+MV
**MEMBRANE TRAFFICKING**					
716	C2 domain-containing protein-Contig1639; HAGI01001617	25	2.2	3	R
**VESICLE FUSION**					
584	SNARE domain-containing protein- Contig04118_HP; HAGI01002630	25	2.1	4	R+MV
**SIGNALING**					
341	G-protein α subunit (Gprotein α)- Contig1461; HAGI01001440	25	2.0	5	R+MV
**CARBOHYDRATE METABOLISM**					
429	Alcohol dehydrogenase iron-containing family protein (ADH)- Contig246; HAGI01000245	303	3.3	6	R+MV
156	Phosphofructokinase family protein (PFK)- Contig1563; HAGI01001541	303	2.8	5	MV
113	Iron hydrogenase (Fe-Hyd)- Contig1190; HAGI01001174	303	2.6	6	R+MV
119	Iron hydrogenase 64 kDa (Fe-Hyd-64 kDa)- Contig837; HAGI01000826	303	2.3	6	R+MV
142	Iron hydrogenase 64 kDa (Fe-Hyd-64 kDa)- Contig1051; HAGI01001036	303	2.3	6	R+MV
223	NADH dehydrogenase 51kDa (NADH Dehyd 51 kDa)- Contig292; HAGI01000290	303	2.2	6	R+MV
538	Phosphofructokinase family protein (PFK)- Contig570; HAGI01000564	303	2.2	3	MV
114	Iron hydrogenase (Fe-Hyd)- Contig2186; HAGI01002119	303	2.0	6	R+MV
**METABOLIC PROCESSES**					
171	Xanthine dehydrogenase (XDH)- Contig708; HAGI01000700	303	3.3	6	R+MV
673	Amidohydrolase family protein- Contig243; HAGI01000242	303	2.5	3	R+MV
402	Xanthine dehydrogenase (XDH)- Contig19; HAGI01000019	303	2.4	6	R
626	Aminotransferase classes I and II family protein Contig03239_HP; HAGI01002389	303	2.4	2	R+MV
285	Pyridoxal-phosphate dependent enzyme family protein (PyrdxlP-dep)- Contig334; HAGI01000329	303	2.3	6	MV
129	Dihydroorotate dehydrogenase family protein (DHOD)- Contig290; HAGI01000288	303	2.1	6	R+MV
**UNKNOWN FUNCTION**					
365	Hypothetical protein- Contig634; HAGI01000628	303	2.4	6	R+MV
505	Hypothetical protein- Contig2180; HAGI01002115	303	2.3	5	R+MV
**CELL DIVISION**					
879	Chromosome partitioning protein ATPase- Contig1699; HAGI01001677	303	3.4	2	R
**PROTEOSTASIS**					
248	Chaperonin CPN60 hydrogenosomal (CPN60-Hyd)- Contig1546; HAGI01001524	303	2.7	6	R+MV
**CYTOSKELETON ORGANIZATION**					
67	Coronin putative- Contig1058; HAGI01001043	303	2.3	6	R+MV
**ADHESION/CARBOHYDRATE METABOLISM**					
152	Adhesin protein AP-65 / malic enzyme (AP-65/ME)- Contig2156; HAGI01002133	303	2.2	6	R+MV

*The differentially expressed proteins are categorized according to their proposed functions and listed within categories according to their fold upregulation (from high to low)*.

*Proteins that take part in metabolic processes other than carbohydrate metabolism are grouped together*.

a *Protein number assigned by sequential window acquisition of all theoretical mass spectra (SWATH) MS*.

b *Contig identification number (ID) was obtained from the de novo transcriptome sequencing of a virulent and an attenuated H. meleagridis strain (Mazumdar et al., 2017). HP = the extension was assigned to contigs specific to the attenuated strain. LP = the extension was assigned to contigs specific to the virulent strain*.

c *Protein sample = 25: the identified protein was significantly upregulated in protein samples of the cultivated virulent strain*.

*303: the identified proteins was significantly upregulated in protein samples of the cultivated attenuated strain*.

d *Statistics = R: proteins that fulfill the significance criteria of R programming language. MV: proteins that fulfill the significance criteria of MarkerView. R+MV: proteins that fulfill the significance criteria of R programming language and MarkerView*.

The fold change in upregulation values ranged between 2.0 and 17.8 for proteins of the virulent strain and between 2.0 and 3.4 for proteins of the attenuated one (Table 4 and Figures 3C, 4C). The 22 upregulated proteins in the virulent culture corresponded to 16 different proteins, one of which was also found to be significantly overexpressed in the 2D-DIGE-α experiments and identified as Clan CD, family C13, asparaginyl endopeptidase-like CP (Figure 2A). Based on their proposed functions, the 22 proteins could be connected with 12 different categories (Figure 3C). Peptidase activity was the largest category, which was represented by eight identifications each mapped to a different contig (Table 4, Figure 3C). The most frequent identification was a group of four proteins with unknown function termed hypothetical, followed by clan CA, family C1, cathepsin L-like CP which was represented by three proteins (Table 4, Figure 3C). The four hypothetical proteins were encoded by different contigs and that was also the case for the three proteins identified as cathepsin L-like CP (Table 4). Proteins with the highest upregulation were a hypothetical protein (907) and a clan CA, family C1, cathepsin L-like CP (220). In this strain, most of the identified proteins displayed a mediocre fold upregulation level which was below the median value (Table 4).

The 20 overexpressed proteins in samples of the attenuated culture represented 15 different proteins, out of which only coronin was shown to be also significantly upregulated in the gel-based experiments (Table 4, Figure 2B). The upregulated coronin protein, reported by SWATH MS, was mapped to a contig which was different from the one to which the coronins, reported by the 2D-DIGE experiments, were mapped (Tables 2–4). The 20 overexpressed proteins could be connected with seven different categories based on their proposed functions (Figure 4C). Carbohydrate metabolism constituted the largest category, which was represented by nine identifications each mapped to a different contig (Table 4, Figure 4C). Within this group, iron hydrogenases were the most frequently identified proteins (Figure 4C). The four iron hydrogenases were encoded by different contigs and could be separated into two groups based on their annotation (Table 4). The second largest category was a group of six proteins that are involved in other metabolic processes (Figure 4C). The range of fold upregulation values for these 20 proteins was narrower in comparison to the one reported for the virulent strain (Table 4). Nevertheless, as for the virulent strain, most of the 20 proteins from the attenuated one demonstrated mediocre upregulation which was below the median value (Table 4, Figure 4C). Proteins with the highest upregulation belonged to categories such as cell division (879), carbohydrate metabolism (429), and metabolic processes (171) (Table 4, Figure 4C).

In contrast to the gel-based experiments, none of the above 42 significantly differentially expressed proteins belonged to *E. coli* DH5α. However, an average number of 151 and 158 proteins were identified to be of bacterial origin in protein samples obtained from the *in vitro* cultivated virulent (25a and 25b) and attenuated (303a and 303b) strains, respectively.

## Discussion

### 2-DE and 2D-DIGE experiments

Despite substantial differences between 2-DE and 2D-DIGE, such as in the experimental design, the applied amount of protein, influence of technical variation and protein spot detection, the protein patterns obtained by the two techniques showed a high level of similarity. The observed distribution of protein spots in the 2D-DIGE-α and 2D-DIGE-β experiments was in agreement with previously published conventional 2-DE gels of *H. meleagridis* cultures (Monoyios et al., 2018). In the conventional 2-DE study as well as in the 2D-DIGE, a noticeable difference in the *M*_r_ distribution of proteins between virulent and attenuated culture samples was revealed (Monoyios et al., 2018). In particular, low *M*_r_ proteins were more numerous in the gel images of the virulent strain, while high *M*_r_ proteins were more abundant in the gel images of the attenuated one. This could be attributed to a stronger proteolytic activity that might have occurred within the virulent *H. meleagridis* parasites. Evidence for this, could be found in the shotgun proteomic analysis, in which a number of CPs were found to be significantly upregulated in protein samples of the virulent strain, one of which exhibited the second highest upregulation value among all others.

A number of proteins which were found to be significantly overexpressed in the 2-DE study were re-detected in the two 2D-DIGE experiments, a finding that underscores their importance. Interestingly, these proteins were also mapped to the same contigs. In the virulent strain proteins such as 14-3-3, Hsp70, multiple actin proteins of low *M*_r_ and the legumain-like CP were re-discovered as significantly upregulated. The latter protein was also detected as upregulated in the same strain by the SWATH MS experiment but was mapped to a different contig. Nevertheless, knowing that this CP was consistently detected in the virulent strain by three independent proteomic experiments verifies its implication in virulence mechanisms. On the other hand, in the fluorescent gel images of the attenuated strain, the Cdc48-like protein, enolase, and coronin were re-detected. Coronin, a protein implicated in actin cytoskeleton remodeling, was also found overexpressed in the attenuated strain by the SWATH MS experiment but mapped to a different contig.

### 2D-DIGE and SWATH MS experiments

Notwithstanding their utility, gel-based techniques cannot visualize complete proteomes (reviewed in Rabilloud et al., 2010). In a given 2-DE gel only specific proteins can be visualized depending on a number of factors such as: solubility, pI, relative abundance, *M*_r_, gel size, and pH gradient used (Görg et al., 2004; reviewed in Baggerman et al., 2005). The utilization of a gel-free, label-free shotgun proteomic approach with SWATH MS technology complements the 2D-DIGE gel-based experiments, since the resolution and detection of significant proteins is not depended on the pH gradient of the IPG strip, the length of the IPG strip or the polyacrylamide gel. A characteristic observed in our study as well. However, in spite of their resolving power, shotgun proteomic methods are preoccupied with the analysis of peptides and cannot conclude if a given identified protein was subjected to proteolysis or post-translational modifications as information on their experimental pI and *M*_r_ is lost (Görg et al., 2004). This feature was observed in the case of actin and GAPDH identifications, whose multiple spots were found upregulated in the virulent strain in both 2D-DIGE experiments, but SWATH analysis missed to detect them.

### Proteins with plasminogen and fibronectin-binding potential

Although they are traditionally connected with non-virulent mechanisms, proteins such as actin, GAPDH and FBAL demonstrated the highest upregulation in fluorescent gel images of the virulent strain. Actin is an essential component of cytoskeleton with involvement in cell motility and cellular shape maintenance (reviewed in Dominguez and Holmes, 2011). In anaerobic protozoa such as *Trichomonas vaginalis*, GAPDH and FBAL participate in the glycolytic part of carbohydrate metabolism, which takes place in the cytoplasm (Huang et al., 2014).

The upregulated actin and GAPDH proteins displayed pI and *M*_r_ values, which were different from those displayed by the prominent actin and GAPDH proteins that were visualized on the same gels. Interestingly, regardless of their gel position, the actin and GAPDH proteins were all mapped to the same contigs. This finding supports the view that these multiple post-translationally modified or truncated proteins might be components of other mechanisms which are not related with cytoskeleton or carbohydrate metabolism. The experiments of Lama and coworkers demonstrated that GAPDH is a fibronectin (FN)-, plasminogen (PLG)- and collagen-binding protein with localization on *T. vaginalis* surface (Lama et al., 2009). Insoluble FN is a large glycoprotein of the extracellular matrix (ECM) that connects, via integral membrane proteins known as integrins, cellular surfaces with collagen (Hynes and Yamada, 1982; Bachman et al., 2015). It was theorized that the association of *T. vaginalis* with ECM components can promote colonization of the host (Lama et al., 2009).

Common characteristics of actin, FBAL and GAPDH are their alternative localization on parasites' surface and the ability to bind PLG (Lama et al., 2009; González-Miguel et al., 2015). PLG binding to these surface proteins, is an important step for its activation to plasmin and for maintaining its activation (reviewed in Plow et al., 1995). Lysine residues that are externally localized in PLG surface receptors are usually implicated in PLG binding (González-Miguel et al., 2015). Interestingly, in *H. meleagridis* actin the presence of a domain with similarities to a proposed PLG-binding domain of other organisms was discussed (González-Miguel et al., 2015; Monoyios et al., 2018). Plasmin degrades ECM and can help parasites that reside in blood vessels such as *Dirofilaria immitis*, to avoid blood clots due to its fibrinolytic activity (González-Miguel et al., 2015). Considering that *H. meleagridis* can spread via blood vessels to the liver and to other organs during the final stages of the disease (Singh et al., 2008), the ability to bind PLG might be of equal importance for this flagellate.

### Cytolytic factors

The SWATH MS experiment identified a pore-forming protein (PFP), namely surfactant protein B-like (SPB-like), being overexpressed in the virulent strain. SPB contains a motif of six cysteine periodically located residues, which is commonly found in proteins of the saposin-like family (reviewed in Hawgood et al., 1998). This saposin motif is also found in molecules with antibacterial activity such as the lysin of Natural Killer cells (NK-lysin) and the PFPs of *Entamoeba histolytica*, termed amoebapores (reviewed in Hawgood et al., 1998). In addition to structural similarities, the proteins mentioned above, demonstrate similar mode of action. This involves attachment to membranes that possess negatively charged phospholipids, following the lysis or fusion of such membranes (reviewed in Hawgood et al., 1998). Once PFPs penetrate the lipid bilayer of the target cell, death occurs by osmotic lysis (reviewed in Hirt et al., 2011). The activity of PFPs depends on the pH of the micro-space which is created between the parasite and the host's cell, following amoeboid transformation and cytoadherence (reviewed in Hirt et al., 2011). Based on the above, it is evident that SPB-like can be an effective virulence factor. Nevertheless, in the *in vitro* environment the protein might also contribute to the lysis of bacteria and through this to nutrient acquisition.

A clan CA, family C1, cathepsin L-like and a clan CD, family C13, asparaginyl endopeptidase-like CPs were significantly upregulated in the virulent low-passaged *H. meleagridis* parasite. In *T. vaginalis* almost half of the predicted peptidases contain cysteine as an active site similar to *H. meleagridis* transcriptome in which CPs were the most represented group of peptidases (reviewed in Hirt et al., 2011; Mazumdar et al., 2017). This category of peptidases participates in *T. vaginalis* virulence mechanisms such as cytoadherence, nutrient acquisition, hemolysis and apoptosis of human vaginal epithelial cells (reviewed in Hirt et al., 2011). Aside from this, the proteolytic activity of cathepsin L-like CPs, secreted by *Trichomonas gallinae*, was connected to the cytopathogenic effect on permanent chicken liver cells indicating their role in virulence of this avian parasite (Amin et al., 2012). The differential expression of cathepsin L-like CP was detected comparing 2-DE gels of high- and low-virulence *T. vaginalis* isolates (De Jesus et al., 2009). However, in contrast to our finding, the *T. vaginalis* clan CD, family C13, asparaginyl endopeptidase-like CP did not exhibit differential expression among these isolates (De Jesus et al., 2009). It was theorized that *T. vaginalis* CPs are involved in the lysis of erythrocytes and through this action can obtain lipids and iron, which cannot be synthesized otherwise (Rosset et al., 2002). Considering, that the *in vitro* growth of *H. meleagridis* can be significantly enhanced by the presence of supplemental cholesterol (Gruber et al., 2018) one cannot exclude the involvement of CPs in similar processes.

Rab11-isotypes, one of which was detected to be overexpressed in the virulent strain by 2D-DIGE, could be implicated in the secretion of CPs. *E. histolytica* parasites which were overexpressing a Rab11 isotype, demonstrated augmentation in the amounts of intracellular and secreted CPs (Mitra et al., 2007). The overexpression of this isotype, was not connected with higher production of CPs, but rather with an increase in transportation (Mitra et al., 2007). While Rab-GTPases modulate the contact of membranes, SNARE proteins participate in the actual act of fusion and lipid bilayer mixing (Ungermann and Langosch, 2005). In this context, the detection of an overexpressed SNARE domain-containing protein in the virulent parasite could increase the fusion of CP-containing vesicles with the plasma membrane and the release of CPs.

### Cytoadherence mechanisms

The SWATH experiment identified the α subunit of the heterotrimeric membrane-associated G protein as upregulated in the virulent strain. The G protein subunits can activate other molecules, including phospholipase C (InterPro entry^5^). The activity of phospholipase C on phospholipids can alter the negative surface charge of *T. vaginalis*, and as a result the interaction of this human parasite with the host's cells is initiated (reviewed in López et al., 2000). Additionally, the creation of cytoplasmic extensions such as pseudopodia and filopodia is necessary for initiating parasite contact with epithelial surfaces and for the maintenance of cytoadhesion (reviewed in López et al., 2000). In addition to the above described mechanisms, proteins such as adhesins and CPs directly contribute to the parasite's cytoadherence (reviewed in López et al., 2000). It was theorized that the activity of CPs on cell surface molecules can elicit the synthesis of adhesins (reviewed in López et al., 2000). Nonetheless, one of the five known adhesin proteins, called AP65, was detected as overexpressed in the attenuated *H. meleagridis* strain by the SWATH experiment. It should be mentioned that AP65 is also a malic enzyme and the presence of an initial peptide sequence can direct this protein to the hydrogenosome where it exhibits a different function (Alderete et al., 1995; Engbring and Alderete, 1998). Taking into account that several other hydrogenosomal enzymes were detected as overexpressed in the attenuated parasite, the hydrogenosomal function of the above protein seems more likely.

### Survival mechanisms

Two NlpC/P60 superfamily domain-containing proteins showed increased expression in the virulent *H. meleagridis* parasite by the SWATH analysis. *T. vaginalis* possesses nine Clan CA, family C40 NlpC/P60 papain-like peptidases of the cysteine type that are involved in the cleavage of bacterial cell walls (Carlton et al., 2010; reviewed in Hirt et al., 2011). Considering that *H. meleagridis* utilizes co-cultivated *E. coli* DH5α as food source, proteins like the above might contribute to its *in vitro* survival. In the same strain, the SWATH analysis identified an overexpressed lysin motif (LysM)-containing protein. This motif is found in enzymes that, in addition to chitin-binding, also degrade bacterial cell walls (InterPro entry^6^). Additionally, the LysM-containing protein, possesses a glycoside hydrolase 19 domain, typically found in chitinases (InterPro entry^7^). The cyst of the protozoan parasite *Entamoeba* spp. is encircled with chitin and its main element are proteins with chitin-binding domains, such as chitinases (Van Dellen et al., 2006). Under nutritional restricted conditions amoeboid *T. vaginalis* parasites, undergo a pseudocyst stage characterized by morphological changes such as round shape and internalized flagella (De Jesus et al., 2007). On the protein level, *T. vaginalis* pseudocyst stages were consistently overexpressing several actin proteins which were mapped to a single accession, PEPCK and a Rab11 isotype, whereas the expression of hydrogenosomal enzymes was shut down (De Jesus et al., 2007).

The overexpression of the above proteins in the gel images of the virulent *H. meleagridis* was accompanied by the absence of hydrogenosomal enzyme overexpression. In recent detailed investigations on *H. meleagridis* morphology, a genuine encystation process was not observed (Gruber et al., 2017). However, as it was the case for *T. vaginalis*, the flagellate was capable of defending itself by forming cyst-like stages under adverse *in vitro* conditions characterized by rounded morphology and double cell membrane (Zaragatzki et al., 2010; Gruber et al., 2017). The upregulation of the above proteins in the virulent parasite, which is less adapted to the *in vitro* environment, indicates the existence of these defense mechanisms on a protein level.

### Glycolytic and hydrogenosomal proteins of the carbohydrate metabolism

Overall, six enzymes of the glycolytic pathway (7 GAPDH spots, 5 PEPCK spots, 1 FBAL spot, 1 PPDK spot, 1 ADH spot, 1 PGlyM spot) and one protein (ADK protein) that acts in the hydrogenosome were shown to be overexpressed in the virulent strain by the gel-based and gel-free experiments. Except from the single GAPDH spot and ADH, the detected overexpression of two other glycolytic proteins (2 PFK proteins, 1 enolase spot), as well as six hydrogenosomal enzymes (2 iron hydrogenase 64 kDa proteins, 2 iron hydrogenase proteins, 1 NADH dehydrogenase 51 kDa protein, 1 malic enzyme) was the exclusive property of the attenuated parasite.

In amitochondriate anaerobic protozoa, such as *T. vaginalis* and *H. meleagridis*, carbohydrate metabolism is fermentative. The part of the glycolytic pathway that takes place in the cytoplasm results in pyruvate or malate production, which are further converted to hydrogen, carbon dioxide and acetate in double-membrane organelles called hydrogenosomes (De Jesus et al., 2007). Key hydrogenosomal enzymes that are involved in the latter process are the pyruvate ferredoxin oxidoreductase (PFOR), electron transporter ferredoxin (Fd), and hydrogenase (reviewed in Müller, 1993). Hydrogenosomal enzymes contain iron-sulfur clusters and iron is considered to be an essential element for their function, structure and transcription (De Jesus et al., 2007). Iron depletion can lead to the downregulation of several *T. vaginalis* hydrogenosomal enzymes, including Iron hydrogenase-1 (50 kDa), -2 (64 kDa), and -3 (Beltrán et al., 2013). Interestingly, in this human parasite the downregulation of hydrogenosomal enzymes due to iron starvation was accompanied by an upregulation of certain glycolytic enzymes, including PEPCK (De Jesus et al., 2007), which was detected as overexpressed at multiple gel positions in the virulent *H. meleagridis*. The authors theorized that the overexpression of PEPCK under such conditions by *T. vaginalis* is part of an energy compensation mechanism (De Jesus et al., 2007), and one could argue that the same could apply for the virulent *H. meleagridis*. However, under *in vitro* conditions *H. meleagridis* should not experience a compromised hydrogenosomal activity due to iron deprivation since the fetal bovine serum, which is used as nutritional element, contains high levels of the iron-storage protein ferritin (Kakuta et al., 1997). Nonetheless, the *in vitro* environment might not be entirely optimal for this microaerophilic protozoan, considering that after each passage *H. meleagridis* comes in contact with atmospheric oxygen. It is known that *T. vaginalis* is sensitive to oxygen levels above those normally encountered in the host's vaginal environment (Ellis et al., 1994). In other trichomonads, such as *Tritrichomonas foetus*, the activities of PFOR and iron hydrogenase were negatively affected by oxygen with the latter showing higher sensitivity compared to the former (Lindmark and Müller, 1973). On the transcriptome level, oxygen protective molecules were found in both *H. meleagridis* strains, albeit the sequence encoding for superoxide dismutase (SOD), which is an important oxidative stress defense in *T. vaginalis*, was only reported in the attenuated one (Ellis et al., 1994; Mazumdar et al., 2017). Based on the above, the detected overexpression of hydrogenosomal enzymes in the attenuated strain might indicate the ability of this strain to cope better with the *in vitro* fluctuating oxygen levels. Alternatively, the fact that the overexpressed iron hydrogenases were mapped to different contigs can itself constitute a coping mechanism that protects hydrogenosomal metabolism from oxygen and might explain the higher adaptation of this strain to the *in vitro* environment.

## Conclusion

This study corroborated, and to a greater extent supplemented the findings of the first *H. meleagridis* proteomic analysis that utilized conventional 2-DE (Monoyios et al., 2018). Results obtained from both 2D-DIGE experiments overlapped in the majority of identified proteins; however the SWATH MS analysis contributed to the identification of a completely new set of proteins. Overall, both gel-based and gel-free investigations displayed the poor adaptation of the low-passaged virulent strain to the *in vitro* conditions in comparison to the high-passaged attenuated strain. This was manifested by the upregulation of proteins related with stress response in the former and the overexpression of cell division proteins in the latter. Additionally, the upregulation of different components of the carbohydrate metabolism between these strains might further support this view. Molecules that are part of the virulence arsenal of other related parasites such as CPs and PFPs were detected in the virulent strain, a feature in large part identified by the SWATH MS experiment. Finally, both gel-based experiments identified multiple modifications of ubiquitous proteins such as actin and GAPDH in the virulent strain, which might indicate alternative functions and localizations. This finding was supported by the conventional 2-DE comparative study and should be addressed in greater detail by future investigations.

## Data availability

The mass spectrometry proteomic data were deposited to the ProteomeXchange Consortium via the PRIDE (Vizcaíno et al., 2016) partner-repository with the following accession numbers: PXD009332 for 2D-DIGE spot data obtained by MALDI-TOF/TOF, PXD009316 for 2D-DIGE spot data obtained by LC-MS/MS and PXD009319 for the SWATH MS data and will be made publicly available.

Any other data supporting the conclusions of this manuscript can be made available by the authors, without undue reservation, to any qualified researcher upon request.

## Author contributions

IB, AM, and MH contributed conception and design of the study. MP assisted with 2D-Gels. KN, KH, and SS performed mass spectrometry analysis and wrote some material sections of the manuscript. AM, KN, and KH organized the database and performed the statistical analysis. AM and IB wrote the first draft of the manuscript. All authors contributed to manuscript revision, read, and approved the submitted version.

### Conflict of interest statement

The authors declare that the research was conducted in the absence of any commercial or financial relationships that could be construed as a potential conflict of interest.

## Footnotes

^1^http://www.uniprot.org/

^2^http://www.ebi.ac.uk/ena/data/view/PRJEB19109

^3^ftp://ftp.thegpm.org/fasta/cRAP/crap.fasta

^4^https://www.promega.com/-/media/files/resources/protocols/technical-manuals/101/trypsinlysc-mix-mass-spec-grade-protocol.pdf?la=en

^5^http://www.ebi.ac.uk/interpro/entry/IPR000367

^6^https://www.ebi.ac.uk/interpro/entry/IPR018392

^7^https://www.ebi.ac.uk/interpro/entry/IPR000726

## Abbreviations

2-DE: two-dimensional electrophoresis
2D-DIGE: two-dimensional differential gel electrophoresis
DTT: dithiothreitol
ECM: extracellular matrix
ESI: electrospray ionization
FBAL: fructose-bisphosphate aldolase
Fd: ferredoxin
FDR: false discovery rate
FN: fibronectin
GAPDH: glyceraldehyde-3-phosphate dehydrogenase
HPLC: high performance liquid chromatography
IAA: iodoacetamide
IEF: isoelectric focusing
IPG: immobilized pH gradient
IS: internal standard
LC-MS/MS: liquid chromatography coupled to tandem mass spectrometry
MALDI-TOF/TOF MS: matrix-assisted laser desorption/ionization time-of-flight/time-of-flight mass spectrometry
*M*_r_: molecular mass
NK: Natural killer cells
PEPCK: phosphoenolpyruvate carboxykinase
PFOR: pyruvate ferredoxin oxidoreductase
PFP: pore-forming protein
PLG: plasminogen
SDS-PAGE: sodium dodecyl sulfate polyacrylamide gel electrophoresis
SOD: superoxide dismutase
SPB: surfactant protein B
SWATH: sequential window acquisition of all theoretical mass spectra.

## References

[B1] Alderete J. F. O'Brien J. L. Arroyo R. Engbring J. A. Musatovova O. Lopez O.. (1995). Cloning and molecular characterization of two genes encoding adhesion proteins involved in *Trichomonas vaginalis* cytoadherence. Mol. Microbiol. 17, 69–83. 10.1111/j.1365-2958.1995.mmi_17010069.x 7476210

[B2] Amin A. Nöbauer K. Patzl M. Berger E. Hess M. Bilic I. (2012). Cysteine peptidases, secreted by *Trichomonas gallinae*, are involved in the cytopathogenic effects on a permanent chicken liver cell culture. PLoS ONE 7:e37417. 10.1371/journal.pone.0037417 22649527 PMC3359344

[B3] Arnal L. Grunert T. Cattelan N. de Gouw D. Villalba M. I. Serra D. O.. (2015). *Bordetella pertussis* isolates from argentinean whooping cough patients display enhanced biofilm formation capacity compared to Tohama I reference strain. Front. Microbiol. 6:1352. 10.3389/fmicb.2015.01352 26696973 PMC4672677

[B4] Bachman H. Nicosia J. Dysart M. Barker T. H. (2015). Utilizing fibronectin integrin-binding specificity to control cellular responses. Adv. Wound Care 4, 501–511. 10.1089/wound.2014.0621 26244106 PMC4505771

[B5] Baggerman G. Vierstraete E. De Loof A. Schoofs L. (2005). Gel-based versus gel-free proteomics: a review. Comb. Chem. High Throughput Screen. 8, 669–677. 10.2174/138620705774962490 16464155

[B6] Beltrán N. C. Horváthová L. Jedelský P. L. Šedinová M. Rada P. Marcinčiková M.. (2013). Iron-induced changes in the proteome of *Trichomonas vaginalis* hydrogenosomes. PLoS ONE 8:e65148. 10.1371/journal.pone.0065148 23741475 PMC3669245

[B7] Benjamini Y. Hochberg Y. (1995). Controlling the false discovery rate - a practical and powerful approach to multiple testing. J. R. Stat. Soc. 57, 289–300.

[B8] Berth M. Moser F. M. Kolbe M. Bernhardt J. (2007). The state of the art in the analysis of two-dimensional gel electrophoresis images. Appl. Microbiol. Biotechnol. 76, 1223–1243. 10.1007/s00253-007-1128-0 17713763 PMC2279157

[B9] Blum H. Gross H. J. Beier H. (1989). The expression of the TMV-specific 30-kDa protein in tobacco protoplasts is strongly and selectively enhanced by actinomycin. Virology 169, 51–61. 10.1016/0042-6822(89)90040-8 2466372

[B10] Carlton J. M. Malik S.-B. Sullivan S. A. Sicheritz-Ponten T. Tang P. Hirt R. P. (2010). The genome of *Trichomonas vaginalis* in Anaerobic Parasitic Protozoa: Genomics and Molecular Biology, eds Clark C. G. Johnson P. J. Adam R. D. (Norfolk: Caister Academic Press), 45–81.

[B11] Cepicka I. Hampl V. Kulda J. (2010). Critical taxonomic revision of parabasalids with description of one new genus and three new species. Protist 161, 400–433. 10.1016/j.protis.2009.11.005 20093080

[B12] Crutchfield C. A. Thomas S. N. Sokoll L. J. Chan D. W. (2016). Advances in mass spectrometry-based clinical biomarker discovery. Clin. Proteomics 13, 1–12. 10.1186/s12014-015-9102-9 26751220 PMC4705754

[B13] De Jesus J. B. Cuervo P. Britto C. Sabóia-Vahia L. Costa E Silva-Filho F. Borges-Veloso A.. (2009). Cysteine peptidase expression in *Trichomonas vaginalis* isolates displaying high- and low-virulence phenotypes. J. Proteome Res. 8, 1555–1564. 10.1021/pr8009066 19186947

[B14] De Jesus J. B. Cuervo P. Junqueira M. Britto C. Costa e Silva-Filho F. Soares M. J.. (2007). A further proteomic study on the effect of iron in the human pathogen *Trichomonas vaginalis*. Proteomics 7, 1961–1972. 10.1002/pmic.200600797 17514679

[B15] Dominguez R. Holmes K. C. (2011). Actin structure and function. Annu. Rev. Biophys. 40, 169–186. 10.1146/annurev-biophys-042910-155359 21314430 PMC3130349

[B16] Ellis J. E. Yarlett N. Cole D. Humphreys M. J. Lloyd D. (1994). Antioxidant defences in the microaerophilic protozoan *Trichomonas vaginalis*: comparison of metronidazole-resistant and sensitive strains. Microbiology 140, 2489–2494. 10.1099/13500872-140-9-2489 7952198

[B17] Engbring J. A. Alderete J. F. (1998). Three genes encode distinct AP33 proteins involved in *Trichomonas vaginalis* cytoadherence. Mol. Microbiol. 28, 305–313. 10.1046/j.1365-2958.1998.00784.x 9622355

[B18] Ganas P. Liebhart D. Glösmann M. Hess C. Hess M. (2012). *Escherichia coli* strongly supports the growth of *Histomonas meleagridis*, in a monoxenic culture, without influence on its pathogenicity. Int. J. Parasitol. 42, 893–901. 10.1016/j.ijpara.2012.07.007 22921600

[B19] Gharahdaghi F. Weinberg C. R. Meagher D. A. Imai B. S. Mische S. M. (1999). Mass spectrometric identification of proteins from silver-stained polyacrylamide gel: a method for the removal of silver ions to enhance sensitivity. Electrophoresis 20, 601–605. 10.1002/(SICI)1522-2683(19990301)20:3<601::AID-ELPS601>3.0.CO;2-6 10217175

[B20] Gillet L. C. Navarro P. Tate S. Röst H. Selevsek N. Reiter L.. (2012). Targeted data extraction of the MS/MS spectra generated by data-independent acquisition: a new concept for consistent and accurate proteome analysis. Mol. Cell. Proteomics 11, 1–17. 10.1074/mcp.O111.016717 22261725 PMC3433915

[B21] González-Miguel J. Morchón R. Siles-Lucas M. Simón F. (2015). Fibrinolysis and proliferative endarteritis: two related processes in chronic infections? The model of the blood-borne pathogen *Dirofilaria immitis*. PLoS ONE 10:e124445. 10.1371/journal.pone.0124445 25875022 PMC4395379

[B22] Görg A. Drews O. Lück C. Weiland F. Weiss W. (2009). 2-DE with IPGs. Electrophoresis 30, 122–132. 10.1002/elps.200900051 19441019

[B23] Görg A. Weiss W. Dunn M. J. (2004). Current two-dimensional electrophoresis technology for proteomics. Proteomics 4, 3665–3685. 10.1002/pmic.200401031 15543535

[B24] Gruber J. Ganas P. Hess M. (2017). Long-term *in vitro* cultivation of *Histomonas meleagridis* coincides with the dominance of a very distinct phenotype of the parasite exhibiting increased tenacity and improved cell yields. Parasitology 144, 1253–1263. 10.1017/S0031182017000646 28478784

[B25] Gruber J. Pletzer A. Hess M. (2018). Cholesterol supplementation improves growth rates of *Histomonas meleagridis in vitro*. Exp. Parasitol. 185, 53–61. 10.1016/j.exppara.2018.01.007 29317242

[B26] Hawgood S. Derrick M. Poulain F. (1998). Structure and properties of surfactant protein B. Biochim. Biophys. Acta 1408, 150–160. 10.1016/S0925-4439(98)00064-7 9813296

[B27] Hess M. (2017). Commensal or pathogen – a challenge to fulfil Koch's postulates. Br. Poult. Sci. 58, 1–12. 10.1080/00071668.2016.1245849 27724044 PMC5359748

[B28] Hess M. Kolbe T. Grabensteiner E. Prosl H. (2006). Clonal cultures of *Histomonas meleagridis, Tetratrichomonas gallinarum* and a *Blastocystis* sp. established through micromanipulation. Parasitology 133, 547–554. 10.1017/S0031182006000758 16854251

[B29] Hess M. Liebhart D. Bilic I. Ganas P. (2015). *Histomonas meleagridis*-new insights into an old pathogen. Vet. Parasitol. 208, 67–76. 10.1016/j.vetpar.2014.12.018 25576442

[B30] Hess M. McDougald L. R. (2013). Histomoniasis (Blackhead) and other protozoan diseases of the intestinal tract in Diseases of Poultry, ed Swayne D. E. (Ames, IA: Wiley-Blackwell; John Wiley & Sons, Inc publication), 1172–1178.

[B31] Hirt R. P. de Miguel N. Nakjang S. Dessi D. Liu Y. C. Diaz N. (2011). *Trichomonas vaginalis* pathobiology: new insights from the genome sequence in Advances in Parasitology, eds Rollinson D. Hay S. I. (London: Elsevier Ltd.), 87–140. 10.1016/B978-0-12-391429-3.00006-X 22137583

[B32] Huang K. Y. Chen Y. Y. M. Fang Y. K. Cheng W. H. Cheng C. C. Chen Y. C.. (2014). Adaptive responses to glucose restriction enhance cell survival, antioxidant capability, and autophagy of the protozoan parasite *Trichomonas vaginalis*. Biochim. Biophys. Acta 1840, 53–64. 10.1016/j.bbagen.2013.08.008 23958562

[B33] Huang Q. Yang L. Luo J. Guo L. Wang Z. Yang X.. (2015). SWATH enables precise label-free quantification on proteome scale. Proteomics 15, 1215–1223. 10.1002/pmic.201400270 25560523

[B34] Hynes R. O. Yamada K. M. (1982). Fibronectins: multifunctional modular glycoproteins. J. Cell Biol. 95, 369–377. 10.1083/jcb.95.2.369 6128348 PMC2112946

[B35] Jiménez C. R. Huang L. Qiu Y. Burlingame A. L. (2001). In-gel digestion of proteins for MALDI-MS fingerprint mapping in Current Protocols in Protein Science, eds Coligan J. E. Dunn B. M. Speicher D. W. Wingfield P. T. (Brooklyn, NY: John Wiley & Sons, Inc.), 16.4.1–16.4.5. 10.1002/0471140864.ps1604s14 18429131

[B36] Kakuta K. Orino K. Yamamoto S. Watanabe K. (1997). High levels of ferritin and its iron in fetal bovine serum. Comp. Biochem. Physiol. 118A, 165–169. 10.1016/S0300-9629(96)00403-3 9243818

[B37] Kumar G. Hummel K. Ahrens M. Menanteau-Ledouble S. Welch T. J. Eisenacher M.. (2016). Shotgun proteomic analysis of *Yersinia ruckeri* strains under normal and iron-limited conditions. Vet. Res. 47, 1–13. 10.1186/s13567-016-0384-3 27716418 PMC5054536

[B38] Lama A. Kucknoor A. Mundodi V. Alderete J. F. (2009). Glyceraldehyde-3-phosphate dehydrogenase is a surface-associated, fibronectin-binding protein of *Trichomonas vaginalis*. Infect. Immun. 77, 2703–2711. 10.1128/IAI.00157-09 19380472 PMC2708568

[B39] Liebhart D. Ganas P. Sulejmanovic T. Hess M. (2017). Histomonosis in poultry: previous and current strategies for prevention and therapy. Avian Pathol. 46, 1–18. 10.1080/03079457.2016.1229458 27624771

[B40] Liebhart D. Sulejmanovic T. Grafl B. Tichy A. Hess M. (2013). Vaccination against histomonosis prevents a drop in egg production in layers following challenge. Avian Pathol. 42, 79–84. 10.1080/03079457.2012.760841 23391185

[B41] Liebhart D. Zahoor M. A. Prokofieva I. Hess M. (2011). Safety of avirulent histomonads to be used as a vaccine determined in turkeys and chickens. Poult. Sci. 90, 996–1003. 10.3382/ps.2010-01255 21489945

[B42] Lindmark D. G. Müller M. (1973). Hydrogenosome, a cytoplasmic organelle of the anaerobic flagellate *Tritrichomonas foetus*, and its role in pyruvate metabolism. J. Biol. Chem. 248, 7724–7728. 4750424

[B43] López L. B. De Melo Braga M. B. López J. O. Arroyo R. Costa e Silva Filho F. (2000). Strategies by which some pathogenic trichomonads integrate diverse signals in the decision-making process. An. Acad. Bras. Cienc. 72, 173–186. 10.1590/S0001-37652000000200006 10932116

[B44] Mazumdar R. Endler L. Monoyios A. Hess M. Bilic I. (2017). Establishment of a *de novo* reference transcriptome of *Histomonas meleagridis* reveals basic insights about biological functions and potential pathogenic mechanisms of the parasite. Protist 168, 663–685. 10.1016/j.protis.2017.09.004 29107797

[B45] McDougald L. R. (2005). Blackhead disease (Histomoniasis) in poultry : a critical review. Avian Dis. 49, 462–476. 10.1637/7420-081005R.1 16404985

[B46] Miller I. (2012). Application of 2D DIGE in animal proteomics in Difference Gel Electrophoresis (DIGE): Methods and Protocols. Series: Methods in Molecular Biology, eds Cramer R. Westermeier R. (New York, NY; Dordrecht; Heidelberg; London: Humana Press; Springer Science+ Business Media, LLC), 373–396. 10.1007/978-1-61779-573-2_26 22311774

[B47] Miller I. Gemeiner M. (1992). Two-dimensional electrophoresis of cat sera: protein identification by cross reacting antibodies against human serum proteins. Electrophoresis 13, 450–453. 10.1002/elps.1150130193 1425559

[B48] Mitra B. N. Saito-Nakano Y. Nakada-Tsukui K. Sato D. Nozaki T. (2007). Rab11B small GTPase regulates secretion of cysteine proteases in the enteric protozoan parasite *Entamoeba histolytica*. Cell. Microbiol. 9, 2112–2125. 10.1111/j.1462-5822.2007.00941.x 17441984

[B49] Monoyios A. Patzl M. Schlosser S. Hess M. Bilic I. (2018). Unravelling the differences : comparative proteomic analysis of a clonal virulent and an attenuated *Histomonas meleagridis* strain. Int. J. Parasitol. 48, 145–157. 10.1016/j.ijpara.2017.08.017 29203214

[B50] Müller M. (1993). The hydrogenosome. J. Gen. Microbiol. 139, 2879–2889. 10.1099/00221287-139-12-2879 8126416

[B51] Oliveros J. C. (2015). Venny. An Interactive Tool for Comparing Lists With Venn's Diagrams. Available online at: http://bioinfogp.cnb.csic.es/tools/venny/index.html

[B52] Petrak J. Ivanek R. Toman O. Cmejla R. Cmejlova J. Vyoral D.. (2008). Déjà vu in proteomics. A hit parade of repeatedly identified differentially expressed proteins. Proteomics 8, 1744–1749. 10.1002/pmic.200700919 18442176

[B53] Pham A. D. Mast J. Magez S. Goddeeris B. M. Carpentier S. C. (2016). The enrichment of *Histomonas meleagridis* and its pathogen-specific protein analysis : a first step to shed light on its virulence. Avian Dis. 60, 628–636. 10.1637/11389-021016-Reg.1 27610722

[B54] Plow E. F. Herren T. Redlitz A. Miles L. A. Hoover-Plow J. L. (1995). The cell biology of the plasminogen system. FASEB J. 9, 939–945. 10.1096/fasebj.9.10.7615163 7615163

[B55] R Development Core Team (2014). R: A Language and Environment for Statistical Computing. R Foundation for Statistical Computing, Vienna, Austria. Available online at: http://www.R-project.org/

[B56] Rabilloud T. Chevallet M. Luche S. Lelong C. (2010). Two-dimensional gel electrophoresis in proteomics: past, present and future. J. Proteomics 73, 2064–2077. 10.1016/j.jprot.2010.05.016 20685252

[B57] Rosset I. Tasca T. Tessele P. M. De Carli G. A. (2002). Scanning electron microscopy in the investigation of the *in vitro* hemolytic activity of *Trichomonas vaginalis*. Parasitol. Res. 88, 356–359. 10.1007/s00436-001-0555-6 11999024

[B58] Shannon P. Markiel A. Ozier O. Baliga N. S. Wang J. T. Ramage D.. (2003). Cytoscape: a software environment for integrated models of biomolecular interaction networks. Genome Res. 13, 2498–2504. 10.1101/gr.1239303 14597658 PMC403769

[B59] Shevchenko A. Wilm M. Vorm O. Mann M. (1996). Mass spectrometric sequencing of proteins from silver-stained polyacrylamide gels. Anal. Chem. 68, 850–858. 10.1021/ac950914h 8779443

[B60] Singh A. Weissenböck H. Hess M. (2008). *Histomonas meleagridis*: immunohistochemical localization of parasitic cells in formalin-fixed, paraffin-embedded tissue sections of experimentally infected turkeys demonstrates the wide spread of the parasite in its host. Exp. Parasitol. 118, 505–513. 10.1016/j.exppara.2007.11.004 18155698

[B61] Sitek B. Scheibe B. Jung K. Schramm A. Stühler K. (2006). Difference Gel Electrophoresis (DIGE): the next generation of two-dimensional gel electrophoresis for clinical research in Methods and Principles in Medicinal Chemistry: Proteomics in Drug Research, eds Hamacher M. Marcus K. Stühler K. van Hall A. Warscheid B. Meyer H. (Weinheim: Wiley-VCH Verlag GmbH & Co. KGaA, Weinheim), 33–55.

[B62] Sulejmanovic T. Liebhart D. Hess M. (2013). *In vitro* attenuated *Histomonas meleagridis* does not revert to virulence, following serial *in vivo* passages in turkeys or chickens. Vaccine 31, 5443–5450. 10.1016/j.vaccine.2013.08.098 24055087

[B63] Taylor G. K. Goodlett D. R. (2005). Rules governing protein identification by mass spectrometry. Rapid Commun. Mass Spectrom. 19, 3420–3420. 10.1002/rcm.2225 16252315

[B64] Tyzzer E. E. (1919). Developmental phases of the protozoon of “Blackhead” in turkeys. J. Med. Res. 40, 1–30.3. 19972476 PMC2104353

[B65] Ungermann C. Langosch D. (2005). Functions of SNAREs in intracellular membrane fusion and lipid bilayer mixing. J. Cell Sci. 118, 3819–3828. 10.1242/jcs.02561 16129880

[B66] Van Dellen K. L. Chatterjee A. Ratner D. M. Magnelli P. E. Cipollo J. F. Steffen M.. (2006). Unique posttranslational modifications of chitin-binding lectins of *Entamoeba invadens* cyst walls. Eukaryot. Cell 5, 836–848. 10.1128/EC.5.5.836-848.2006 16682461 PMC1459681

[B67] Vizcaíno J. A. Csordas A. Del-Toro N. Dianes J. A. Griss J. Lavidas I. (2016). 2016 update of the PRIDE database and its related tools. Nucleic Acids Res. 44, D447–D456. 10.1093/nar/gkw880 26527722 PMC4702828

[B68] Zaragatzki E. Hess M. Grabensteiner E. Abdel-Ghaffar F. Al-Rasheid K. A. S. Mehlhorn H. (2010). Light and transmission electron microscopic studies on the encystation of *Histomonas meleagridis*. Parasitol. Res. 106, 977–983. 10.1007/s00436-010-1777-2 20143091

