# Supplementary material for: An Alliance of Gel-Based and Gel-Free Proteomic Techniques Displays Substantial Insight Into the Proteome of a Virulent and an Attenuated Histomonas meleagridis Strain
Source: Front Cell Infect Microbiol. 2018 Nov 16;8:407. doi: 10.3389/fcimb.2018.00407 (PMC6250841; doi:10.3389/fcimb.2018.00407)
Supplement: Supplementary file 1 [file Table_1.DOCX]

**Table S1**| Sequence coverage (%), mass to charge (*m/z*) ratio values, peptide scores and peptide sequence information for identifications of protein spots with significant (*P* ˂0.05) upregulation in fluorescent gel images of the cultivated virulent (25) and attenuated (303) *Histomonas meleagridis* strain. The significant differential expression of protein spots was detected by the two-dimensional differential gel electrophoresis (2D-DIGE) experiment, designated as 2D-DIGE-α. The identified proteins were categorized according to their proposed functions. Within categories, the identifications were sorted according to their fold upregulation (from high to low).

| **Spot ID^1^** | **Protein identity- Species- Contig ID^2^;**  **Accession Nr.** | **Method** | **Gel**  **image^3^** | **Seq. cov(%)** | ***m/z* values** | **Peptide scores^4^** | **Peptide sequences** |
| --- | --- | --- | --- | --- | --- | --- | --- |
| **Cytoskeleton/ plasminogen (PLG)-binding: virulent *H. meleagridis*** | | | | | | | |
| 63527 | Actin- Contig2112; HAGI01002078 | MALDI-TOF/TOF | 25 | 9 | 1515.6924  2712.1724 | 85  161 | K.IWHHTFYNELR.V K.AATTSECDISYTLPDGNVITIANER.F + Deamidated (NQ) |
| 78829 | Actin- Contig2112; HAGI01002078 | MALDI-TOF/TOF | 25 | 12 | 1186.7487  1515.8124  2711.4397 | 26  74  179 | R.SVFPSIVGRPK.Y  K.IWHHTFYNELR.V  K.AATTSECDISYTLPDGNVITIANER.F |
| 63624 | Actin- Contig2112; HAGI01002078 | MALDI-TOF/TOF | 25 | 16 | 1456.7446  2283.2168  2412.3379  2711.4072 | 112  70  58  61 | R.DEYNEAGPGIVHR.K  K.DLYANIVLSGGTTMFEGLPER.M  R.KDLYANIVLSGGTTMFEGLPER.M + Deamidated (NQ)  K.AATTSECDISYTLPDGNVITIANER.F |
| 63646 | Actin- Contig2112; HAGI01002078 | MALDI-TOF/TOF | 25 | 15 | 1456.7001  2283.1360  2712.3274 | 66  54  80 | R.DEYNEAGPGIVHR.K  K.DLYANIVLSGGTTMFEGLPER.M  K.AATTSECDISYTLPDGNVITIANER.F + Deamidated (NQ) |
| 63512 | Actin- Contig2112; HAGI01002078 | MALDI-TOF/TOF | 25 | 15 | 1515.7971  2283.2061  2711.3682 | 54  61  128 | K.IWHHTFYNELR.V  K.DLYANIVLSGGTTMFEGLPER.M  K.AATTSECDISYTLPDGNVITIANER.F |
| **Carbohydrate metabolism: virulent *H. meleagridis*** | | | | | | | |
| 63324 | Pyruvate, phosphate dikinase (PPDK)- Contig1102; HAGI01001086 | LC-MS/MS | 25 | 8 | 682.8157  472.7809  587.798  552.8187 | 12  8  12  11 | LETSPEDIEGMK+ Oxidation(M)@11  SAQGILTVR  ELGAEGIGLCR+ Carbamidomethyl(C)@10  LAVTYPEIAK |
| 79153 | Phosphoenolpyruvate carboxykinase (PEPCK)- Contig1899; HAGI01001872 | MALDI-TOF/TOF | 25 | 5 | 1455.7462  1717.8126 | 55  108 | K.TNLAMLIPPEALR.N + Deamidated (NQ); Oxidation (M)  R.AINPENGFFGVAPGTAR.E |
| 63521 | Phosphoenolpyruvate carboxykinase (PEPCK)- Contig1899; HAGI01001872 | MALDI-TOF/TOF | 25 | 10 | 1295.7012  1371.8182  1556.9468  1717.9696  2156.2852 | 34  53  71  97  96 | K.FLWPGYGDNAR.V  K.VPAVFHVNWFR.K  K.GKVPAVFHVNWFR.K  R.AINPENGFFGVAPGTAR.E  K.VTTVGDDIAWIKPDATGQLR.A |
| 63471 | Phosphoenolpyruvate carboxykinase (PEPCK)- Contig1899; HAGI01001872 | MALDI-TOF/TOF | 25 | 10 | 1295.6135  1371.7214  1556.8386  1717.8433  2156.1084 | 37  51  67  60  115 | K.FLWPGYGDNAR.V  K.VPAVFHVNWFR.K  K.GKVPAVFHVNWFR.K  R.AINPENGFFGVAPGTAR.E  K.VTTVGDDIAWIKPDATGQLR.A |
| 101054 | Phosphoenolpyruvate carboxykinase (PEPCK)- Contig1899; HAGI01001872 | MALDI-TOF/TOF | 25 | 4 | 1296.6632  1718.8658 | 29  78 | K.FLWPGYGDNAR.V + Deamidated (NQ)  R.AINPENGFFGVAPGTAR.E + Deamidated (NQ) |
| **Carbohydrate metabolism/ plasminogen (PLG)-binding: virulent *H. meleagridis*** | | | | | | | |
| 63536 | Glyceraldehyde-3-phosphate dehydrogenase (GAPDH)- Contig449; HAGI01000443 | MALDI-TOF/TOF | 25 | 9 | 1633.9249  1928.0492 | 83  24 | K.ELGVDVVLESTGIFR.T  R.AACMNIIPTSTGAAIALPR.V |
| 63529 | Glyceraldehyde-3-phosphate dehydrogenase (GAPDH)- Contig449; HAGI01000443 | MALDI-TOF/TOF | 25 | 18 | 1633.9778  1908.8999  1928.1038  2257.2751 | 98  72  71  105 | K.ELGVDVVLESTGIFR.T  K.VLSWYDNEWMYSCR.C  R.AACMNIIPTSTGAAIALPR.V  R.LLYPNEVQVVAIHDLCDMK.T |
| 63528 | Glyceraldehyde-3-phosphate dehydrogenase (GAPDH)- Contig449; HAGI01000443 | MALDI-TOF/TOF | 25 | 13 | 1633.9019  1908.8190  1928.0411 | 136  66  67 | K.ELGVDVVLESTGIFR.T  K.VLSWYDNEWMYSCR.C  R.AACMNIIPTSTGAAIALPR.V |
| 63530 | Glyceraldehyde-3-phosphate dehydrogenase (GAPDH)- Contig449; HAGI01000443 | MALDI-TOF/TOF | 25 | 13 | 1633.8425  1908.7495  1927.9496 | 141  67  96 | K.ELGVDVVLESTGIFR.T  K.VLSWYDNEWMYSCR.C  R.AACMNIIPTSTGAAIALPR.V |
| **Adaptation to stress: virulent *H. meleagridis*** | | | | | | | |
| 219386 | 14-3-3 protein- Contig1890; HAGI01001863 | LC-MS/MS | 25 | 34 | 462.2349  467.5666  489.7661  490.2585  490.2585  553.8115  554.3055  554.3055  551.8211  487.7751  712.8588  713.3513  576.8219  577.3141  464.748  513.5884  513.9175  670.3143  670.8054  491.7834  500.2951  500.788  500.788 | 14  15  13  13  10  13  12  12  8  13  20  21  16  11  10  18  10  18  11  13  11  11  11 | MIAAIIDHEESR  MIAAIIDHEESR+ Oxidation(M)@1  VEQLQAYK  VEQLQAYK+ Deamidated(Q)@5  VEQLQAYK+ Deamidated(Q)@3  VEQLQAYKK  VEQLQAYKK+ Deamidated(Q)@5  VEQLQAYKK+ Deamidated(Q)@3  KTILEELEK  TILEELEK  YCSEVIALVDQK+ Carbamidomethyl(C)@2  YCSEVIALVDQK+ Carbamidomethyl(C)@2; Deamidated(Q)@11  LLPAANTPEAR  LLPAANTPEAR+ Deamidated(N)@6  LKADYYR  AKECYENALEIAK+ Carbamidomethyl(C)@4  AKECYENALEIAK+ Carbamidomethyl(C)@4; Deamidated(N)@7  ECYENALEIAK+ Carbamidomethyl(C)@2  ECYENALEIAK+ Carbamidomethyl(C)@2; Deamidated(N)@5  QVAIELAQK+ Gln->pyro-Glu@N-term  QVAIELAQK  QVAIELAQK+ Deamidated(Q)@8  QVAIELAQK+ Deamidated(Q)@1 |
| 84570 | Cytosolic heat shock protein 70 (Cytosolic Hsp70)- Contig764; HAGI01000755 | MALDI-TOF/TOF | 25 | 6 | 1285.6268  1498.7172  1911.0029 | 57  74  145 | K.NALEGYCFGVR.N  R.AMFENLNDELFR.S  R.DNNLLGTFDLTGIPPAPR.G |
| **Peptidase activity: virulent *H. meleagridis*** | | | | | | | |
| 63659 | Clan CD, family C13, asparaginyl endopeptidase-like cysteine peptidase (Peptidase C13)- Contig42; HAGI01000042 | MALDI-TOF/TOF | 25 | 12 | 1671.8054  1857.9333  2014.9331 | 60  115  108 | R.YAVLLAGSNGWSNYR.H  R.HQADICTIYQLLINR.G  K.GQIFHTSAHENVYPGTEK.M |
| **Intracellular vesicle trafficking: virulent *H. meleagridis*** | | | | | | | |
| 63717 | Rab family GTPase (Rab11c-like)- Contig1980; HAGI01001951 | LC-MS/MS | 25 | 36 | 529.3161  526.785  637.8106  472.745  809.9093  446.7138  611.8185  690.3625 | 13  14  14  10  17  9  20  16 | IVLIGDSGVGK  STIGVEFATK  AQIWDTAGQER  AITSAYYR  ENADSNIVVMLVGNK+ Oxidation(M)@10  CDLSELR+ Carbamidomethyl(C)@1  AVTTEEGIGFAK  SENLLFIETSAR |
| **Unknown function: virulent *H. meleagridis*** | | | | | | | |
| 63318 | Hypothetical protein- Contig1489; HAGI01001468 | LC-MS/MS | 25 | 31 | 549.2859  449.7531  567.7998  515.7662  516.2598  656.7847  776.4218  440.2118  422.9659  684.2928  761.3212  402.7207 | 10  13  12  13  13  15  24  14  14  19  12  9 | LYYDWIPK  SHLATIEK  FAQWIIDNK  GAQNLGLSDR  GAQNLGLSDR+ Deamidated(Q)@3  WQDTFMPDTR+ Oxidation(M)@6  ADSSSLSVIFTGLVR  AQSHLNFCVDK+ Carbamidomethyl(C)@8  AQSHLNFCVDKLEK+Carbamidomethyl(C)@8  YEGDPYFFDSK  WNPCGEGTSETQR+ Carbamidomethyl(C)@4  SILETLE |
| **Metabolic processes: virulent *H. meleagridis*** | | | | | | | |
| 63664 | Phosphomanomutase (PMM)- Contig1867; HAGI01001842 | MALDI-TOF/TOF | 25 | 15 | 1788.0352  1659.9385  1849.9250  962.5387 | 89  86  34  34 | R.KILVLFDIDGTLTPSR.L  K.ILVLFDIDGTLTPSR.L  K.IGETSIRDQFTPAQMR.K  K.FIDWLLR.Y |
| **Adaptation to stress: bacterial** | | | | | | | |
| 63323 | Chaperone protein ClpB- *Escherichia coli*- J7R7G1_ECOLX | LC-MS/MS | 25 | 26 | 505.7621  647.3608  529.3033  584.8322  585.3225  672.8831  574.2719  879.9872  687.3671  492.9054  503.2815  585.8042  594.3148  643.8511  458.2562  828.4391  766.3875  458.9015  825.4589 | 9  14  13  12  7  17  16  16  14  13  11  11  12  18  8  20  20  12  13 | YTIDLTER  NNPVLIGEPGVGK  TAIVEGLAQR  IINGEVPEGLK  IINGEVPEGLK+ Deamidated(N)@3  VLALDMGALVAGAK+ Oxidation(M)@6  GELHCVGATTLDEYR+ Carbamidomethyl(C)@5  VFVAEPSVEDTIAILR  AIDLIDEAASSIR  MQIDSKPEELDR+ Oxidation(M)@1  ASLSGTQTIK  QLEAATQLEGK+ Gln->pyro-Glu@N-term  QLEAATQLEGK  VTDAEIAEVLAR  WTGIPVSR  VIGQNEAVDAVSNAIR  LVGAPPGYVGYEEGGYLTEAVR  GYEIHISDEALK  AIQQQIENPLAQQILSGELVPGK |
| **Metabolic processes: bacterial** | | | | | | | |
| 105883 | Glycerophosphodiester phosphodiesterase- *E. coli* - A0A024L1F7_ECOLX | MALDI-TOF/TOF | 25 | 11 | 1083.6490  1656.8032  2301.1733 | 35  90  111 | K.LVVHPYTVR.S  K.VYLQCFDADELKR.I  R.VHTFEEEIEFVQGLNHSTGK.N |
| **Carbohydrate up-take/ adaptation to stress: bacterial** | | | | | | | |
| 106853 | Maltose ABC transporter substrate-binding protein MalE- *E. coli*- A0A023LBH2_ECOLX | MALDI-TOF/TOF | 25 | 6 | 1267.6814  1767.9288 | 46  58 | K.LYPFTWDAVR.Y  R.FGGYAQSGLLAEITPDK.A |
|  | | | | | | | |
| **Cell division: attenuated *H. meleagridis*** | | | | | | | |
| 63266 | Cell division cycle protein 48-like (Cdc48-like)- Contig1011; HAGI01000996 | MALDI-TOF/TOF | 303 | 14 | 936.5075  1603.9354  1924.0336  1506.7323  1683.9628  1752.9958  2170.1040  2305.3276 | 53  119  54  89  109  110  145  122 | R.FPELFQR.F  R.HPQLFSNLGIKPPR.G  K.ETVEYPLRFPELFQR.F  R.QEHFNYALQNSR.K  R.VINQLLTELDGLEAR.K  R.LDQLIYIPLPDEPAR.M  R.QASPCIIFFDELDSITSAR.G  R.EIDIGVPDETGRLEILGIHTK.K |
| 63274 | Cell division cycle protein 48-like (Cdc48-like)- Contig1011; HAGI01000996 | MALDI-TOF/TOF | 303 | 12 | 1603.9993  1684.0128  1753.0728  1924.1255  2170.1941  2305.4485 | 93  87  78  26  100  56 | R.HPQLFSNLGIKPPR.G  R.VINQLLTELDGLEAR.K  R.LDQLIYIPLPDEPAR.M  K.ETVEYPLRFPELFQR.F  R.QASPCIIFFDELDSITSAR.G  R.EIDIGVPDETGRLEILGIHTK.K |
| **Carbohydrate metabolism/ plasminogen (PLG)-binding: attenuated *H. meleagridis*** | | | | | | | |
| 63468 | Enolase family protein- Contig1151; HAGI01001135 | MALDI-TOF/TOF | 303 | 19 | 1246.7542  1552.9054  2130.1465  2403.2727  2665.3955 | 66  81  98  98  104 | K.APPATIDHLVGR.E  K.LILDAGQNVMVSHR.S  K.LQEFMASPAPGIPYPDQLR.M  R.GNPTVETDVYVNYLGTVMFAGR.S  K.NLGDEGGFAPCLETPEEAITFIER.A |
| 63509 | Glyceraldehyde-3-phosphate dehydrogenase (GAPDH)- Contig449; HAGI01000443 | MALDI-TOF/TOF | 303 | 25 | 1633.9114  1908.8223  1928.0321  2257.2097  2525.2200 | 145  84  107  132  107 | K.ELGVDVVLESTGIFR.T  K.VLSWYDNEWMYSCR.C  R.AACMNIIPTSTGAAIALPR.V  R.LLYPNEVQVVAIHDLCDMK.T  K.NGFMTTVHSYTNDQVVTDVMHK.D |
| **Other** | | | | | | | |
| 63347 | Serum albumin precursor- *Bos taurus*- ALBU_BOVIN | MALDI-TOF/TOF | 303 | 10 | 927.5039  1439.8446  1479.8002  1567.7584  2045.0374 | 20  24  82  85  63 | K.YLYEIAR.R  R.RHPEYAVSVLLR.L  K.LGEYGFQNALIVR.Y  K.DAFLGSFLYEYSR.R  R.RHPYFYAPELLYYANK.Y |
| **Metabolic processes: attenuated *H. meleagridis*** | | | | | | | |
| 63369 | Clan MH, family M20, peptidase T-like metallopeptidase- Contig1068; HAGI01001053 | MALDI-TOF/TOF | 303 | 14 | 1065.5981  1482.7792  1731.9618  2340.2629  2526.2925 | 72  59  61  88  145 | R.FPSLFVAER.A  R.EHIWCPIAVMAR.F  R.VIFLDGHSDTVYPLR.D  K.IVSIATIAEEDNDGGAPMHIMR.K  R.IPADYYEEDKLCGTSNHETQR.L |
| **Cytoskeleton organization: attenuated** ***H. meleagridis*** | | | | | | | |
| 63416 | Coronin- Contig2106; HAGI01002072 | MALDI-TOF/TOF | 303 | 6 | 947.4982  1245.7933  1428.7698 | 55  54  90 | K.IHIYDMR.A  K.ALLHVPMIVPR.R  K.LHYIFTTGFSSR.A |

Spots of interest were excised and pooled from all four silver-stained fluorescent gels included in the 2D-DIGE-α experiment.

Proteins that take part in metabolic processes other than carbohydrate metabolism were grouped together.

**^1^** Spot ID = a unique number assigned to each protein spot by Delta2D software version 4.7 (Decodon GmbH, Greifswald, Germany).

**^2^** Contig identification number (ID) was obtained from the *de novo* transcriptome sequencing of a virulent and an attenuated *H. meleagridis* strain (Mazumdar et al.,2017).

**^3^** Gel image = 25: the protein spot was significantly upregulated in fluorescent gel images displaying proteins of the cultivated virulent strain.

303: the protein spot was significantly upregulated in fluorescent gel images displaying proteins of the cultivated attenuated strain.

**^4^** Peptide scores = identifications having at least one peptide with a MASCOT score ˃20 were regarded as statistical significant (*P* ˂0.05). The sum of peptide scores for each identification produced the protein score. Identifications with protein score ˃80 were regarded as significant in MASCOT and fulfil the stricter criteria of ProteinScape 2.1 software.

The following protein spots were not identified: 63535, 63735, 71550.
