# Supplementary material for: An Alliance of Gel-Based and Gel-Free Proteomic Techniques Displays Substantial Insight Into the Proteome of a Virulent and an Attenuated Histomonas meleagridis Strain
Source: Front Cell Infect Microbiol. 2018 Nov 16;8:407. doi: 10.3389/fcimb.2018.00407 (PMC6250841; doi:10.3389/fcimb.2018.00407)
Supplement: Supplementary file 2 [file Table_2.DOCX]

**Table S2|** Sequence coverage (%), mass to charge (*m/z*) ratio values, peptide scores and peptide sequence information for identifications of protein spots with significant (*P* ˂0.05) upregulation in fluorescent gel images of the cultivated virulent (25) and attenuated (303) *Histomonas meleagridis* strain. The significant differential expression of protein spots was detected by the two-dimensional differential gel electrophoresis (2D-DIGE) experiment, designated 2D-DIGE-β. The identified proteins were categorized according to their proposed functions. Within categories, the identifications were sorted according to their fold upregulation (from high to low).

| **Spot ID^1^** | **Protein identity- Species- Contig ID^2^; Accession Nr.** | **Method** | **Gel**  **image^3^** | **Seq. cov(%)** | ***m/z* values** | **Peptide scores^4^** | **Peptide sequences** |
| --- | --- | --- | --- | --- | --- | --- | --- |
| **Carbohydrate metabolism/ plasminogen (PLG)-binding: virulent *H. meleagridis*** | | | | | | | |
| 610 | Fructose-bisphosphate aldolase (FBAL)- Contig601; HAGI01000595 | MALDI-TOF/TOF | 25 | 21 | 1140.7212  1440.8101  1609.8848  1765.9984  2106.2837  2173.1873 | 46  79  50  26  100  84 | K.FPPGTKPQIR.L  K.MPDAVGIPEDQLR.E  R.LFHEHPEWFDPR.Q  R.RLFHEHPEWFDPR.Q  K.LIPIVLHLDHGDSFELCK.S  K.AEHHTYTRPEEVQDFVSK.T |
| 526 | Glyceraldehyde-3-phosphate dehydrogenase (GAPDH)- Contig449; HAGI01000443 | MALDI-TOF/TOF | 25 | 18 | 1633.9984  1908.9381  1928.1401  2257.3411 | 136  52  96  87 | K.ELGVDVVLESTGIFR.T  K.VLSWYDNEWMYSCR.C  R.AACMNIIPTSTGAAIALPR.V  R.LLYPNEVQVVAIHDLCDMK.T |
| 509 | Glyceraldehyde-3-phosphate dehydrogenase (GAPDH)- Contig449; HAGI01000443 | MALDI-TOF/TOF | 25 | 16 | 918.5209  1634.0336  1928.1810  2257.3691 | 28  157  135  125 | R.CADIFHR.L *  K.ELGVDVVLESTGIFR.T  R.AACMNIIPTSTGAAIALPR.V  R.LLYPNEVQVVAIHDLCDMK.T * |
| 524 | Glyceraldehyde-3-phosphate dehydrogenase (GAPDH)- Contig449; HAGI01000443 | MALDI-TOF/TOF | 25 | 15 | 918.4788  1633.9781  1908.9302  1928.1149 | 24  140  42  112 | R.CADIFHR.L  K.ELGVDVVLESTGIFR.T  K.VLSWYDNEWMYSCR.C  R.AACMNIIPTSTGAAIALPR.V |
| **Cytoskeleton/ plasminogen (PLG)-binding: virulent *H. meleagridis*** | | | | | | | |
| 535 | Actin- Contig2112; HAGI01002078 | MALDI-TOF/TOF | 25 | 9 | 1515.9373  2711.6079 | 48  76 | K.IWHHTFYNELR.V *  K.AATTSECDISYTLPDGNVITIANER.F * |
| 627 | Actin- Contig2112; HAGI01002078 | MALDI-TOF/TOF | 25 | 9 | 1456.7965  2283.2666 | 79  90 | R.DEYNEAGPGIVHR.K  K.DLYANIVLSGGTTMFEGLPER.M |
| 386163 | Actin family protein- Contig444; HAGI01000438 | MALDI-TOF/TOF | 25 | 9 | 1711.9  2403.4 | 43  85 | K.ETTTYVVPYTLPDGR.V  K.INCSEHPVLVTEAPLNPLENR.K |
| **Carbohydrate metabolism: virulent *H. meleagridis*** | | | | | | | |
| 202345 | Phosphoenolpyruvate carboxykinase (PEPCK)- Contig1899; HAGI01001872 | MALDI-TOF/TOF | 25 | 12 | 1295.6348  1438.8215  1556.8765  1717.8975  2156.1785 | 65  58  66  127  99 | K.FLWPGYGDNAR.V  K.TNLAMLIPPEALR.N  K.GKVPAVFHVNWFR.K  R.AINPENGFFGVAPGTAR.E  K.VTTVGDDIAWIKPDATGQLR.A |
| 63267 | Iron-containing alcohol dehydrogenase (ADH)- Contig362; HAGI01000356 | LC-MS/MS | 25 | 40 | 838.0445  843.3755  843.3774  896.4388  1019.867  490.3007  667.0297  779.3858  787.8992  788.384  795.8973  576.774  595.6245  625.6402  625.9678  625.968  555.3218 | 19  20  15  19  11  12  19  15  16  8  13  10  11  22  12  14  10 | EAGMEVETFEGVEADPSVETVMR  EAGMEVETFEGVEADPSVETVMR+ Oxidation(M)@4  EAGMEVETFEGVEADPSVETVMR+ Oxidation(M)@22  FCAIPSTSGTATEVTAFAVITDYHK+ Carbamidomethyl(C)@2  GIKYPLADFNITPDVAIVDPELAETMPK  LIHENLIK  FIHLPGSTTEELVDSLIK  QMNVELEIPTCIK+ Gln->pyro-Glu@N-term; Carbamidomethyl(C)@11  QMNVELEIPTCIK+ Carbamidomethyl(C)@11  QMNVELEIPTCIK+ Deamidated(N)@3; Carbamidomethyl(C)@11  QMNVELEIPTCIK+ Oxidation(M)@2; Carbamidomethyl(C)@11  EYEGGIIDEK  EYEGGIIDEKEFLDK  LSAVATNAIGDACTGSNPR+ Carbamidomethyl(C)@13  LSAVATNAIGDACTGSNPR+ Deamidated(N)@7; Carbamidomethyl(C)@13  LSAVATNAIGDACTGSNPR+ Carbamidomethyl(C)@13; Deamidated(N)@17  LLLAVFYDR |
| 640 | Phosphoglycerate mutase (PGlyM)- Contig1739; HAGI01001716 | MALDI-TOF/TOF | 25 | 16 | 904.5896  1150.7128  1369.6654  1708.9353 | 36  87  93  78 | K.YQIVLLR.H  K.VLIAAHGNSLR.A  R.HGESQWNLENR.F  R.VMPFWHDQIVPAIR.S |
| **Metabolic processes: virulent *H. meleagridis*** | | | | | | | |
| *42635* | *Clan MG, family M24, aminopeptidase P-like metallopeptidase- Contig29;* *HAGI01000029* | *MALDI-TOF/TOF* | *25* | *3* | *1887.9719* | *101* | *R.YACDVNCNAFHDVFR.K* |
| 356639 | Clan MG, family M24, aminopeptidase P-like metallopeptidase- Contig29; HAGI01000029 | LC-MS/MS | 25 | 49 | 658.8744  621.3241  532.2864  543.7932  714.3909  538.2844  624.3527  409.7439 | 14  11  9  14  13  13  17  11 | YQVDEVLLLPK  IYTLEPTYNK  DVTIPLDYK  VALEAIGEER  QIKSEDELTLIR+ Gln->pyro-Glu@N-term  SEDELTLIR  LVYQAVLDAQK  VMAAGLIK+ Oxidation(M)@2 |
| **Adaptation to stress: bacterial** | | | | | | | |
| 417 | 60 kDa chaperonin- *Escherichia coli*- Q6Q099_ECOLX | LC-MS/MS | 25 | 38 | 493.2866  461.2556  801.4189  609.304  617.3007  617.3007  529.8081  532.2593  880.9557  445.7359  600.3375  608.3379  523.2792  615.9775  847.8784  571.2635  414.271  517.7621  784.4443  647.3311  653.0081 | 14  12  27  12  11  11  16  9  23  9  11  9  14  22  18  10  13  12  26  10  16 | GVNVLADAVK  SFGAPTITK  ANDAAGDGTTTATVLAQAIITEGLK  AVAAGMNPMDLK  AVAAGMNPMDLK+ Oxidation(M)@9  AVAAGMNPMDLK+ Oxidation(M)@6  AVTVAVEELK  ALSVPCSDSK+ Carbamidomethyl(C)@6  AIAQVGTISANSDETVGK  LIAEAMDK  EMLPVLEAVAK  EMLPVLEAVAK+ Oxidation(M)@2  ATLEDLGQAK  DTTTIIDGVGEEAAIQGR  QQIEEATSDYDREK+ Gln->pyro-Glu@N-term  QQIEEATSDYDREK  LAGGVAVIK  VGAATEVEMK  AAVEEGVVAGGGVALIR  QIVLNCGEEPSVVANTVK+ Gln->pyro-Glu@N-term; Carbamidomethyl(C)@6  QIVLNCGEEPSVVANTVK+ Carbamidomethyl(C)@6 |
| **Carbohydrate metabolism: bacterial** | | | | | | | |
| 197187 | Class II fructose-bisphosphate aldolase - *E. coli* - A0A0D8VY27_ECOLX | MALDI-TOF/TOF | 25 | 10 | 1878.0808  1991.1848 | 120  100 | K.IFDFVKPGVITGDDVQK.V  K.VKAPVIVQFSNGGASFIAGK.G |
|  | | | | | | | |
| **Cytoskeleton organization: attenuated *H. meleagridis*** | | | | | | | |
| 408 | Coronin- Contig2106; HAGI01002072 | MALDI-TOF/TOF | 303 | 14 | 947.5921  1245.9027  1428.9180  1854.2557  2121.2522 | 53  71  104  132  165 | K.IHIYDMR.A *  K.ALLHVPMIVPR.R *  K.LHYIFTTGFSSR.A *  K.IKPNHNQPLATLTGHGR.K *  K.GATNNQNFLDIAWSQDGNR.V * |
| 409 | Coronin- Contig1193; HAGI01001177 | MALDI-TOF/TOF | 303 | 24 | 947.6010  1275.9348  1414.9156  1859.1752  2025.3481  2180.2954  2779.9714 | 55  73  105  108  98  134  143 | K.IHIYDMR.A *  K.SLIHIPMIVPR.R *  K.LHYIFSTGFSSR.A *  K.HPIMALSSFQMQEALR.C *  K.GNIKPNHNQPLATLTGHGR.K *  K.GNTNNQNFLDISWSQDGNR.V *  K.APVIDVAFNPFADNIFVSASEDATIR.V * |
| **Metabolic processes: attenuated *H. meleagridis*** | | | | | | | |
| 587944-1 | Transketolase family protein- Contig1077; HAGI01001062 | MALDI-TOF/TOF | 303 | 4 | 897.5059  1625.8162  1642.8497 | 43  75  113 | R.YLEFGIR.E  R.QNAPPIEGASFDGALR.G + Gln->pyro-Glu (N-term Q)  R.QNAPPIEGASFDGALR.G |
| 587944-2 | Transketolase family protein- Contig1077; HAGI01001062 | MALDI-TOF/TOF | 303-Gel4 | 6 | 897.4951  1287.7104  1625.7910  1642.8192 | 36  36  38  136 | R.YLEFGIR.E  K.THPVPELIERP.-  R.QNAPPIEGASFDGALR.G  R.QNAPPIEGASFDGALR.G |
| **Carbohydrate metabolism/ plasminogen (PLG)-binding: attenuated *H. meleagridis*** | | | | | | | |
| 495 | Glyceraldehyde-3-phosphate dehydrogenase (GAPDH)- Contig449; HAGI01000443 | MALDI-TOF/TOF | 303 | 18 | 1633.9811  1908.9390  1928.1563  2257.3005 | 149  72  138  96 | K.ELGVDVVLESTGIFR.T  K.VLSWYDNEWMYSCR.C  R.AACMNIIPTSTGAAIALPR.V  R.LLYPNEVQVVAIHDLCDMK.T |
| **Metabolic processes: bacterial** | | | | | | | |
| 153815 | Bifunctional purine biosynthesis protein purH- *E. coli*- A0A085PAJ0_ECOLX | MALDI-TOF/TOF | 303 | 19 | 1332.7  1419.6  1566.8  2042.9  2201.0  2262.0 | 96  42  114  123  53  74 | K.AGIVEFAQALSAR.G  K.GSSMASDAFFPFR.D  K.TDPTSAFGGIIAFNR.E  K.HANPCGVAIGNSILDAYDR.A  K.GLPVTEVSDYTGFPEMMDGR.V  R.EGCSLEDAVENIDIGGPTMVR.S |

Spots of interest were excised and pooled from all four silver-stained fluorescent gels included in the 2D-DIGE-β experiment.

Proteins that take part in metabolic processes other than carbohydrate metabolism were grouped together.

**^1^** Spot ID = a unique number assigned to each protein spot by Delta2D software version 4.7 (Decodon GmbH, Greifswald, Germany).

**^2^** Contig identification number (ID) was obtained from the *de novo* transcriptome sequencing of a virulent and an attenuated *H. meleagridis* strain (Mazumdar et al., 2017).

**^3^** Gel image = 25: the protein spot was significantly upregulated in fluorescent gel images displaying proteins of the cultivated virulent strain.

303: the protein spot was significantly upregulated in fluorescent gel images displaying proteins of the cultivated attenuated strain.

**^4^** Peptide scores = identifications having at least one peptide with a MASCOT score ˃20 were regarded as statistical significant (*P* ˂0.05). The sum of peptide scores for each identification produced the protein score. Identifications with protein score ˃80 were regarded as significant in MASCOT and fulfil the stricter criteria of ProteinScape 2.1 software.

* Indicates peptides found with 150 ppm mass tolerance:

Identification in grey/italics: protein spots identified with just one peptide.

The following protein spots were not identified: 457(gel image: 25) and 63593 (gel image: 25).
