# Supplementary material for: An Alliance of Gel-Based and Gel-Free Proteomic Techniques Displays Substantial Insight Into the Proteome of a Virulent and an Attenuated Histomonas meleagridis Strain
Source: Front Cell Infect Microbiol. 2018 Nov 16;8:407. doi: 10.3389/fcimb.2018.00407 (PMC6250841; doi:10.3389/fcimb.2018.00407)
Supplement: Supplementary file 3 [file Table_3.docx]

**Table S3|** Mass to charge (*m/z*) ratio values and peptide sequence information for protein identifications with significant upregulation in protein samples of the cultivated virulent (25) and attenuated (303) *Histomonas meleagridis* strain. The significant differential expression of proteins was detected by SWATH MS. The identified proteins were categorized according to their proposed functions. Within categories, the identifications were sorted according to their fold upregulation (from high to low).

| **Protein Nr.^1^** | **Protein identity- Contig ID^2^; Accession Nr.** | **Protein**  **sample^3^** | ***m/z* values** | **Peptide sequences** |
| --- | --- | --- | --- | --- |
| **Peptidase activity** | | | | |
| 220 | Clan CA, family C1, cathepsin L-like cysteine peptidase (Cathepsin L-like)- Contig2131; HAGI01002930 | 25 | 737.3464  975.0863  772.8905  1026.1274  813.8684  963.4396 | SYEHGIEGDEDYLAELLTR  DQGQC[CAM]GSC[CAM]WAFGTVQAC[CAM]ESAYALATGK  NNQC[CAM]GVASDALLVLA  C[CAM]SSYNLNHAVGC[CAM]VGYGTDNGVDYWIVR  NSWGTSWGENGYIR  GVC[CAM]DIAIDASSYSFQLYTSGVYNDSR |
| 124 | Clan CA, family C1, cathepsin L-like cysteine peptidase (Cathepsin L-like)- Contig2161; HAGI01002235 | 25 | 777.7007  948.4399  1127.1893  588.7822  952.4347  856.3825 | AVGSISDYLEVASYDEDDLAAK  C[CAM]SSTSLDHGVAAVGYGSDGATNYWIVR  VTNNGPTC[CAM]IAIDASHYSFQLYSSGIYDETR  FAAYTPSEYK  LLSFSEQNLVDC[CAM]VTTC[CAM]YGC[CAM]NGGLMTSAYDYVIR  DQASC[CAM]GSC[CAM]WAFSAIAGAEGAYFLK |
| 512 | LysM peptidoglycan domain-containing protein (LysM)- Contig1659; HAGI01001637 | 25 | 653.8173  623.291  757.6952  954.4511 | SDLGNIYAGDGPK  ELASGSAYEYR  FGWVDTSSTVINDLNSC[CAM]LSR  NGMNTLC[CAM]ANGATVEQVTK |
| 229 | Clan CD, family C13, asparaginyl endopeptidase-like cysteine peptidase (Peptidase C13)- Contig00252_LP; HAGI01002770 | 25 | 504.4959  619.9929  496.548  791.3401  609.6481  1201.2084 | GQIFHTSAHENVYPGTEK  HQADIC[CAM]TIYQLLINR  GSHVMFYGDESMK  GFTDDEITMMQYDDIATYSR  RYAVLLAGSNGWSNYR  YC[CAM]LFGIEAC[CAM]YAGSLAQEFTAPNMC[CAM]TITAANEK |
| 507 | Basic secretory protein (BSP) family protein - Contig857; HAGI01000846 | 25 | 555.3011  1028.146  1068.1693  584.2968  688.8046 | ATFTTTVNVR  ASPSTSSAVVAQYSAGQSVTYDSIYSNEGR  YC[CAM]C[CAM]AIDTNGAQYITVGGGGSPSPSLPYLQR  WDAYVLIGSQDLSQR  EIYNSAGWGWGH |
| 289 | Clan CA, family C1, cathepsin L-like cysteine peptidase (Cathepsin L-like)- Contig1674; HAGI01001652 | 25 | 810.4169  1005.7434  595.79  433.2519  569.2516  619.7831 | LNHAVNAVGYGVQDGTPYWIVR  QGGQFMREDDYPYTGTDGSC[CAM]QWDSSK  FATYTPAEYK  GYILMIR  NSWGTSWGDK  FAAPDNFDWR |
| 323 | NlpC/P60 superfamily cysteine peptidase domain-containing protein (NlpC/P60)- Contig1788; HAGI01001764 | 25 | 726.8807  806.0667  719.8728  786.7194  846.4267  790.874 | SSGSTSATILTSVNK  VNGIIGYMHPDYVSISSSTTFK  SSGSTSASILTSVNK  VNGIIGYMHPDYVSISSGSSFK  VNGIIGYMHPDYVTI  VVDGTGGC[CAM]VTSAQMAK |
| 753 | NlpC/P60 superfamily cysteine peptidase domain-containing protein (NlpC/P60)- Contig04155_HP; HAGI01002646 | 25 | 435.2638  478.2878  854.7108  571.7593 | QVGISIPR  VNIGALNVR  YVYGATGPNTFDC[CAM]SGLTQWC[CAM]HK  FEDGEGYVAR |
| **Unknown function** | | | | |
| 907 | Hypothetical protein- Contig04355_LP; HAGI01003301 | 25 | 543.9664  873.9365 | YQSVTVKPPEGTVPK  EINQNVPQFSLESNK |
| 637 | Hypothetical protein- Contig02848_HP; HAGI01002296 | 25 | 797.4039  515.7877  1016.4187 | ITFDEIISGENEVK  NFGNIGPLAK  EEIEENIDYYDENGDGK |
| 242 | Hypothetical protein- Contig593; HAGI01000587 | 25 | 808.6744  538.7742  788.9403  779.3752  617.9492  622.3215 | IVQQIVDEVNQIQAPYGTDASVGLHGPGIGR  FTLEPSVFH  LGFISNESGWLLIK  IFTEPSDATFSTGGK  EEEAGNQHDFLMYLR  DYFIGFLSGPK |
| 872 | Hypothetical protein- Contig03282_HP; HAGI01002399 | 25 | 906.4072  901.3929 | AEIDYTPSQLTSC[CAM]PQGYTC[CAM]PSDVK  C[CAM]INNYYC[CAM]WPIGDAR |
| **Cytoskeleton organization** | | | | |
| 108 | α-Actinin- Contig328; HAGI01000323 | 25 | 1039.9995  548.9495  727.3698  594.8057  743.8748  567.3501 | AGVPVYLDPEDLYGNVDDK  FELAQPNRDDIAQK  FVFGDGDDITIVR  NAAIALQAC[CAM]EK  LTDELYLNSAGYK  LLYTQLAALK |
| 625 | Actin-binding protein (ABP)- Contig497; HAGI01000491 | 25 | 449.2117  464.5468  609.2664  549.3031 | YMDGGIDSGFRK  EIQGHESDAFMK  YMDGGIDSGFR  GNYIVIYAGK |
| **Metabolic processes** | | | | |
| 329 | F420-0-gamma-glutamyl ligase (CofE-AF)- Contig02484_HP; HAGI01002231 | 25 | 886.8094  674.6772  642.809  429.8693  651.6644  1127.2196 | IWELADPIVSPAHTSGLIGTPNEIK  RLTDLLGSLC[CAM]DLTTGSGDK  YIADNDFGELK  NVLC[CAM]C[CAM]DIHTR  NIEVMIYGDGAFKDPVGK  GAEIIYGLDDILNHSINGSGYNPEYGVLGSNK |
| **Calcium ion binding** | | | | |
| 406 | EF hand family protein- Contig2024; HAGI01001994 | 25 | 511.2595  787.3791  554.9154  437.2342  775.6817 | LVAETHFTEDEIK  FC[CAM]FGLFDIDGNGTIEREELK  LDHDDGVIDMEEFK  GLSAIC[CAM]PR  IFAAFDNDNSSTIDFEEYAR |
| **Lipid metabolic process/ Pore-forming proteins** | | | | |
| 984 | Surfactant protein B-like (SPB-like)- Contig03153_LP; HAGI01003015 | 25 | 638.7068  642.313 | TVVGGIAGFIGASPVQSIIK  EALAEYESLMK |
| **Protein synthesis** | | | | |
| 788 | 60S acidic ribosomal protein P1 (60S)- Contig03716_HP; HAGI01002520 | 25 | 534.7878  629.3253 | NHDITELVK  GTALGGAAPAAGASTGAAEEKK |
| **Carbohydrate metabolism** | | | | |
| 258 | Adenylate kinase family protein (ADK)- Contig04065_HP; HAGI01002611 | 25 | 585.3011  609.3481  843.922  686.3495  943.9712  801.4721 | FGYASAGDLLR  SVIFVLGGPGSGK  EGMLETINANQPIEK  NAIISSESQYFLLDGFPR  AAAC[CAM]LMLDAPDEVLIER  RIAEIINAGQLVPPELLVDTLK |
| **Membrane trafficking** | | | | |
| 716 | C2 domain-containing protein- Contig1639; HAGI01001617 | 25 | 648.3023  789.6459  909.9313 | SDPYC[CAM]VISVSSSNDTRR  VIENSLKPMWNEEFHFNIPNPSNSALK  DDDMAHLEVQLC[CAM]SLPVGQVVDQWYNMIPAHR |
| **Vesicle fusion** | | | | |
| 584 | SNARE domain-containing protein- Contig04118_HP; HAGI01002630 | 25 | 686.3776  589.3037  817.9047  406.5594 | LLQIQSNLDEAK  NAIELADFASSQLATSK  GEAAAIAVVSEDYPSR  RGIVQYQEPK |
| **Signaling** | | | | |
| 341 | G-protein α subunit (Gprotein α)- Contig1461; HAGI01001440 | 25 | 559.7974  780.6743  449.5456  889.9803  498.2652 | ILTLGAGEC[CAM]GK  AAYPDFTGDTSNADEC[CAM]IEHVK  ITAGEFDDHDVK  TTGISTIDFLIQDNIK  VNLIYDMK |
|  | | | | |
| **Carbohydrate metabolism** | | | | |
| 429 | Alcohol dehydrogenase iron-containing family protein (ADH)- Contig246; HAGI01000245 | 303 | 703.6893  584.295  515.2716  967.5045  549.6066  870.4138 | EYKPDLILSVGGGSC[CAM]LDGTK  TQVAFGTGC[CAM]VK  LEDGKDPWEILTK  LAGGNNNTYPVFSLLEPK  ADVTEALAALEC[CAM]EVR  ILC[CAM]TFGGGSIDLNGC[CAM]R |
| 156 | Phosphofructokinase family protein (PFK)- Contig1563; HAGI01001541 | 303 | 668.9136  589.8244  493.2531  808.9414  731.3796 | IQLVPLEQLVGK  IADAITNAYVK  LPEWTDPK  AVSGTLGLGGTWLGTAR  GLIEGQMFPLNSR |
| 113 | Iron hydrogenase (Fe-Hyd)- Contig1190; HAGI01001174 | 303 | 664.8714  585.3299  695.8566  593.324  675.8491  864.4805 | VGVAVAQGIANAMK  ELPGTDLVAVR  DLSTNSITIDPSK  VAVC[CAM]QTAPAIR  VFNVAIMPC[CAM]TAK  INLSDALGLPAGTISTGK |
| 119 | Iron hydrogenase 64 kDa (Fe-Hyd-64 kDa)- Contig837; HAGI01000826 | 303 | 720.9121  871.988  703.3227  686.8408  537.3011  624.8256 | LAEISGGQLNGLLR  IGLADAFGVPISSLPTGK  LLDVHDQQC[CAM]TSC[CAM]IAYER  GFSETDFSLTIR  SQVQEILTR  VASVQGMANIMK |
| 142 | Iron hydrogenase 64 kDa (Fe-Hyd-64 kDa)- Contig1051; HAGI01001036 | 303 | 704.6845  701.8842  582.3389  816.407  443.9224  624.6404 | VQEAVSANLEMLLASHDER  MLVQPTSGVTLEK  STITMLSGLLK  AKFPLYSSNC[CAM]PAFVNFIEQSK  VAQIDINGKPFK  FAAVDQLHEAQNLMER |
| 223 | NADH dehydrogenase 51kDa (NADH Dehyd 51 kDa)- Contig292; HAGI01000290 | 303 | 788.0919  863.1026  845.4296  1054.8287  1048.4891  537.2617 | GSALGTGAIIVLDKDVDLAGAYSR  GAGAYVC[CAM]GEETALLNSIEGLPGRPR  GLFGC[CAM]PTC[CAM]VNNVETISSVPTILR  EDLYDLEHTALATNNC[CAM]IC[CAM]ALAGASSDPIK  WSFVPQNENQPHYLIINADEGEPGTC[CAM]K  NLYGDEGADLESAKK |
| 538 | Phosphofructokinase family protein (PFK)- Contig570; HAGI01000564 | 303 | 489.612  852.4149  602.7958 | VVSVPLDEIDKPR  AGC[CAM]FLGTAKPQGILTDQTMDTAMK  LADYLSEHEK |
| 114 | Iron hydrogenase (Fe-Hyd)- Contig2186; HAGI01002119 | 303 | 615.3372  631.3321  695.3651  888.9907  627.3  825.432 | VTIC[CAM]QTAPAIR  AC[CAM]TNVAGQSILK  SVVNVAIMPC[CAM]TAK  ISLSDALGLPFGTISTGK  DSIEEMESIFKNPQGR  SPAGMLSSAIINDFVK |
| **Metabolic processes** | | | | |
| 171 | Xanthine dehydrogenase (XDH)- Contig708; HAGI01000700 | 303 | 756.7522  509.7612  918.712  633.0156  628.795  1024.5146 | AIGEPPLLLAGSC[CAM]AFAIIDAIR  DGTVLVTHGGVEMGQGLHTK  TINPAIDIGQLEGGFIQGYGLLTMEELIHGDNDK  MAQVAAETLRVPIENVR  MLSESDIGYNK  IPGFNDIPVEFNAHLMPGSGNPIGIYSSK |
| 673 | Amidohydrolase family protein- Contig243; HAGI01000242 | 303 | 902.4131  677.714  609.2991 | DLSHEIENDGAVVFEDDTIIEC[CAM]GK  GLALKDPPAYTFLEVLQR  NHIAYEVTDR |
| 402 | Xanthine dehydrogenase (XDH)- Contig19; HAGI01000019 | 303 | 717.7042  578.3138  684.0177  692.3379  710.364  929.4783 | IPGFNDLPGEFNAHLLPGSR  AILHLDSSYNIPVYK  DGTVLISHAGVEMGQGLHTK  STVYTNAC[CAM]AVAAR  DRGLNEWIPVNFPLTSDR  GLNEWIPVNFPLTSDR |
| 626 | Aminotransferase classes I and II family protein- Contig03239_HP; HAGI01002389 | 303 | 656.5961  999.8197 | LLEATGIVVVPGSGHGQVEGTYHFR  SINLSSNSVGQLLFSAYC[CAM]NPPKSPEC[CAM]K |
| 285 | Pyridoxal-phosphate dependent enzyme family protein (PyrdxlP-dep)- Contig334; HAGI01000329 | 303 | 883.4124  960.1298  691.3718  775.9071  803.446  504.9279 | GSTVVAVSGGNHSQGVALAC[CAM]TLC[CAM]DC[CAM]K  EEVIVTPLTFPSNLSHEC[CAM]GC[CAM]NLSFK  SIVYVPEFAAASK  VIGVQMASC[CAM]PVVYK  IITEGAGAASFAAVLSK  EHYSLTIDVPNPK |
| 129 | Dihydroorotate dehydrogenase family protein (DHOD)- Contig290; HAGI01000288 | 303 | 715.3553  746.876  641.8029  817.7263  759.3648  941.9066 | IPLVASMGYTAEDIAHC[CAM]APK  VAPFADAIELSTHYIADDPKPMQDAIR  DVYGQIAEEMK  EAGASALTC[CAM]VNSFGPC[CAM]LALDIER  TVSVGPAPVPQPNMMENQDHK  LNDDGIAEC[CAM]NGELC[CAM]FR |
| **Unknown function** | | | | |
| 365 | Hypothetical protein- Contig634; HAGI01000628 | 303 | 561.2972  664.8802  682.3224  584.6003  743.9274  907.9625 | VSLDSC[CAM]LISK  NNIGVNVIGLSTK  YGIEIDQDADPK  GGTGSIEEC[CAM]DITDNRK  GASIIISDSGIVNIK  AAIIVNDNGVLNAC[CAM]NTR |
| 505 | Hypothetical protein- Contig2180; HAGI01002115 | 303 | 468.2691  544.9399  807.063  461.2468  434.5739 | NSLLAYVR  GMIGVDITGDATAHFK  FAASMAQTYVSAALGSAVSQMATR  NVLGFMIQ  NHAIVHAANIGGK |
| **Cell division** | | | | |
| 879 | Chromosome partitioning protein ATPase- Contig1699; HAGI01001677 | 303 | 676.911  694.0598 | GPLVGGLIGDLLTK  IDGAILVTSPQEVAIADVIR |
| **Proteostasis** | | | | |
| 248 | Chaperonin CPN60 hydrogenosomal (CPN60-Hyd)- Contig1546; HAGI01001524 | 303 | 550.3397  657.3323  457.2527  860.9392  672.8345  575.3293 | GIAIAVDTIVK  VTTHDEIAQVATISANGDK  ALSAGLDPNEVRK  TNDLAGDGTTTATLLTR  NAMIEQPYGPPK  TIGTIIADAFK |
| **Cytoskeleton organization** | | | | |
| 67 | Coronin putative- Contig1058; HAGI01001043 | 303 | 860.9614  894.4185  962.1296  590.8025  637.828  507.8028 | GVAEPPSTYYISLPVK  LC[CAM]PTGLQDAVMNDFSSMIYSIYR  SIELDTSSGHMLPMYEEGSGLIYLGGK  SEEFLTPITEPITSFNHNRK  VFETLANPNGGR  QILIWDVK |
| **Adhesion/ Carbohydrate metabolism** | | | | |
| 152 | Adhesin protein AP-65/malic enzyme (AP-65/ME)- Contig2156; HAGI01002133 | 303 | 744.845  664.2831  591.3574  1018.5023  476.252  844.4601 | ALFC[CAM]SGSPFPDYK  FNPSGEYDHVY  SALLPPMALIR  YLYEQELATAEIPAHMTLAEFLESQR  NIIMFDAK  TLVPSQANNSWIFPAVGFALVATK |

Proteins that take part in metabolic processes other than carbohydrate metabolism are grouped together.

**^1^** Protein number assigned by sequential window acquisition of all theoretical mass spectra (SWATH) MS.

**^2^** Contig identification number (ID) was obtained from the *de novo* transcriptome sequencing of a virulent and an attenuated *H. meleagridis* strain (Mazumdar et al., 2017). HP = the extension was assigned to contigs specific to the attenuated strain. LP = the extension was assigned to contigs specific to the virulent strain.

**^3^** Protein sample = 25: the identified protein was significantly upregulated in protein samples of the cultivated virulent strain. 303: the identified proteins was significantly upregulated in protein samples of the cultivated attenuated strain.
