# Supplementary material for: An Alliance of Gel-Based and Gel-Free Proteomic Techniques Displays Substantial Insight Into the Proteome of a Virulent and an Attenuated Histomonas meleagridis Strain
Source: Front Cell Infect Microbiol. 2018 Nov 16;8:407. doi: 10.3389/fcimb.2018.00407 (PMC6250841; doi:10.3389/fcimb.2018.00407)
Supplement: Supplementary file 4 [file Presentation_1.PPTX]

## Slide 1
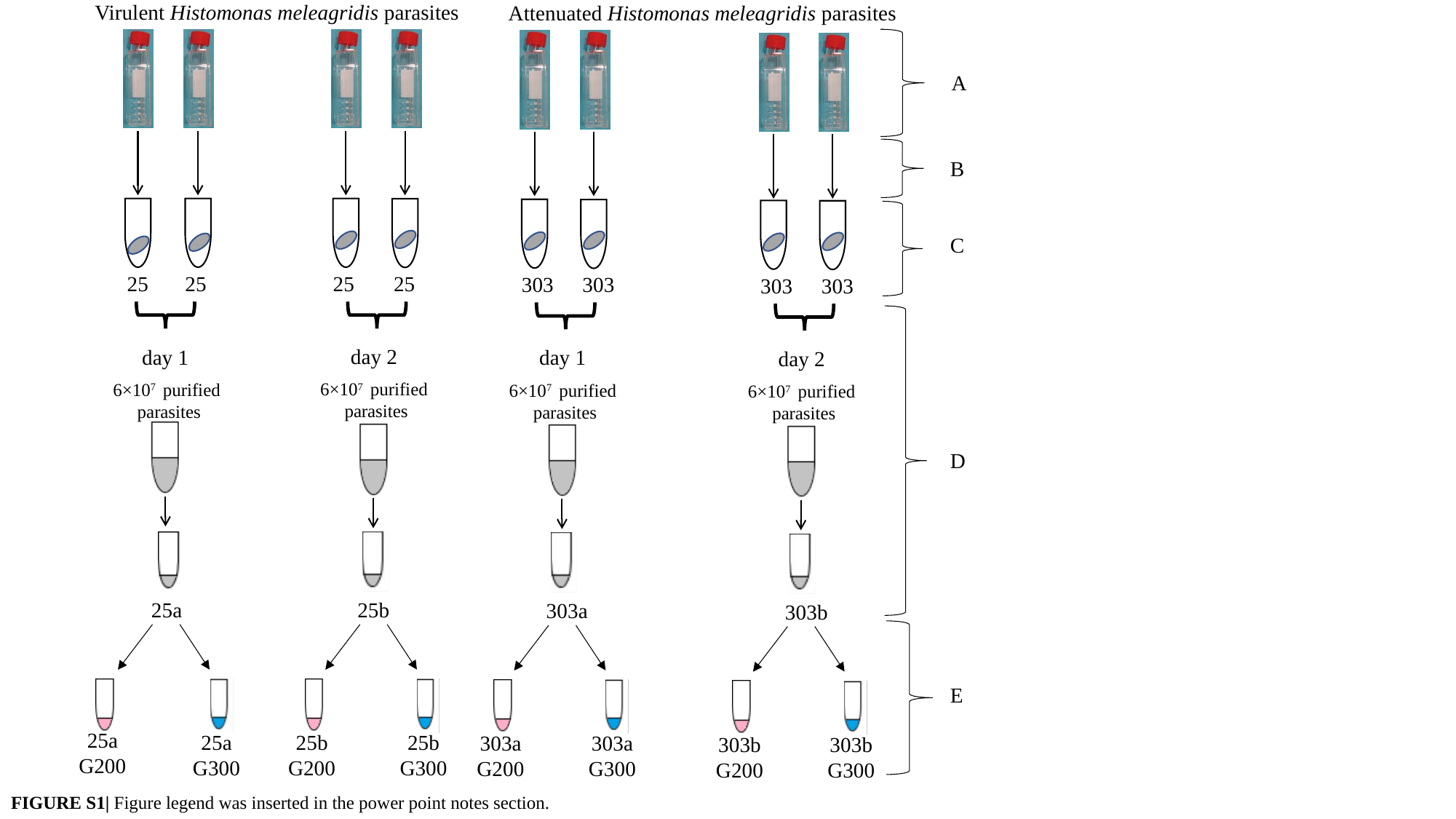

Virulent Histomonas meleagridis parasites
Attenuated Histomonas meleagridis parasites
A
B
25
25
day 1
6×10^7^ purified
 parasites
25a
25a
G200
25a
G300
25
25
day 2
6×10^7^ purified
 parasites
25b
25b
G200
25b
G300
303
303
day 1
6×10^7^ purified
 parasites
303a
303a
G200
303a
G300
303
303
day 2
6×10^7^ purified
 parasites
303b
303b
G200
303b
G300
C
D
E
FIGURE S1| Figure legend was inserted in the power point notes section.
