# Supplementary material for: An Alliance of Gel-Based and Gel-Free Proteomic Techniques Displays Substantial Insight Into the Proteome of a Virulent and an Attenuated Histomonas meleagridis Strain
Source: Front Cell Infect Microbiol. 2018 Nov 16;8:407. doi: 10.3389/fcimb.2018.00407 (PMC6250841; doi:10.3389/fcimb.2018.00407)
Supplement: Supplementary file 5 [file Presentation_2.PPTX]

## Slide 1
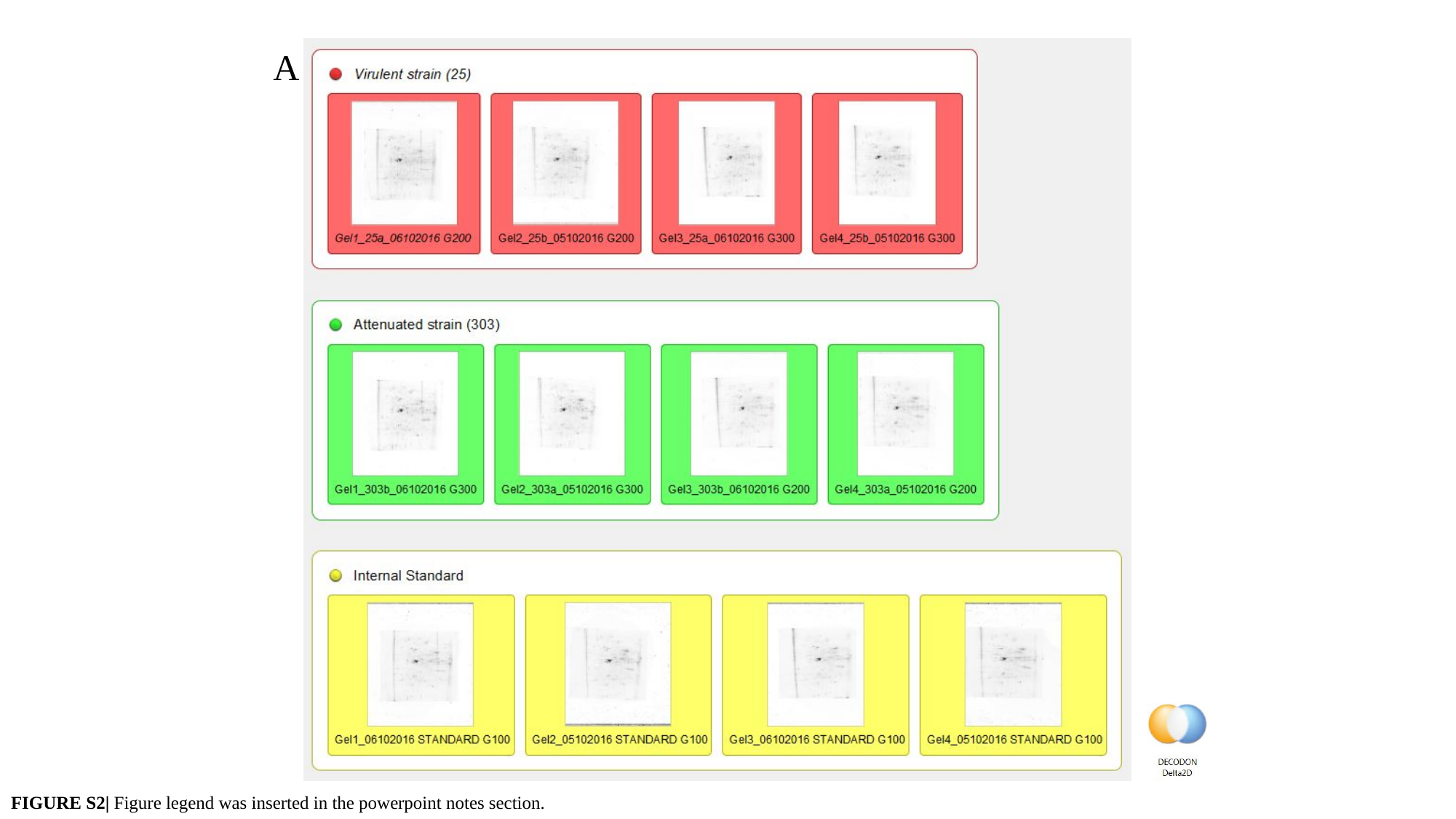

A
FIGURE S2| Figure legend was inserted in the powerpoint notes section.

## Slide 2
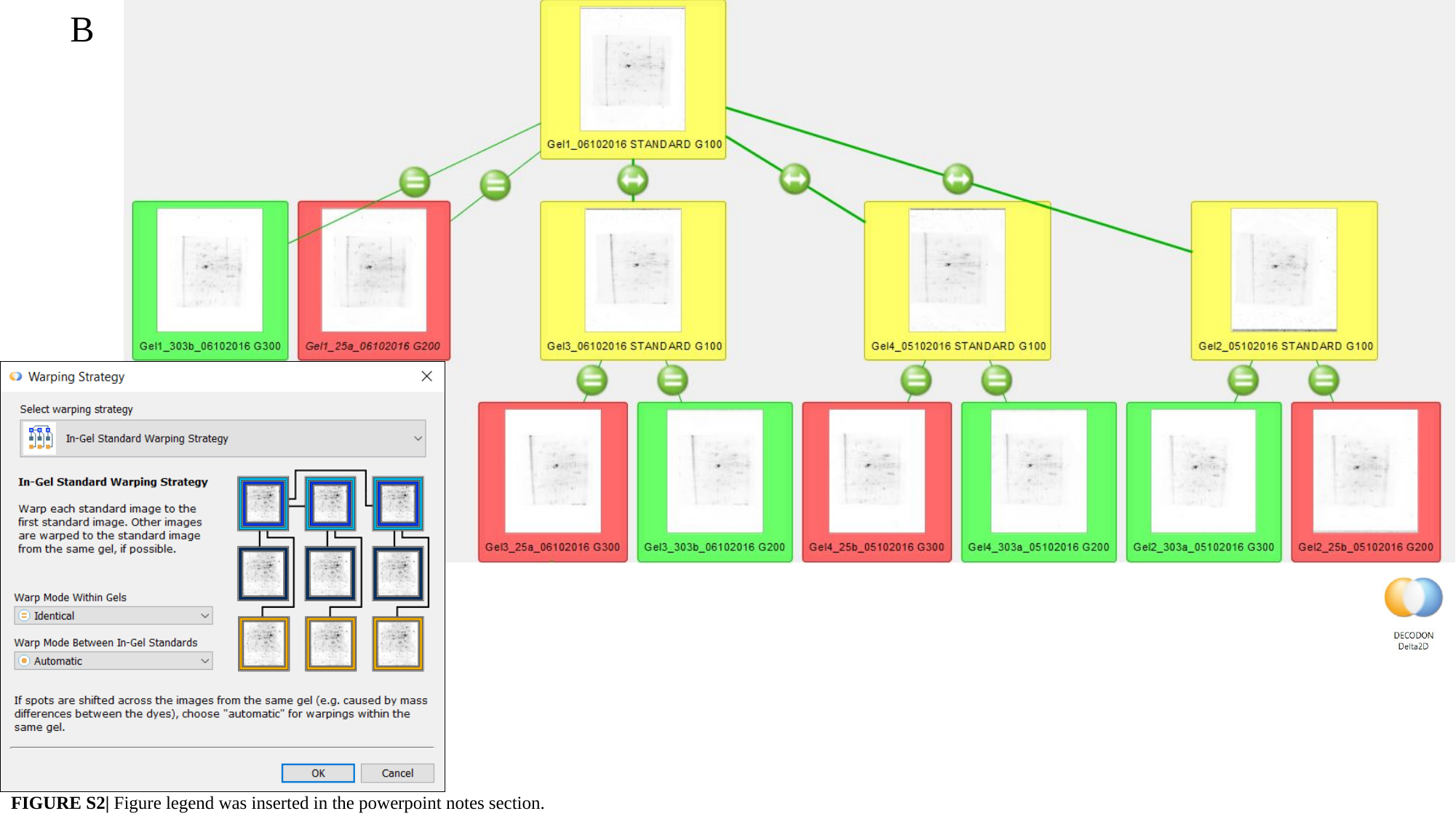

B
FIGURE S2| Figure legend was inserted in the powerpoint notes section.

## Slide 3
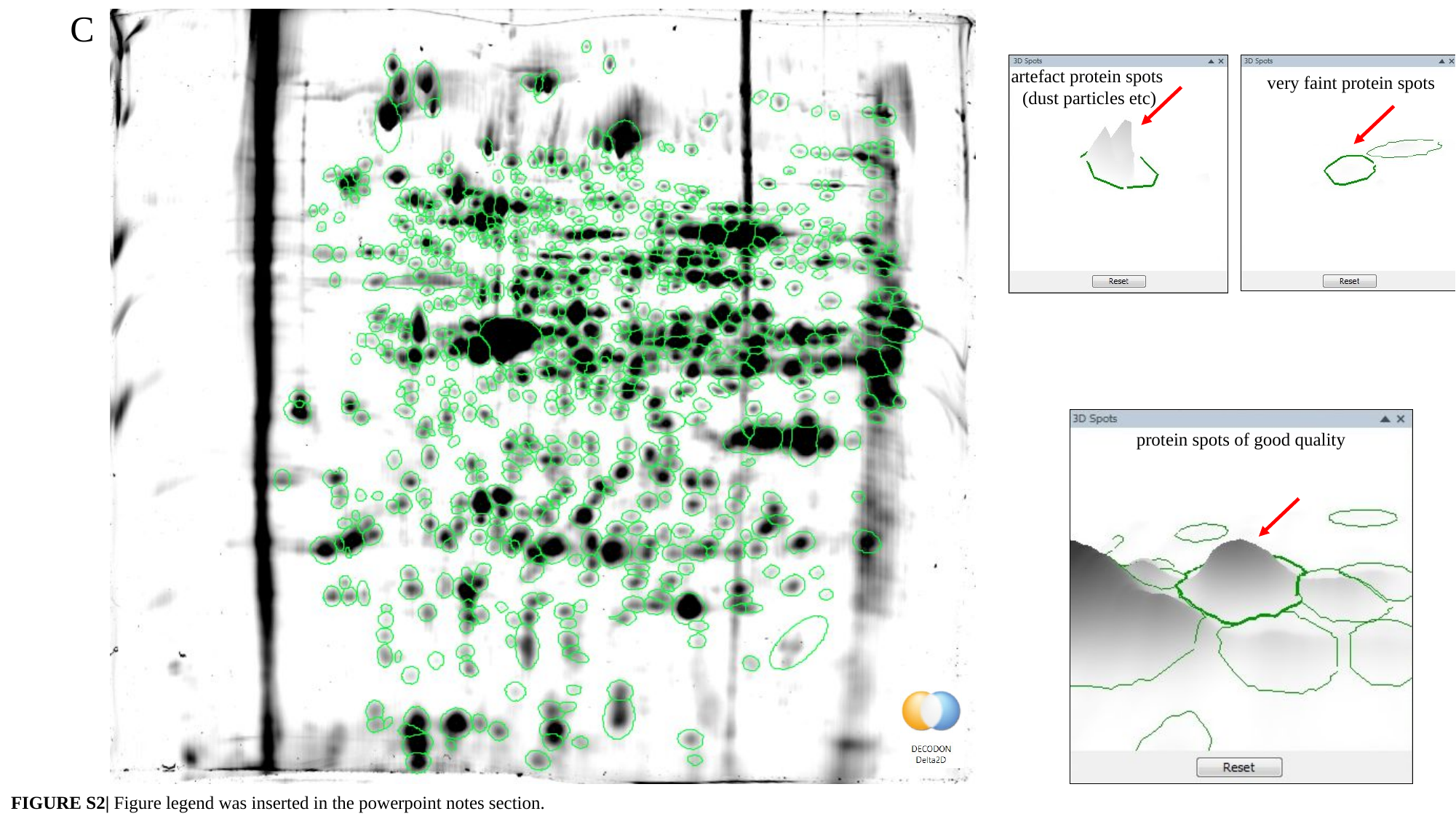

C
artefact protein spots
(dust particles etc)
very faint protein spots
protein spots of good quality
FIGURE S2| Figure legend was inserted in the powerpoint notes section.
