# Supplementary material for: An Alliance of Gel-Based and Gel-Free Proteomic Techniques Displays Substantial Insight Into the Proteome of a Virulent and an Attenuated Histomonas meleagridis Strain
Source: Front Cell Infect Microbiol. 2018 Nov 16;8:407. doi: 10.3389/fcimb.2018.00407 (PMC6250841; doi:10.3389/fcimb.2018.00407)
Supplement: Supplementary file 6 [file Presentation_3.pptx]

## Slide 1
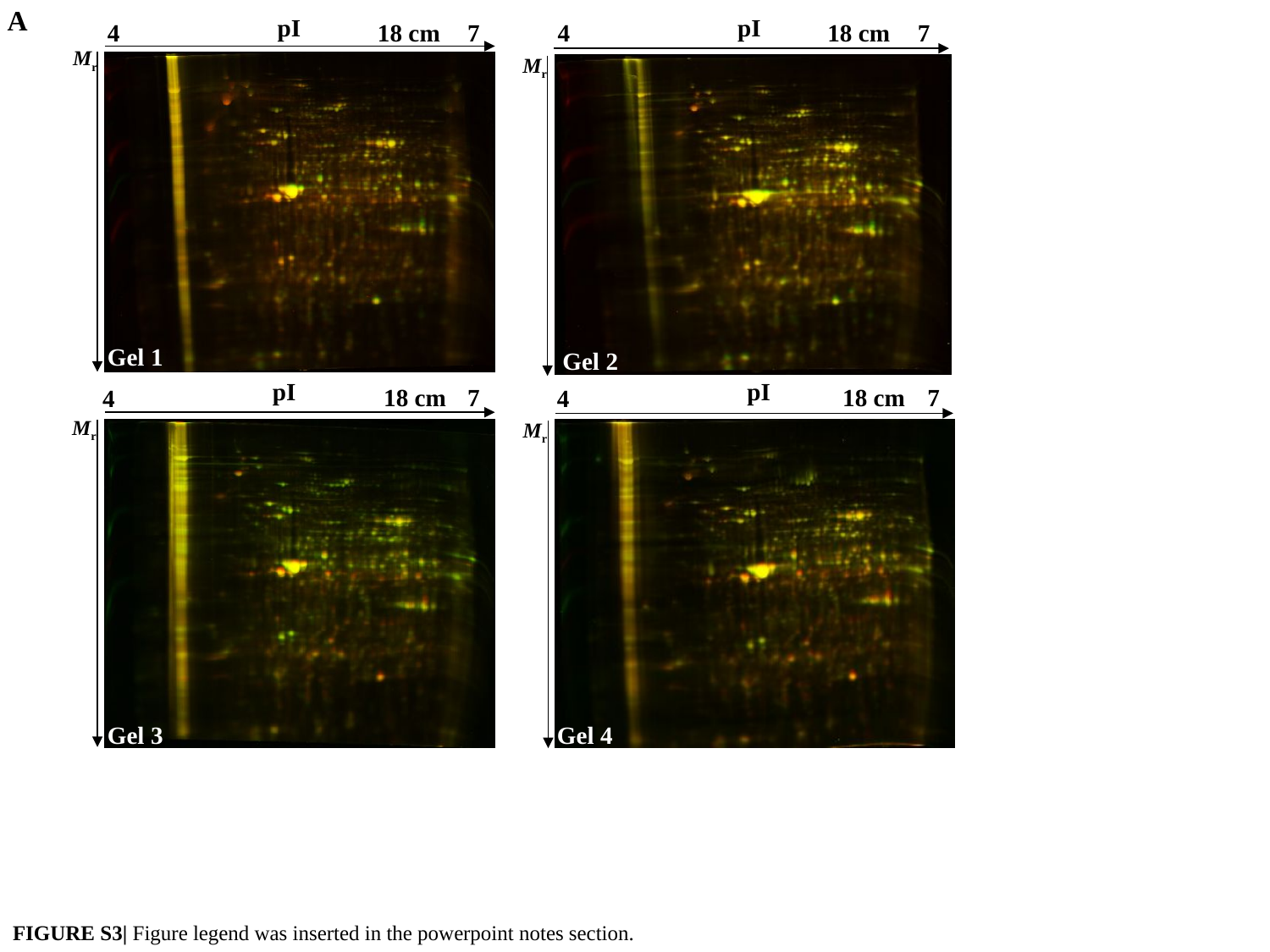

pI
	pI
A
	 Mr
4
18 cm
7
4
18 cm
7
	 Mr
Gel 1
Gel 2
	pI
18 cm
7
4
	pI
7
18 cm
4
	 Mr
	 Mr
Gel 3
Gel 4
FIGURE S3| Figure legend was inserted in the powerpoint notes section.

## Slide 2
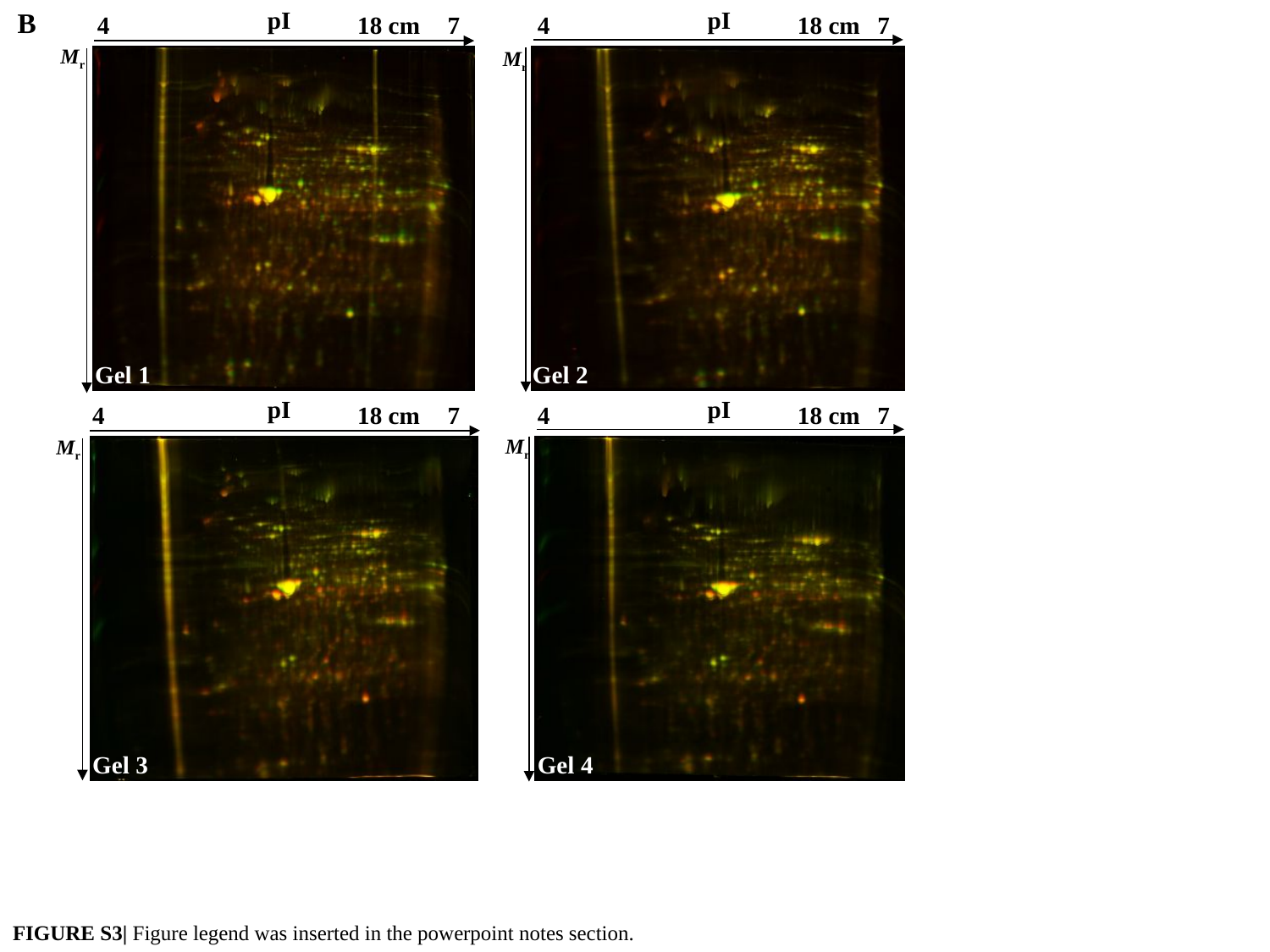

pI
	pI
B
4
18 cm
7
4
18 cm
7
	 Mr
	 Mr
Gel 1
Gel 2
	pI
	pI
4
18 cm
7
4
18 cm
7
	 Mr
	 Mr
Gel 3
Gel 4
FIGURE S3| Figure legend was inserted in the powerpoint notes section.
