# Supplementary material for: An Alliance of Gel-Based and Gel-Free Proteomic Techniques Displays Substantial Insight Into the Proteome of a Virulent and an Attenuated Histomonas meleagridis Strain
Source: Front Cell Infect Microbiol. 2018 Nov 16;8:407. doi: 10.3389/fcimb.2018.00407 (PMC6250841; doi:10.3389/fcimb.2018.00407)
Supplement: Supplementary file 7 [file Presentation_4.pptx]

## Slide 1
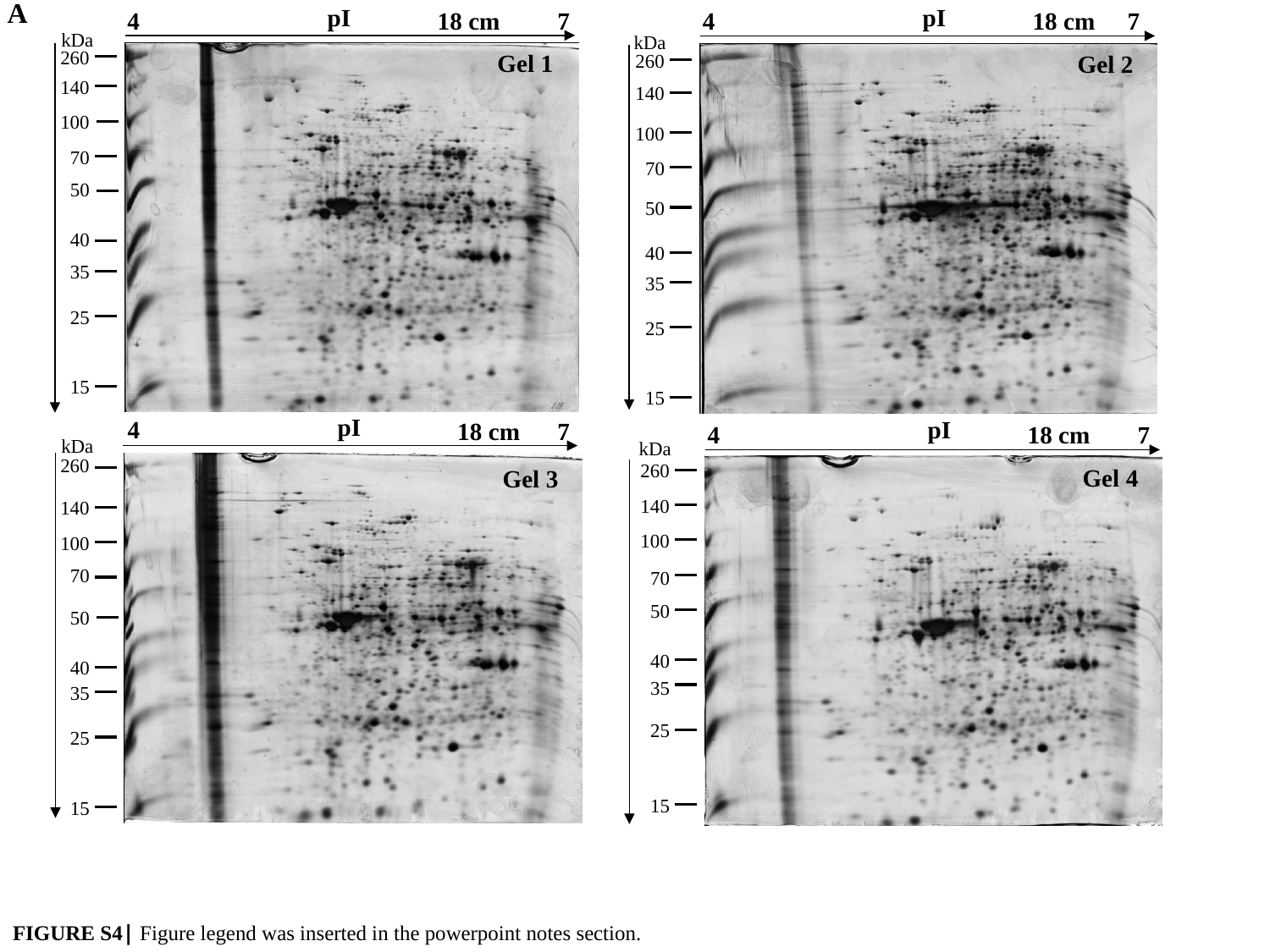

pI
4
18 cm
7
kDa
260
Gel 1
140
100
70
50
40
35
25
15
	pI
4
18 cm
7
kDa
260
140
100
70
50
40
35
25
15
Gel 2
A
	pI
4
18 cm
7
kDa
260
Gel 3
140
100
70
50
40
35
25
15
	pI
4
18 cm
7
260
Gel 4
140
100
70
50
40
35
25
15
kDa
FIGURE S4| Figure legend was inserted in the powerpoint notes section.

## Slide 2
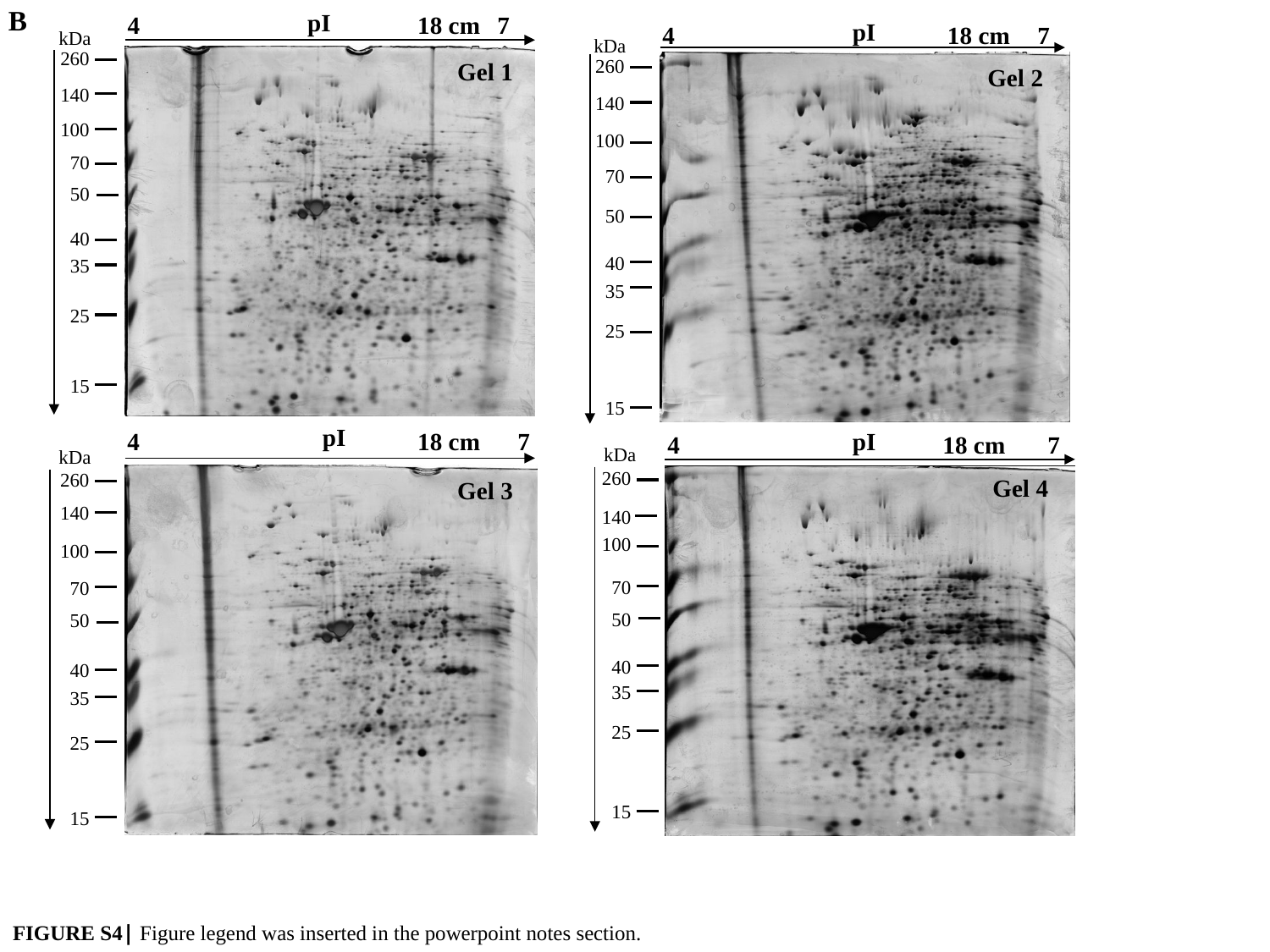

pI
4
18 cm
7
Gel 1
kDa
260
140
100
70
50
40
35
25
15
	pI
4
18 cm
7
Gel 2
260
140
100
70
50
40
35
25
15
kDa
B
	pI
4
18 cm
7
Gel 3
260
140
100
70
50
40
35
25
15
kDa
	pI
4
18 cm
7
Gel 4
kDa
260
140
100
70
50
40
35
25
15
FIGURE S4| Figure legend was inserted in the powerpoint notes section.
