# Supplementary material for: An Alliance of Gel-Based and Gel-Free Proteomic Techniques Displays Substantial Insight Into the Proteome of a Virulent and an Attenuated Histomonas meleagridis Strain
Source: Front Cell Infect Microbiol. 2018 Nov 16;8:407. doi: 10.3389/fcimb.2018.00407 (PMC6250841; doi:10.3389/fcimb.2018.00407)
Supplement: Supplementary file 8 [file Presentation_5.PPTX]

## Slide 1
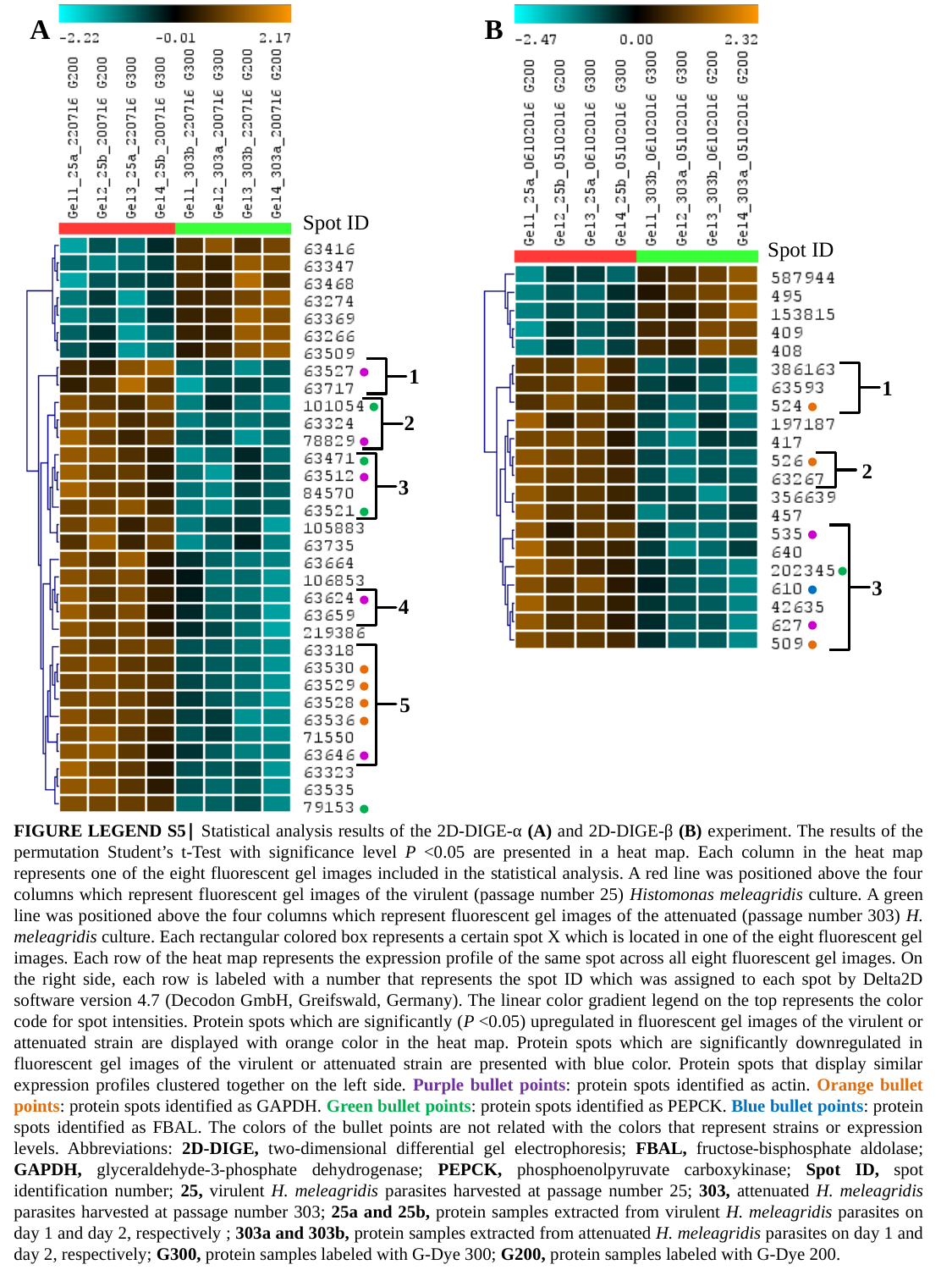

A
•
•
•
•
•
•
•
•
•
•
•
5
•
1
2
3
4
•
•
•
•
•
•
•
•
1
2
3
B
Spot ID
Spot ID
•
*
FIGURE LEGEND S5| Statistical analysis results of the 2D-DIGE-α (A) and 2D-DIGE-β (B) experiment. The results of the permutation Student’s t-Test with significance level P ˂0.05 are presented in a heat map. Each column in the heat map represents one of the eight fluorescent gel images included in the statistical analysis. A red line was positioned above the four columns which represent fluorescent gel images of the virulent (passage number 25) Histomonas meleagridis culture. A green line was positioned above the four columns which represent fluorescent gel images of the attenuated (passage number 303) H. meleagridis culture. Each rectangular colored box represents a certain spot X which is located in one of the eight fluorescent gel images. Each row of the heat map represents the expression profile of the same spot across all eight fluorescent gel images. On the right side, each row is labeled with a number that represents the spot ID which was assigned to each spot by Delta2D software version 4.7 (Decodon GmbH, Greifswald, Germany). The linear color gradient legend on the top represents the color code for spot intensities. Protein spots which are significantly (P ˂0.05) upregulated in fluorescent gel images of the virulent or attenuated strain are displayed with orange color in the heat map. Protein spots which are significantly downregulated in fluorescent gel images of the virulent or attenuated strain are presented with blue color. Protein spots that display similar expression profiles clustered together on the left side. Purple bullet points: protein spots identified as actin. Orange bullet points: protein spots identified as GAPDH. Green bullet points: protein spots identified as PEPCK. Blue bullet points: protein spots identified as FBAL. The colors of the bullet points are not related with the colors that represent strains or expression levels. Abbreviations: 2D-DIGE, two-dimensional differential gel electrophoresis; FBAL, fructose-bisphosphate aldolase; GAPDH, glyceraldehyde-3-phosphate dehydrogenase; PEPCK, phosphoenolpyruvate carboxykinase; Spot ID, spot identification number; 25, virulent H. meleagridis parasites harvested at passage number 25; 303, attenuated H. meleagridis parasites harvested at passage number 303; 25a and 25b, protein samples extracted from virulent H. meleagridis parasites on day 1 and day 2, respectively ; 303a and 303b, protein samples extracted from attenuated H. meleagridis parasites on day 1 and day 2, respectively; G300, protein samples labeled with G-Dye 300; G200, protein samples labeled with G-Dye 200.
