# Supplementary material for: An Alliance of Gel-Based and Gel-Free Proteomic Techniques Displays Substantial Insight Into the Proteome of a Virulent and an Attenuated Histomonas meleagridis Strain
Source: Front Cell Infect Microbiol. 2018 Nov 16;8:407. doi: 10.3389/fcimb.2018.00407 (PMC6250841; doi:10.3389/fcimb.2018.00407)
Supplement: Supplementary file 10 [file Presentation_7.pptx]

## Slide 1
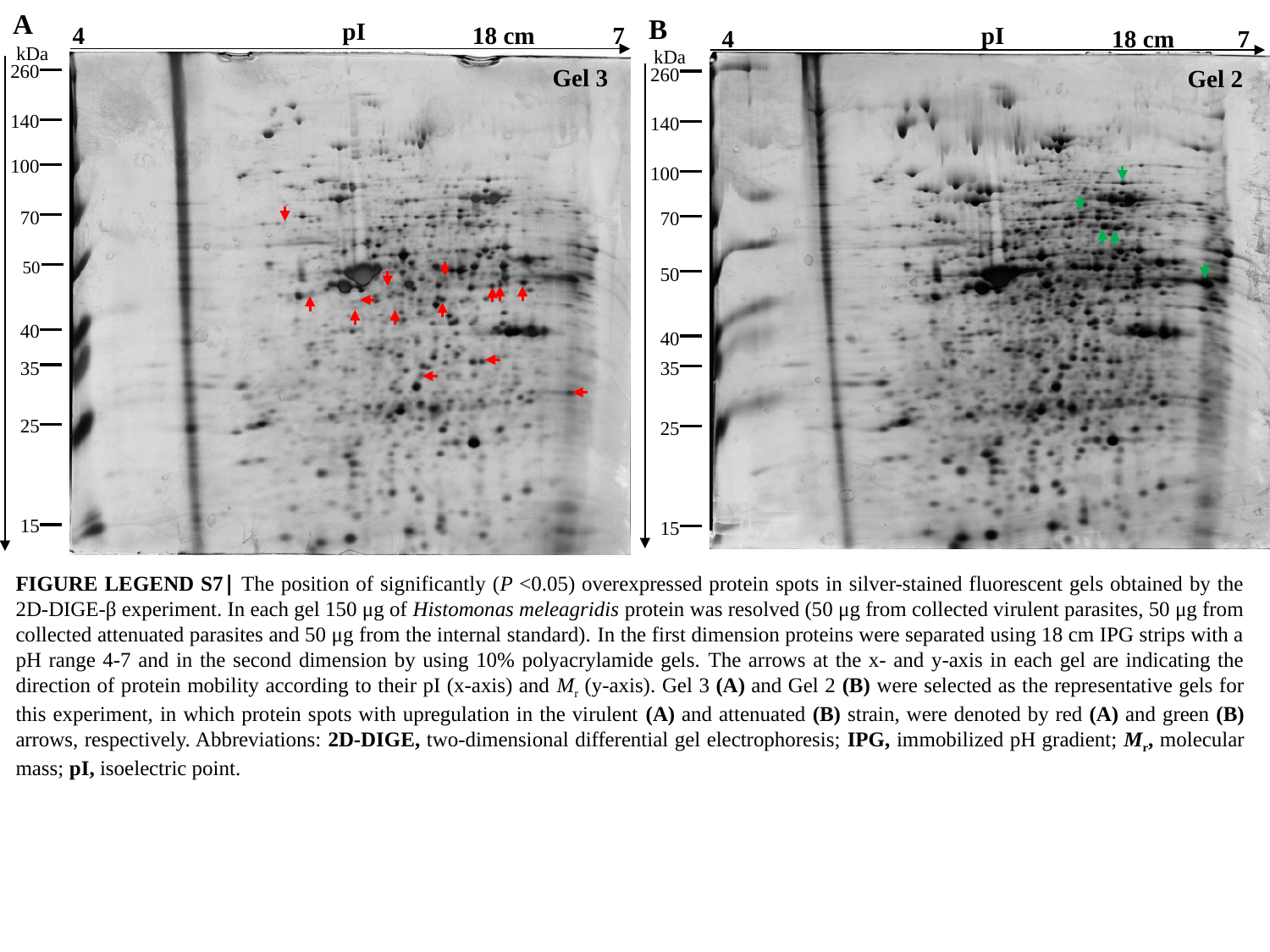

A
	pI
4
18 cm
7
260
Gel 3
140
100
70
50
40
35
25
15
kDa
	pI
4
18 cm
7
260
Gel 2
140
100
70
50
40
35
25
15
kDa
B
FIGURE LEGEND S7| The position of significantly (P ˂0.05) overexpressed protein spots in silver-stained fluorescent gels obtained by the 2D-DIGE-β experiment. In each gel 150 μg of Histomonas meleagridis protein was resolved (50 μg from collected virulent parasites, 50 μg from collected attenuated parasites and 50 μg from the internal standard). In the first dimension proteins were separated using 18 cm IPG strips with a pH range 4-7 and in the second dimension by using 10% polyacrylamide gels. The arrows at the x- and y-axis in each gel are indicating the direction of protein mobility according to their pI (x-axis) and Mr (y-axis). Gel 3 (A) and Gel 2 (B) were selected as the representative gels for this experiment, in which protein spots with upregulation in the virulent (A) and attenuated (B) strain, were denoted by red (A) and green (B) arrows, respectively. Abbreviations: 2D-DIGE, two-dimensional differential gel electrophoresis; IPG, immobilized pH gradient; Mr, molecular mass; pI, isoelectric point.
