# Supplementary material for: An Alliance of Gel-Based and Gel-Free Proteomic Techniques Displays Substantial Insight Into the Proteome of a Virulent and an Attenuated Histomonas meleagridis Strain
Source: Front Cell Infect Microbiol. 2018 Nov 16;8:407. doi: 10.3389/fcimb.2018.00407 (PMC6250841; doi:10.3389/fcimb.2018.00407)
Supplement: Supplementary file 11 [file Presentation_8.pptx]

## Slide 1
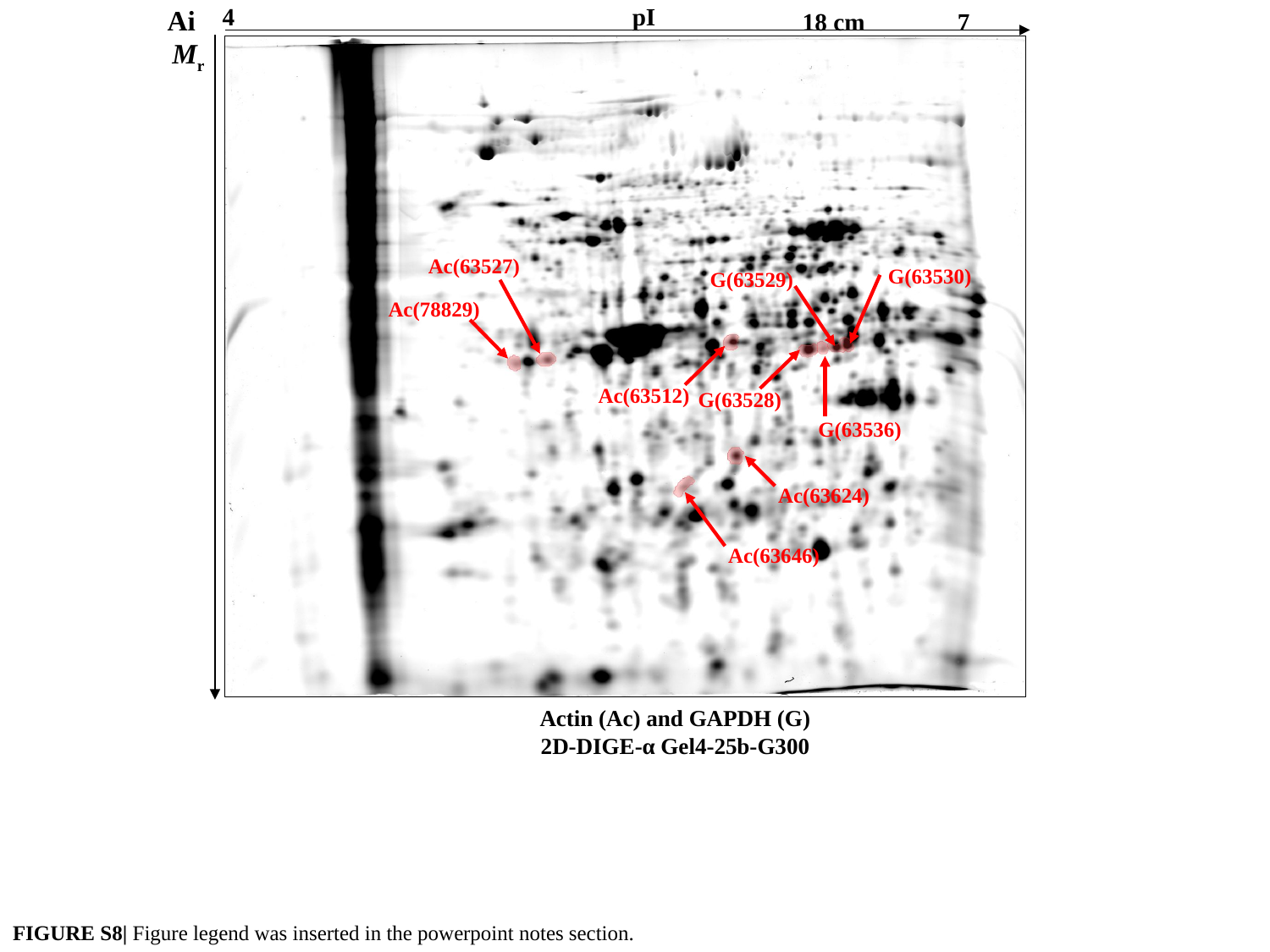

18 cm
7
	pI
4
Ac(63527)
G(63530)
G(63529)
Ac(78829)
Ac(63512)
G(63528)
G(63536)
Ac(63624)
Ac(63646)
Actin (Ac) and GAPDH (G)
2D-DIGE-α Gel4-25b-G300
Ai
Mr
FIGURE S8| Figure legend was inserted in the powerpoint notes section.

## Slide 2
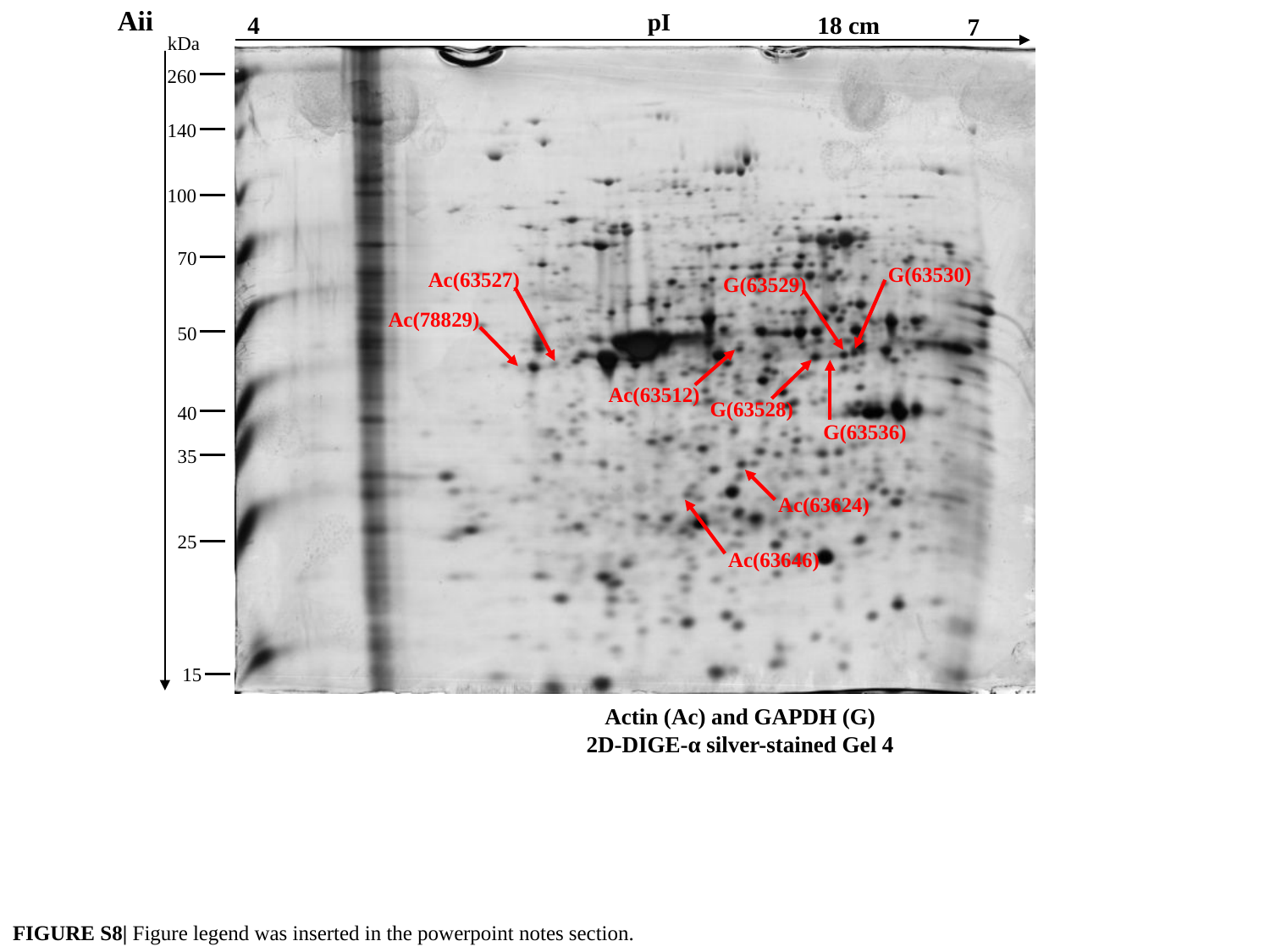

pI
4
18 cm
7
kDa
260
140
100
70
G(63530)
Ac(63527)
G(63529)
Ac(78829)
50
Ac(63512)
G(63528)
40
G(63536)
35
Ac(63624)
25
Ac(63646)
15
Actin (Ac) and GAPDH (G)
2D-DIGE-α silver-stained Gel 4
Aii
FIGURE S8| Figure legend was inserted in the powerpoint notes section.

## Slide 3
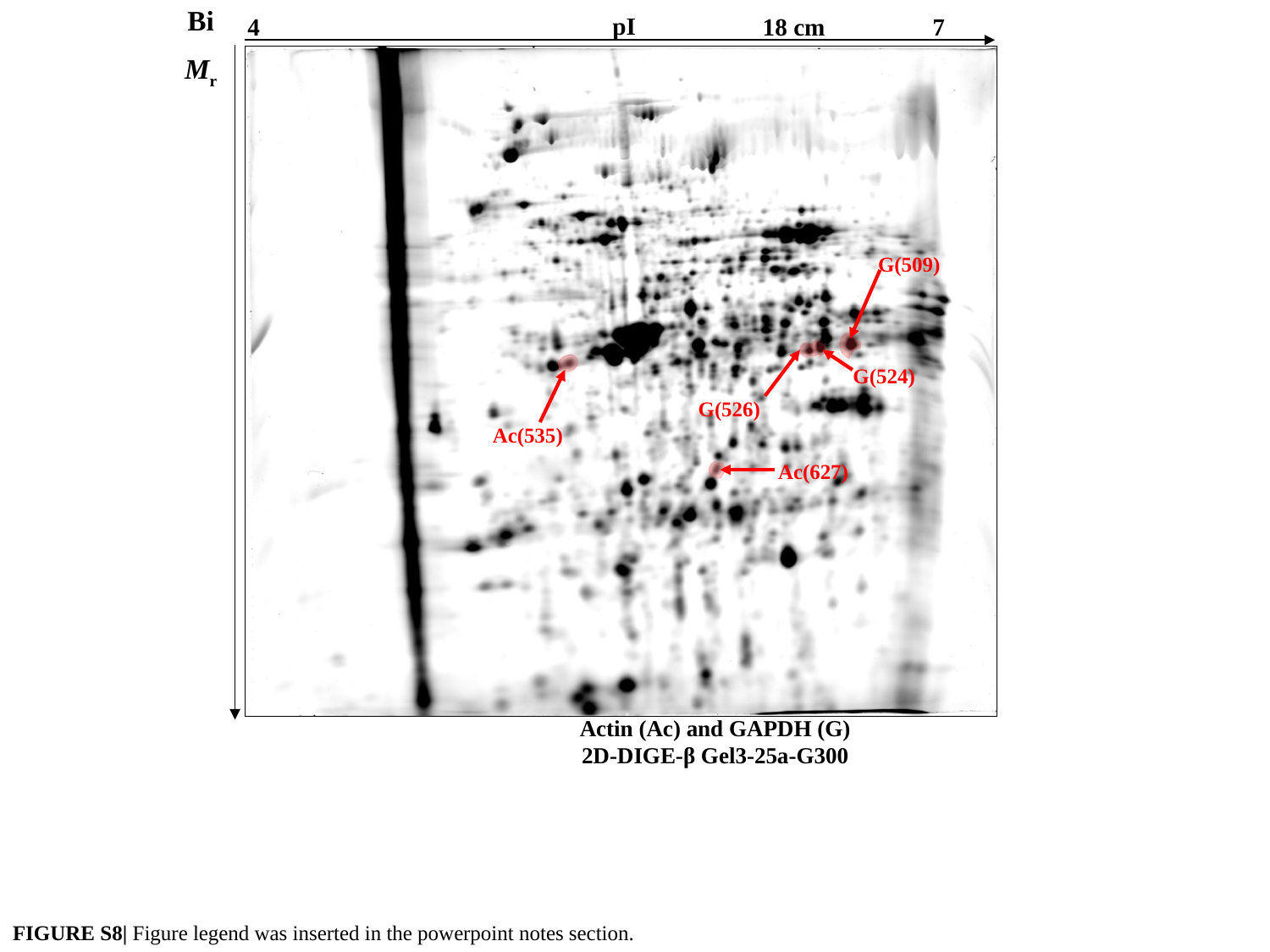

pI
4
18 cm
7
G(509)
G(524)
G(526)
Ac(535)
Ac(627)
Actin (Ac) and GAPDH (G)
2D-DIGE-β Gel3-25a-G300
Bi
Mr
FIGURE S8| Figure legend was inserted in the powerpoint notes section.

## Slide 4
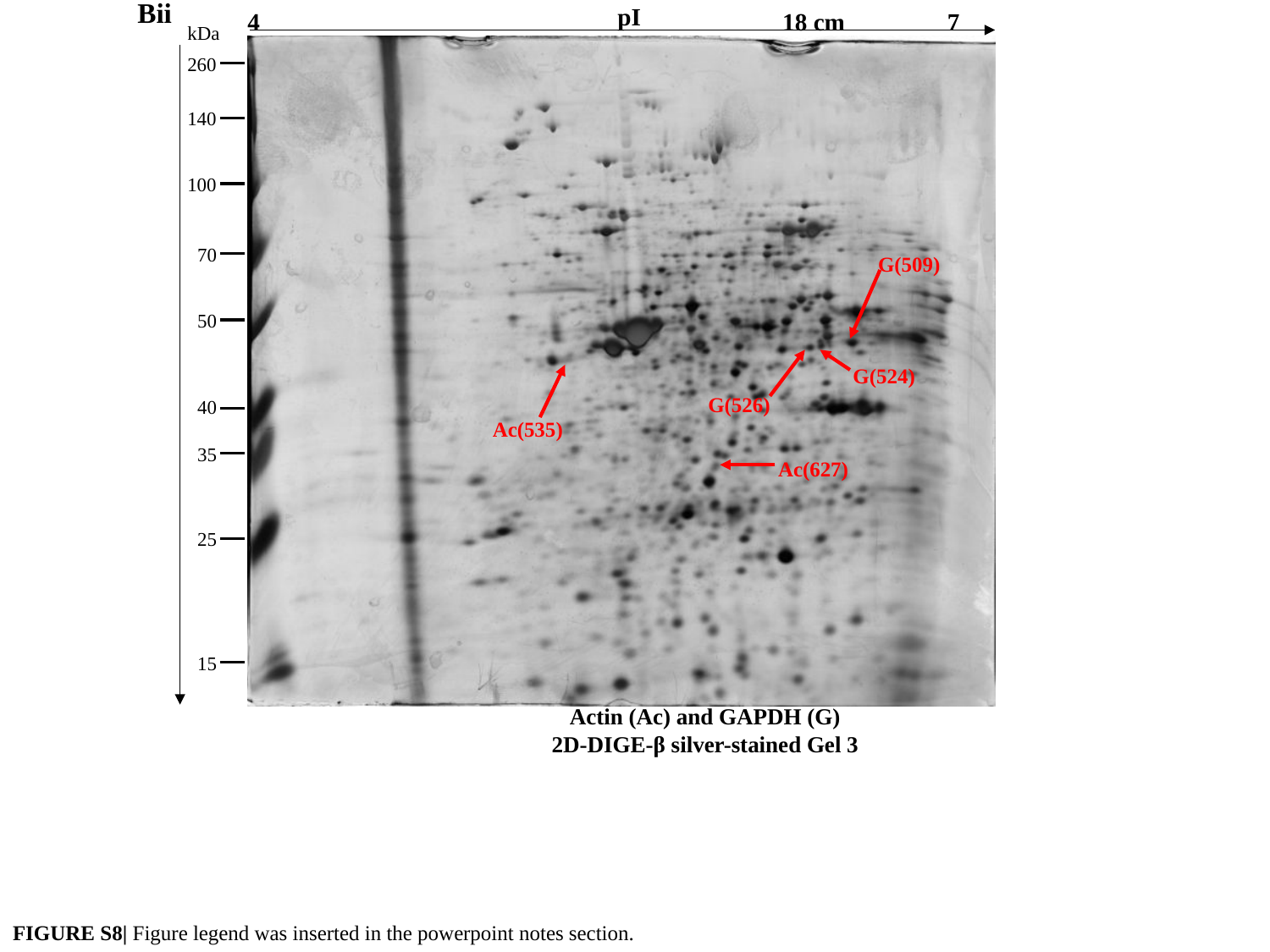

4
18 cm
7
	pI
kDa
260
140
100
70
G(509)
50
G(524)
G(526)
40
Ac(535)
35
Ac(627)
25
15
Actin (Ac) and GAPDH (G)
2D-DIGE-β silver-stained Gel 3
Bii
FIGURE S8| Figure legend was inserted in the powerpoint notes section.
