# Supplementary material for: An Alliance of Gel-Based and Gel-Free Proteomic Techniques Displays Substantial Insight Into the Proteome of a Virulent and an Attenuated Histomonas meleagridis Strain
Source: Front Cell Infect Microbiol. 2018 Nov 16;8:407. doi: 10.3389/fcimb.2018.00407 (PMC6250841; doi:10.3389/fcimb.2018.00407)
Supplement: Supplementary file 12 [file Presentation_9.pptx]

## Slide 1
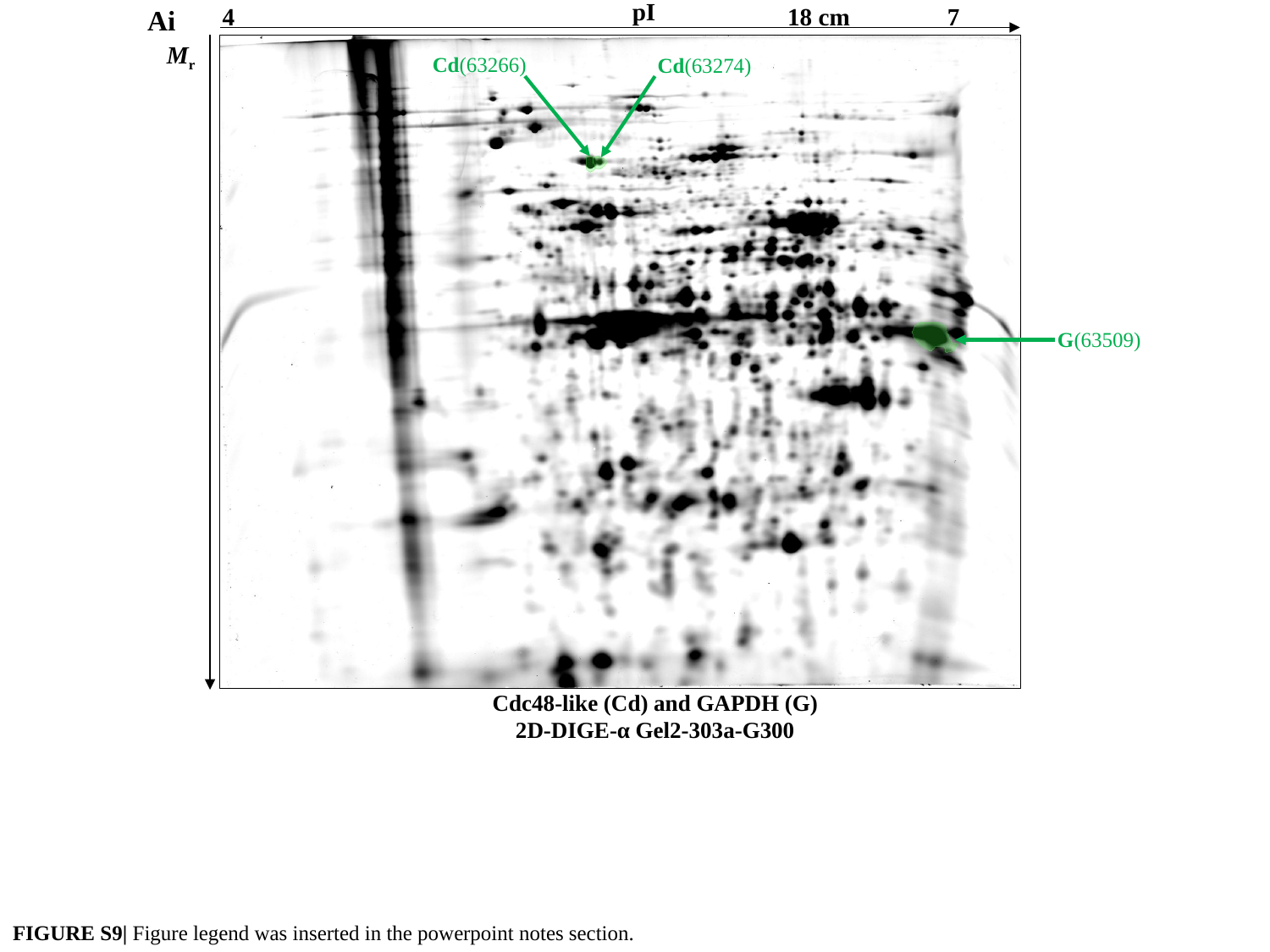

pI
4
18 cm
7
Cd(63266)
Cd(63274)
G(63509)
Cdc48-like (Cd) and GAPDH (G)
2D-DIGE-α Gel2-303a-G300
Ai
Mr
FIGURE S9| Figure legend was inserted in the powerpoint notes section.

## Slide 2
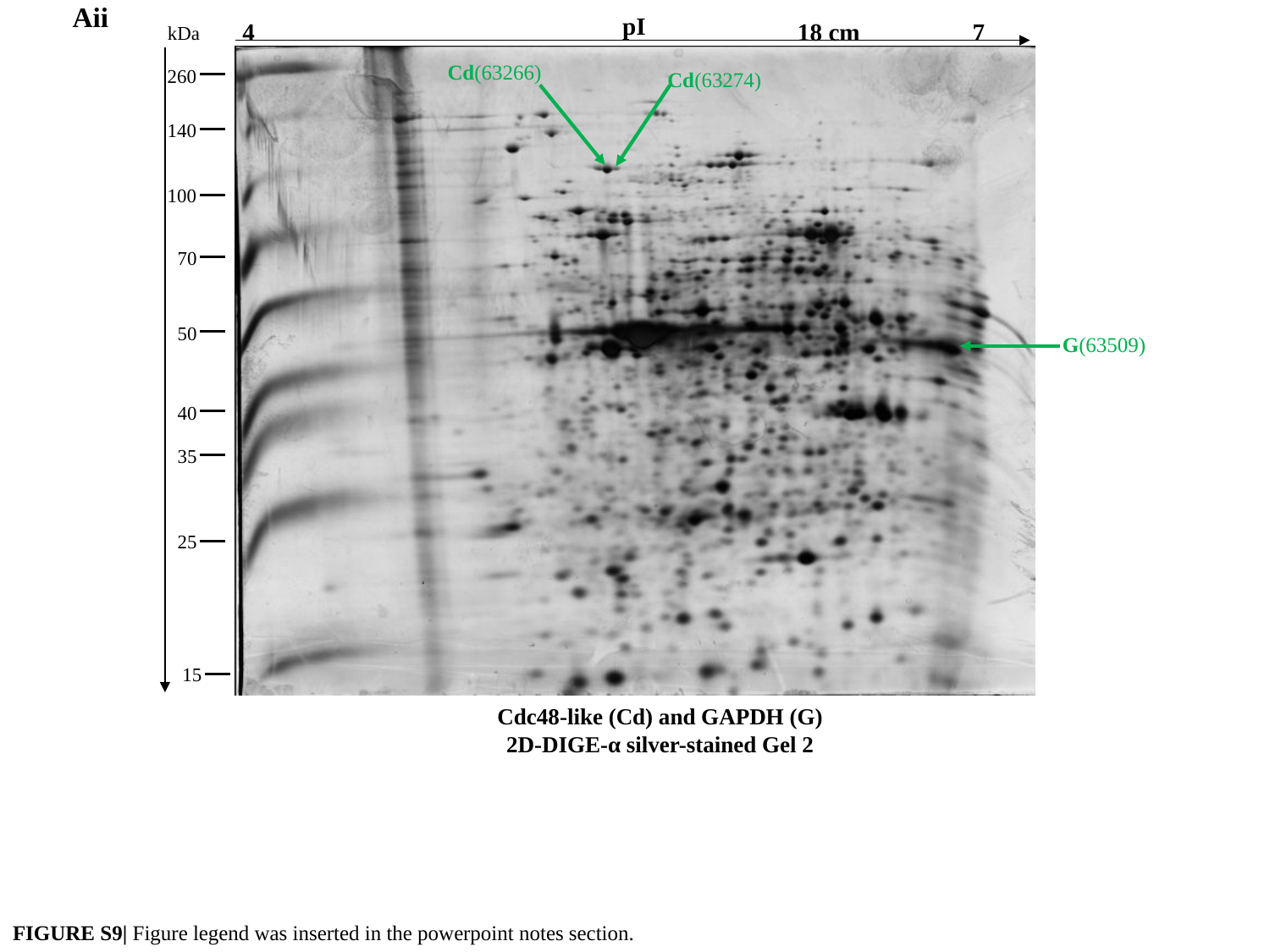

pI
4
18 cm
7
kDa
Cd(63266)
260
Cd(63274)
140
100
70
50
G(63509)
40
35
25
15
Cdc48-like (Cd) and GAPDH (G)
2D-DIGE-α silver-stained Gel 2
Aii
FIGURE S9| Figure legend was inserted in the powerpoint notes section.

## Slide 3
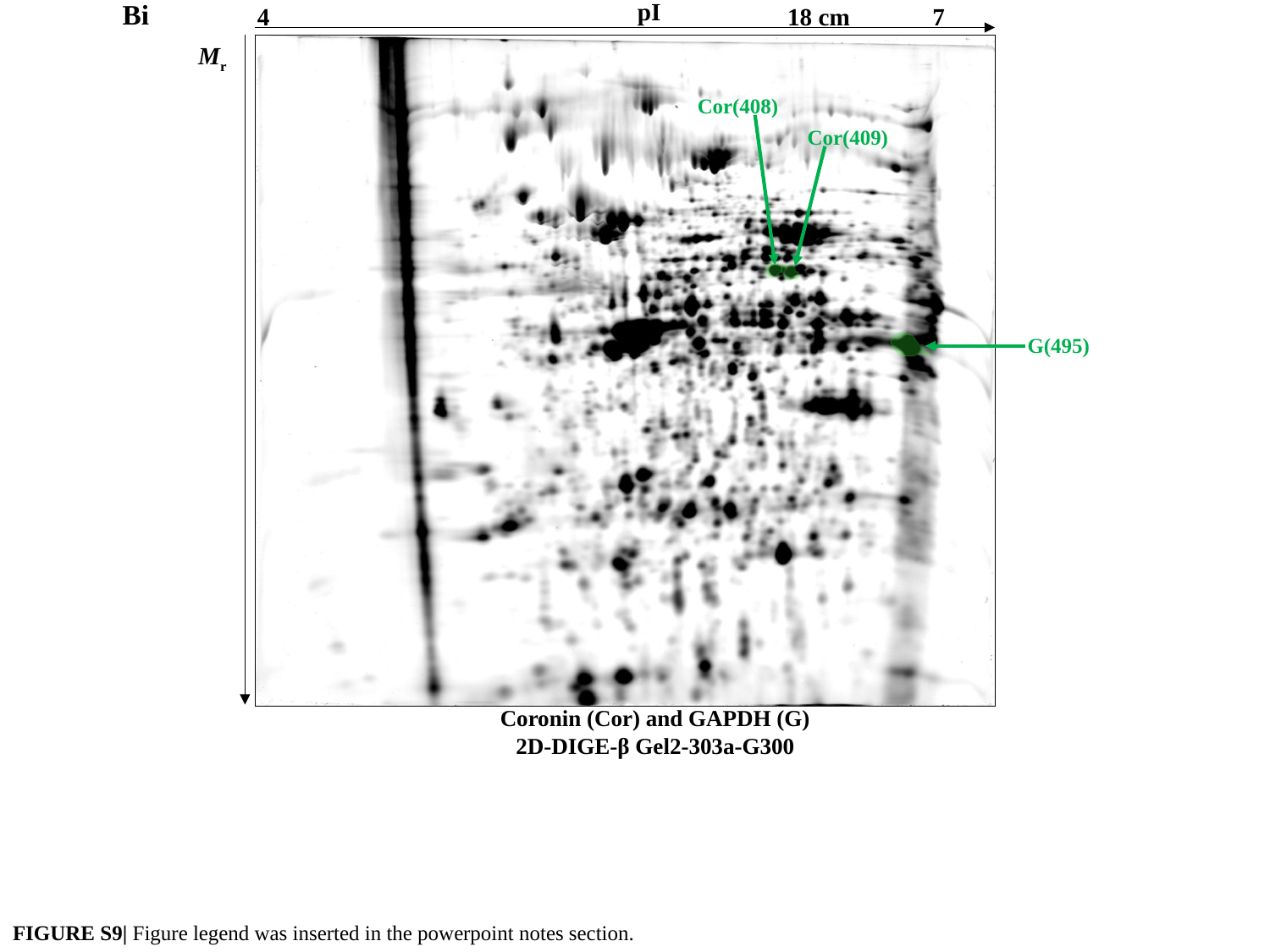

pI
4
18 cm
7
Cor(408)
Cor(409)
G(495)
Coronin (Cor) and GAPDH (G)
2D-DIGE-β Gel2-303a-G300
Bi
Mr
FIGURE S9| Figure legend was inserted in the powerpoint notes section.

## Slide 4
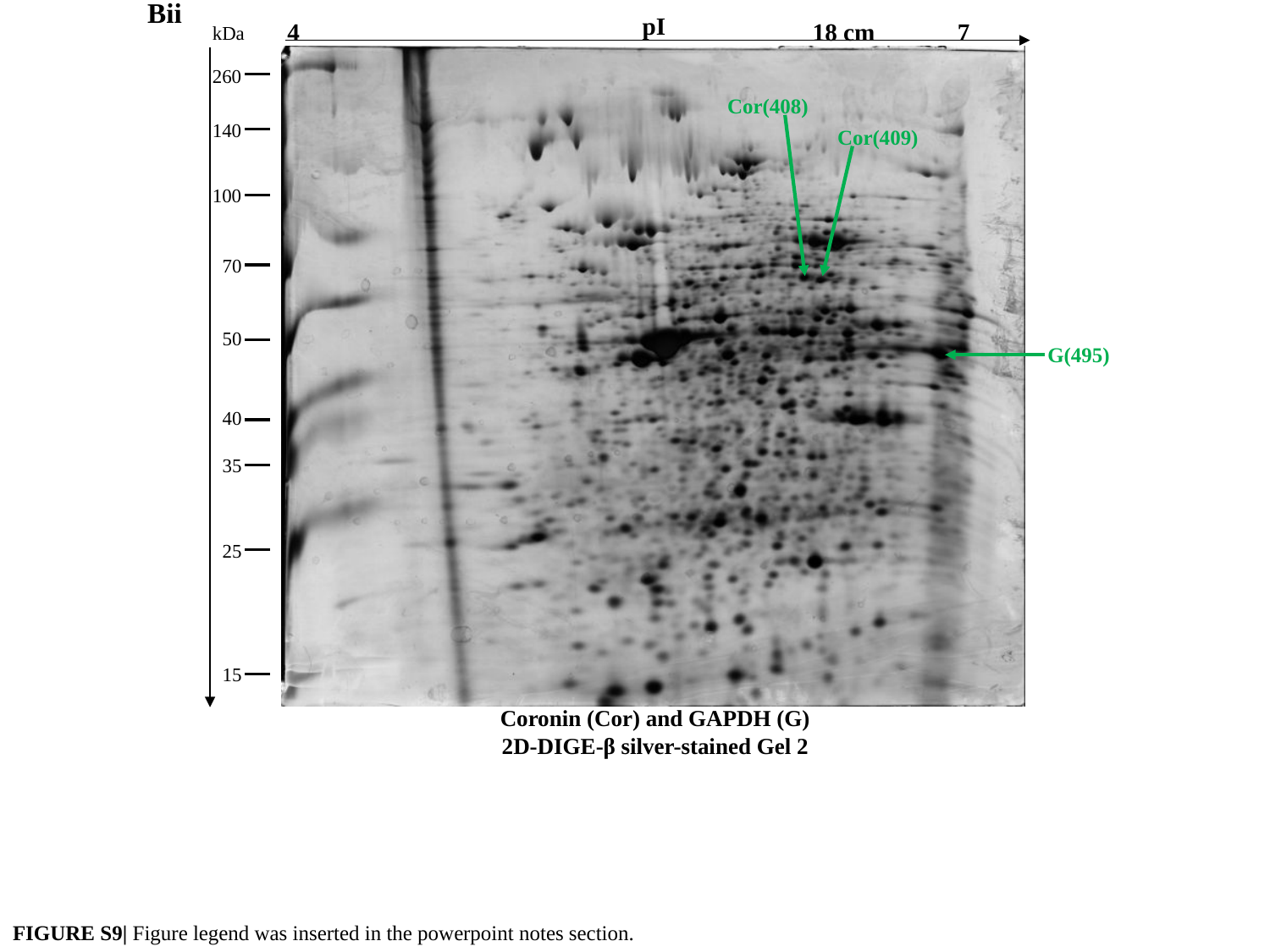

pI
4
18 cm
7
kDa
260
Cor(408)
140
Cor(409)
100
70
50
G(495)
40
35
25
15
Coronin (Cor) and GAPDH (G)
2D-DIGE-β silver-stained Gel 2
Bii
FIGURE S9| Figure legend was inserted in the powerpoint notes section.
